# Ru(ii)-catalyzed C6-selective C–H acylmethylation of pyridones using sulfoxonium ylides as carbene precursors†

**DOI:** 10.1039/c9ra10749e

**Published:** 2020-02-11

**Authors:** Yangjie Fu, Zhaohui Wang, Qiyu Zhang, Zhiyu Li, Hong Liu, Xiaoling Bi, Jiang Wang

**Affiliations:** Jiangsu Key Laboratory of Drug Design and Optimization, Department of Medicinal Chemistry, China Pharmaceutical University 24 Tongjiaxiang Nanjing 210009 China bxl@163.com; State Key Laboratory of Drug Research, Key Laboratory of Receptor Research, Shanghai Institute of Materia Medica, Chinese Academy of Sciences 555 Zu Chong Zhi Road Shanghai 201203 China jwang@simm.ac.cn

## Abstract

In this study, we describe a method using sulfoxonium ylides as carbene precursors to achieve C6-selective acylmethylation of pyridones catalyzed by a ruthenium(ii) complex. This approach featured mild reaction conditions, moderate to excellent yields, high step economy, and had excellent functional group tolerance with good site selectivity. Besides, gram-scale preparation, synthetic utility, and mechanistic studies were conducted. It offers a direct and efficient way to synthesize pyridone derivatives.

## Abstract 2

In this study, we describe a method using sulfoxonium ylides as carbene precursors to achieve C6-selective acylmethylation of pyridones catalyzed by a ruthenium(ii) complex.

## Graphical abstract

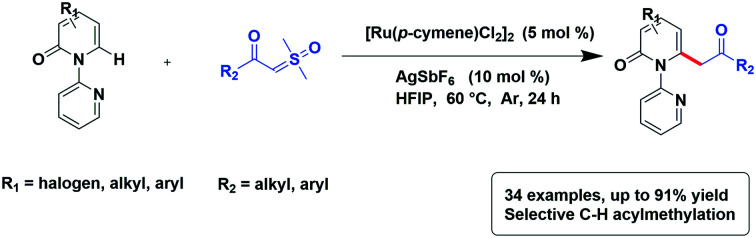

## 1 Introduction

**Fig. 1:**
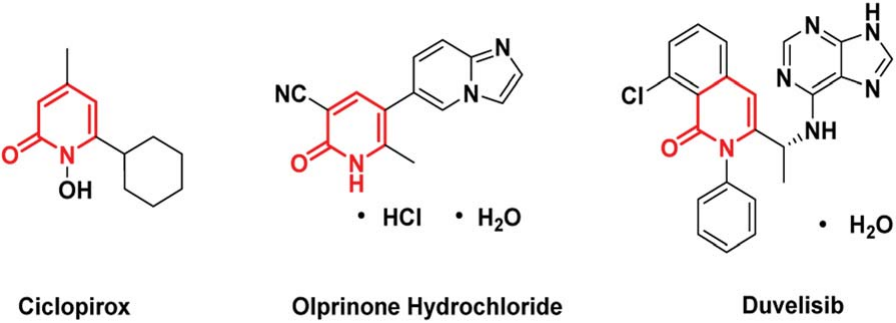
Pharmaceuticals containing a C6-alkylated pyridone core structure.

Pyridone is exhibited as a privilege scaffold in a large range of biological active agents, attracting much attention from medicinal chemists (Fig. 1).^1^ Consequently, how to achieve the late stage functionalization of pyridone has attracted intensive attention.

**Scheme 1:**
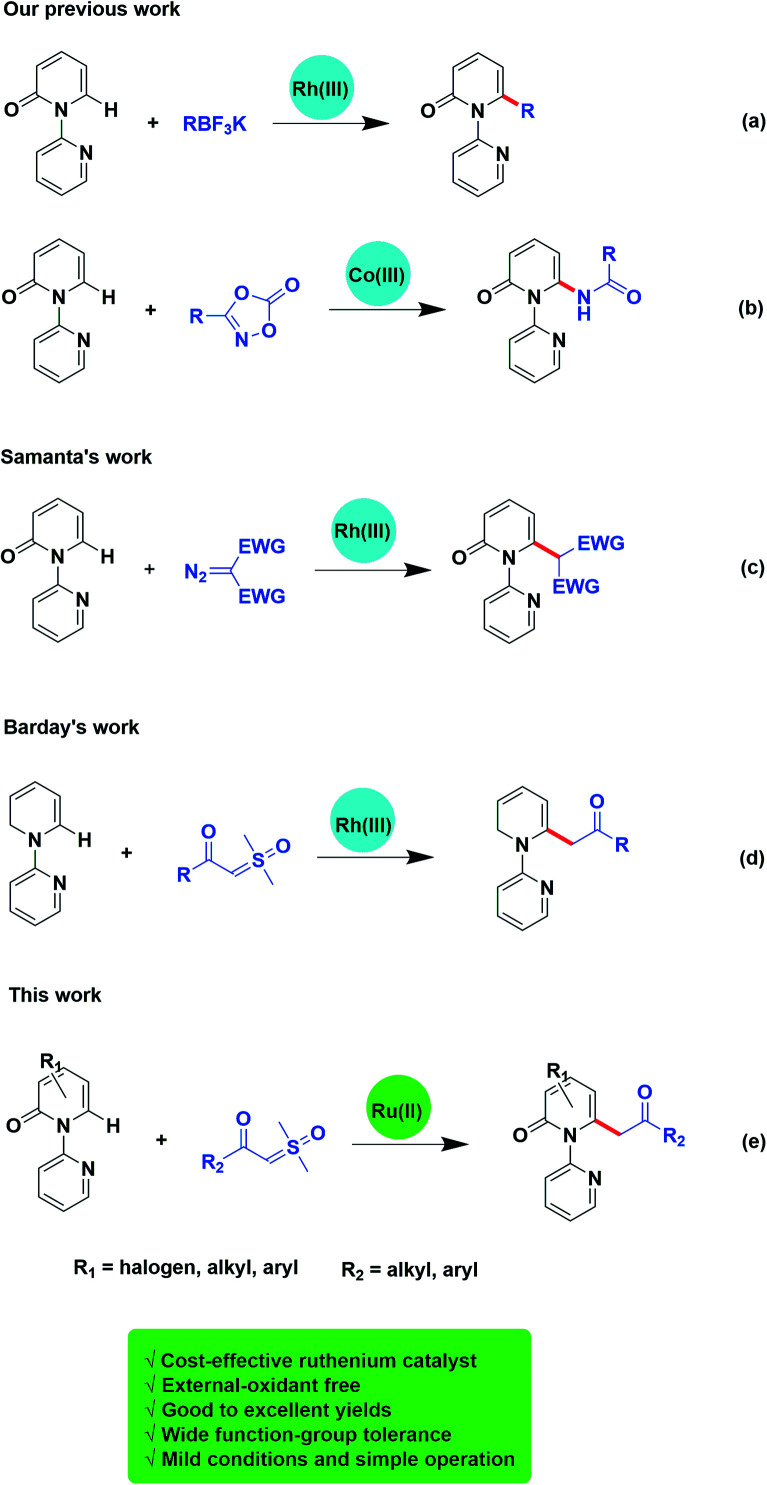
C6-selective C–H functionalization of pyridones.

Traditionally, the direct alkylation of pyridone was usually afforded by pre-functionalization with a halogen followed by transition-metal catalyzed coupling reactions. Recently, the direct C–H functionalization strategy to form C–C or C–X bonds has become a more effective and reliable synthetic route.^2^ Transition-metal-promoted C3 (ref. 3) and C5 (ref. 4) positions of 2-pyridones have been probed exhaustively owing to the sufficient electron density of C–H bonds in these positions. However, only limited examples have been reported on the direct C–H bond functionalization on C6 position of pyridone.^5^ For instance, Cramer and collaborators described the synthesis of 1,6-annulated 2-pyridones by selective intramolecular nickel catalyzed cyclization.^5*c*^ Afterwards, more C–H functionalization at C6 position of pyridone mediated by transition-metal have been reported.^6^ Miura and colleagues exploited selective C6 borylation of pyridone with bis(pinacolato)diboron *via* rhodium catalyzed C–H bond activation. The synthetic utility has been extended by subsequent Suzuki–Miyaura cross-coupling to form new C–C bonds and after removal of the directing group, the C6-arylated NH-pyridone has been afforded.^6*c*^ At the same time, our group has successively reported the rhodium or cobalt-catalyzed, C6-selective C–H alkylation, arylation, and amidation of pyridones by using potassium trifluoroborates or oxazolones (Scheme 1a and b).^6*d*,6*h*^

Transition-metal-catalyzed C–H functionalization is based on carbene migratory insertion to achieve the transformation. In transition-metal-catalyzed C–H functionalization, α-diazo carbonyls are commonly used as carbene precursor.^7,8^ Samanta and colleagues disclosed a rhodium-mediated C6-selective alkylation of 2-pyridones employing α-diazocarbonyl derivatives (Scheme 1c).^7*c*^ However, there are still some limitations of diazo compounds serving as a carbene precursor, such as the potential explosiveness due to the evolution of nitrogen gas. To overcome these problems, other carbene surrogates were explored, such as cyclopropenes,^9^ hydrazones,^10^ ketone-functionalized enynes,^11^ triazoles,^12^ and sulfoxonium ylides. Sulfoxonium ylides have been reported to be employed in industry, and are more safe alternatives to diazo compounds.^13^ And recently, Barday and co-workers developed the cross-coupling reactions of α-carbonyl sulfoxonium ylides with arenes and heteroarenes using (Cp*RhCl_2_)_2_ as the catalyst (Scheme 1d).^13*d*^

Instead of using the noble metals such as rhodium and iridium, to date, more examples on direct C–H bond functionalization catalysed by ruthenium, a cost-effective transition-metal, has attracted attention and been developed. Herein, we reveal ruthenium(ii)-catalyzed C6-selective direct acylmethylation of pyridones using sulfoxonium ylides (Scheme 1e).

## 2 Results and discussion

Based on the precedent reported research, 2-pyridone (1a) and α-benzoyl sulfoxonium ylide (2a) were selected to probe the reaction conditions for transition-metal catalyzed acylmethylation of pyridone (Table 1). Initially, the coupling reaction between substrate 1a (0.4 mmol) and 2a (0.8 mmol) was triggered by a screen of various transition metal complexes. Ruthenium(ii) (5 mol%), cobalt(iii) (5 mol%), and rhodium(iii) (5 mol%) were independently investigated in the presence of AgSbF_6_ (10 mol%) in hexafluoroisopropanol (HFIP) and the mixture was stirred at 60 °C under an argon atmosphere for 24 h. The results indicated that [Ru(*p*-cymene)Cl_2_]_2_ was the optimal catalyst (Table 1, entries 1–3). Additionally, if replacing the [Ru(*p*-cymene)Cl_2_]_2_ with [RuCl(*p*-cymene)((*S*)-binap)]Cl, Ru(PPh_3_)_3_Cl_2_, or RuCl[(*R*,*R*)-Tsdpen](*p*-cymene), the yield of 3aa was decreased (Table 1, entries 4–6). Solvent was subsequently examined and results demonstrated that 3aa could be obtained in a higher yield in HFIP than in others including 1,2-dichloorethaan (DCE), acetonitrile, dioxane, methanol, and ethanol (Table 1, entries 7–11). Changing the additive from AgSbF_6_ to AgNTf_2_, AgOTf, or Ag(OAc)_2_ could diminish the yield of 3aa (Table 1, entries 12–14). The yield slightly decreased caused by the reduction of [Ru(*p*-cymene)Cl_2_]_2_ and AgSbF_6_ (Table 1, entries 15 and 16). Whilst when the reaction was conducted at 90 °C, 3aa could also be attained in 91% yield which was no more discrepancy with conducting at 60 °C (Table 1, entry 15). However, decreasing the temperature to 40 °C, the yield was reduced to 67% (Table 1, entry 16). The reaction could also be carried out in air with 76% yield (Table 1, entry 17), but without ruthenium(ii) complex or Ag(i) additive, the reaction was no longer proceeded (Table 1, entries 18 and 19).

**Table 1:** Optimization of the reaction conditions^a^

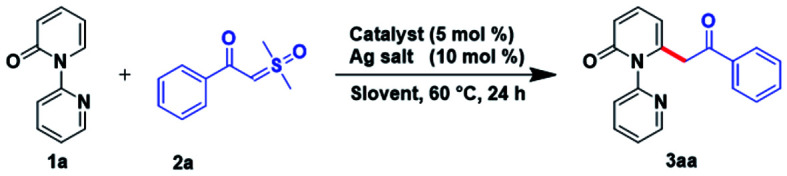				
Entry	Cat.^b^	Ag salt	Solvent	Yield^c^ (%)
1	A	AgSbF_6_	HFIP	13
2	B	AgSbF_6_	HFIP	49
**3**	**C**	**AgSbF**_**6**_	**HFIP**	**91**
4	D	AgSbF_6_	HFIP	31
5	E	AgSbF_6_	HFIP	16
6	F	AgSbF_6_	HFIP	63
7	C	AgSbF_6_	DCE	22
8	C	AgSbF_6_	MeCN	13
9	C	AgSbF_6_	Dioxane	21
10	C	AgSbF_6_	CH_3_OH	12
11	C	AgSbF_6_	CH_3_CH_2_OH	64
12	C	AgNTf_2_	HFIP	84
13	C	AgOTf	HFIP	78
14	C	Ag(OAc)_2_	HFIP	Trace
15^d^	C	AgSbF_6_	HFIP	84
16^e^	C	AgSbF_6_	HFIP	82
17^f^	C	AgSbF_6_	HFIP	91
18^g^	C	AgSbF_6_	HFIP	67
19^h^	C	AgSbF_6_	HFIP	76
20	—	AgSbF_6_	HFIP	N.R.
21	C	—	HFIP	N.R.

a Reaction conditions: compound 1a (0.4 mmol), compound 2a (0.8 mmol), cat. (5 mol%) and Ag salt (10 mol%) in solvent (3 mL) at 60 °C for 24 h, under Ar atmosphere. N.R. = no reaction.

b Catalyst A = [Cp*Co(CO)I_2_], catalyst B = (Cp*RhCl_2_)_2_, catalyst C = [Ru(*p*-cymene)Cl_2_]_2_, catalyst D = [RuCl(*p*-cymene)((*S*)-binap)]Cl, catalyst E = Ru(PPh_3_)_3_Cl_2_, catalyst F = RuCl[(*R*,*R*)-Tsdpen](*p*-cymene).

c Isolated yield.

d Cat. (2.5 mol%).

e Ag salt (5 mol%).

f At 90 °C.

g At 40 °C.

h At air condition.

**Scheme 2:**
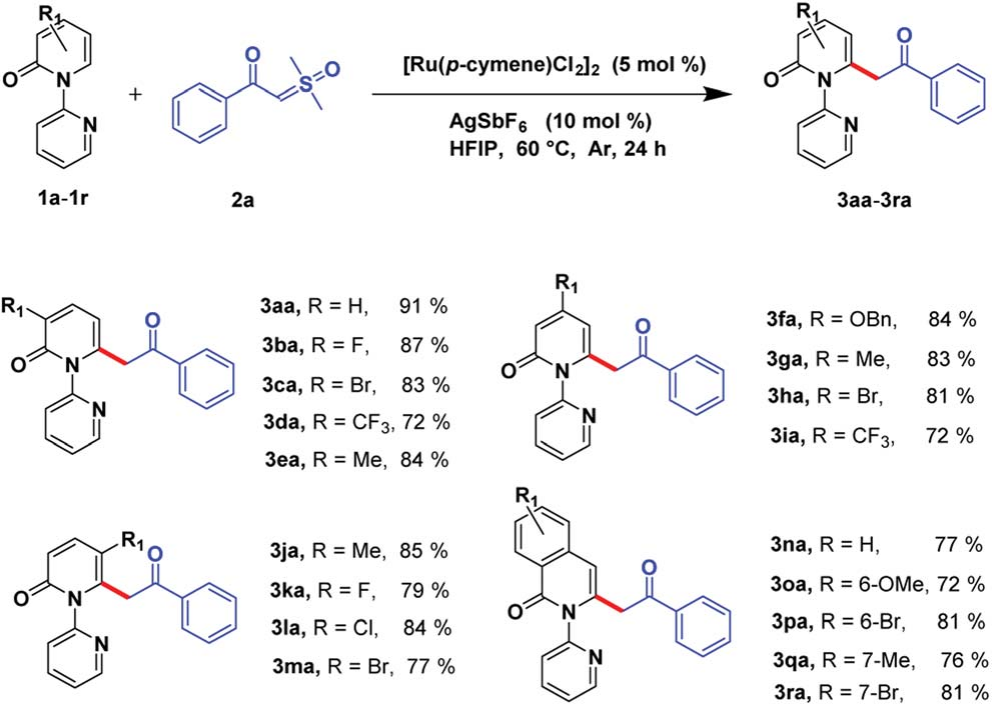
Substrate scope of pyridions.^*a*,*b a*^ Reaction conditions: compound 1a–1r (0.4 mmol), compound 2a (0.8 mmol), [Ru(*p*-cymene)Cl_2_]_2_ (5 mol%), and AgSbF_6_ (10 mol%) in HFIP (3 mL) at 60 °C, under Ar in 24 h. ^*b*^ Isolated yield.

With the optimized reaction conditions obtained, we investigated the substrate scope of pyridones 1a–1r (Scheme 2). The results showed that C3 substituted of 2-pyridones can sustain multiple functional groups, including electron-withdrawing groups or electron-donating groups, and even halogens to afford the desirable products in good to moderate yields (3ba–3ea, 72–87%). Substituents installed on the C4 position of pyridones can be processed smoothly by obtaining the desired products in good to moderate yields (3fa–3ia, 72–84%). Satisfyingly, although suffering from steric hindrance for the C5-substituted 2-pyridones, the desired compounds could be afforded in considerable yields (3ja–3ma, 77–85%). Moreover, this transformation was also compatible to isoquinolinones by attaining target molecules in good to excellent yields (3na–3ra, 72–81%).

**Scheme 3:**
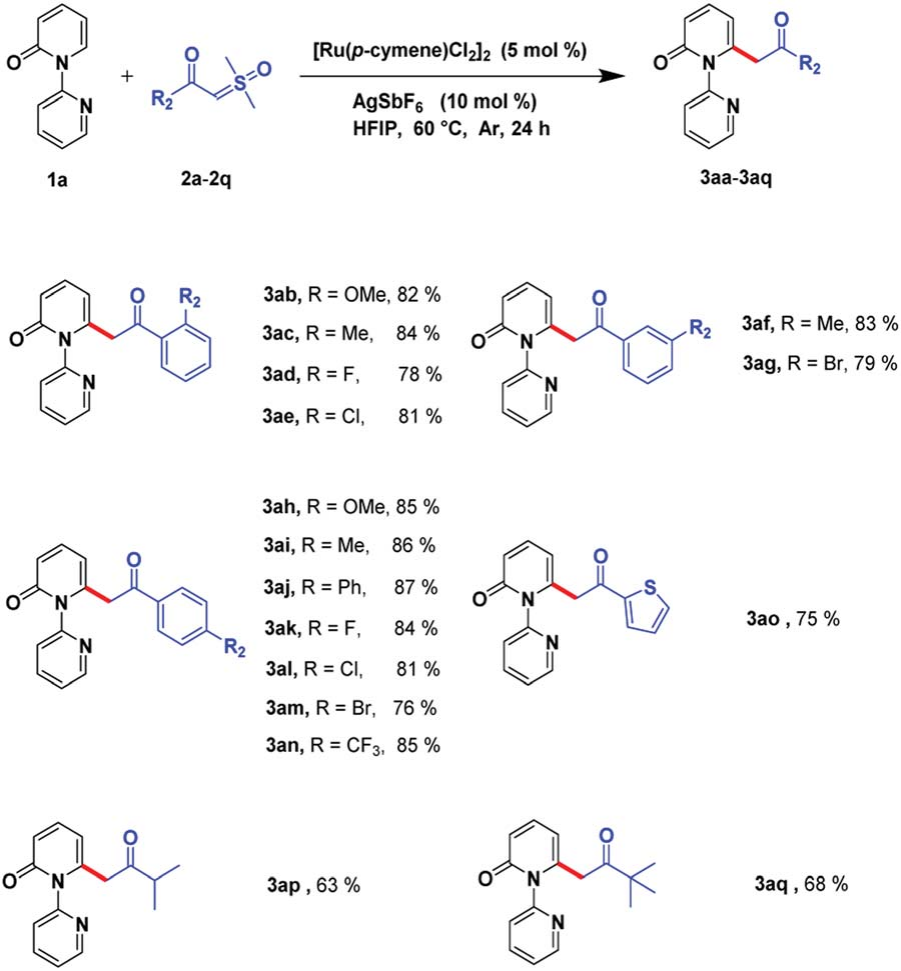
Substrate scope of sulfoxonium ylides.^*a*,*b a*^ Reaction conditions: compound 1a (0.4 mmol), compound 2a–2q (0.8 mmol), [Ru(*p*-cymene)Cl_2_]_2_ (5 mol%), and AgSbF_6_ (10 mol%) in HFIP (3 mL) at 60 °C, under Ar in 24 h. ^*b*^ Isolated yield.

Next, we investigated the scope of sulfoxonium ylides. The acylmethylation proposal was suitable for various kinds of α-benzoyl sulfoxonium ylides (Scheme 3). It can be tolerated by electron-donating groups, such as CH_3_ and OMe, and can be processed smoothly even if electron-withdrawing groups, such as CF_3_, or halogens (F, Cl, and Br), are incorporated in the derivatives. Different positions such as the *ortho*-, *meta*-, and *para*-of the phenyl ring can favorably afford the relevant products (3ab–3an) in high yields (76–86%). Gratifyingly, this reaction could also be carried out with heterocyclic compounds such as thiophene and the corresponding product (3ao) was detected in 75% yield. The sulfoxonium ylides can also bear some alkyl substrates and the relevant products could be detected in acceptable yields (3ap–3aq, 63–68%).

**Scheme 4:**
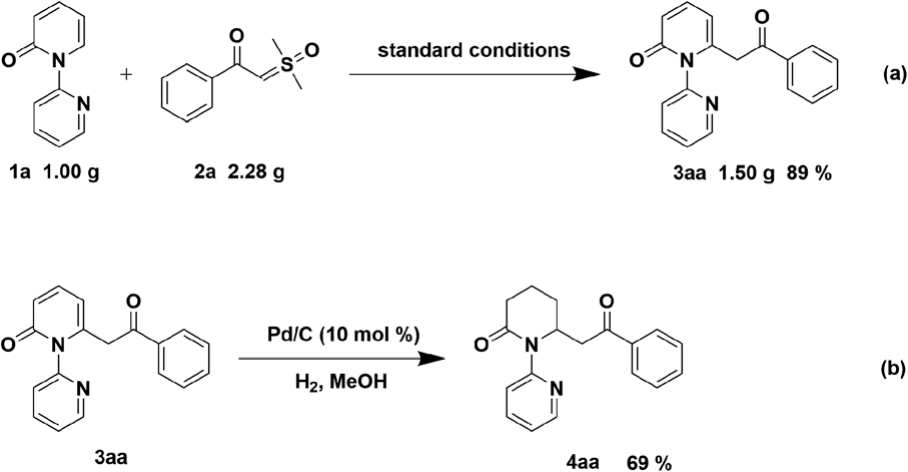
Gram-scale synthesis and synthetic transformation of compound 3aa.

To indicate the synthetic utility of this strategy for the approach to C6-acylmethylation piperidin-2-one, gram-scale synthesis of compound 3aa was conducted and the product was obtained in 89% yield (Scheme 4a). Furthermore, hydrogenation of 3aa was examined to form 4aa in 69% yield (Scheme 4b).

**Scheme 5:**
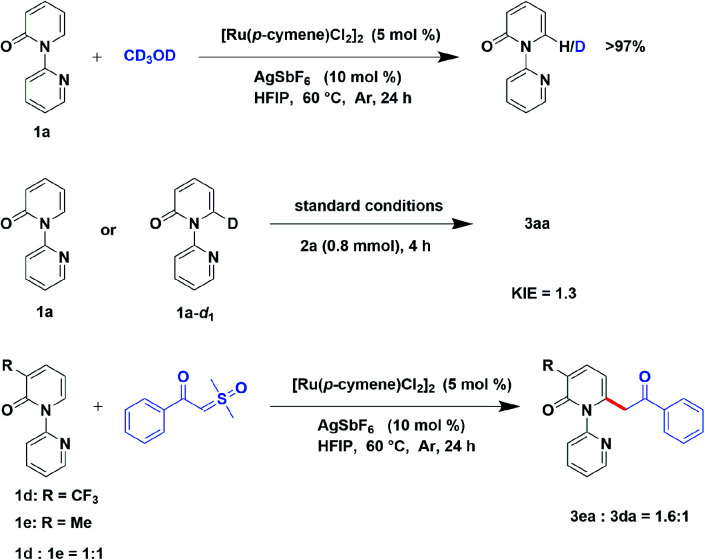
Mechanism study experiments.

In order to investigate the preliminary mechanism, a series of experiments were designed and performed. Firstly, a hydrogen–deuterium (H/D) exchange experiment was conducted to gain insight into the C–H cleavage, when 2-pyridone (1a) was examined in the optimized condition with the presence of CD_3_OD and no deuterium exchange was observed. It demonstrate the irreversible of C–H bond cleavage catalyzed by ruthenium. Furthermore, the kinetic isotope effect (KIE) experiment was conducted, employing [D_1_]-1a as substrate, illustrated a KIE of 1.3, indicated that the rate-limited step was not the division of the C–H bond. Additionally, an intermolecular competition reaction between 3-(trifluoromethyl)-2*H*-[1,2′-bipyridin]-2-one (1d) and 3-methyl-2*H*-[1,2′-bipyridin]-2-one (1e) with compound 2a were carried out in one sealed tube. Finally, it gave a higher yield of 3ea than 3da, revealing that the electron-donating substrate has faster reaction rate (Scheme 5).

**Scheme 6:**
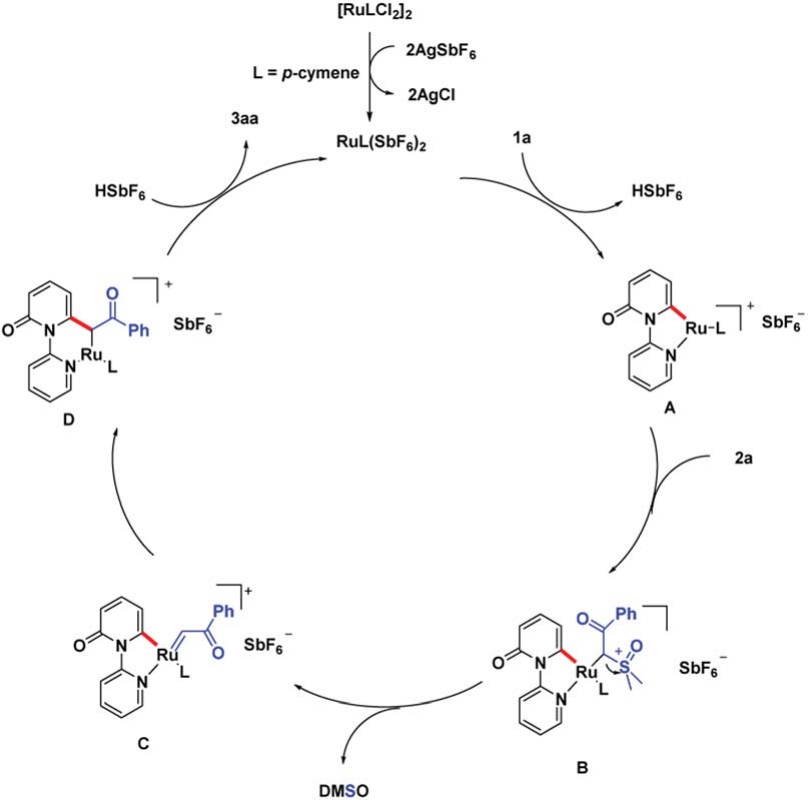
Proposed reaction mechanism.

On the basis of the preliminary experimental results, a plausible acylmethylation catalytic cycle is proposed (Scheme 6). The reactive Ru(ii) complex was first formed after ligand exchange of [Ru(*p*-cymene)Cl_2_]_2_ with AgSbF_6_, followed by a *ortho* C–H bond activation of pyridone. This process is assisted by the DG, pyridine motif and generate intermediate A. There is a ligand exchange among 2a and intermediate A, which affords the intermediate B. With the leaving of DMSO, ruthenium carbene intermediate C is produced. Migratory insertion of ruthenium-carbene generate intermediate D. Eventually, the intermediate D transfer the protonation, produce the product 3aa and liberate the active Ru-catalyst.

## 3 Conclusions

In summary, we achieved the ruthenium(ii)-catalyzed C6-selective C–H acylmethylation of pyridones employing sulfoxonium ylides. This new transformation is achieved using the excellent role of the Ru(ii) catalyst ([Ru(*p*-cymene)Cl_2_]_2_), and allows the synthesis of various C6-acylmethylated 2-pyridone derivatives. Besides, this approach features mild reaction conditions, moderate to excellent yields and high step economy. Furthermore, mechanistic study experiments were conducted to reveal the catalytic transformation cycle. It offers a direct and efficient way to synthesize pyridone derivatives and will be important to medicinal chemists.

## Conflicts of interest

There are no conflicts to declare.

## Supplementary Material

RA-010-C9RA10749E-s001
