# Supplementary material for: Ru(ii)-catalyzed C6-selective C–H acylmethylation of pyridones using sulfoxonium ylides as carbene precursors
Source: RSC Adv. 2020 Feb 11;10(11):6351–5. doi: 10.1039/c9ra10749e (PMC9049633; doi:10.1039/c9ra10749e)
Supplement: RA-010-C9RA10749E-s001 [file RA-010-C9RA10749E-s001.pdf]

Electronic Supplementary Material (ESI) for RSC Advances.
This journal is © The Royal Society of Chemistry 2020

Ru(II)-catalyzed C6-selective C-H Acylmethylation of Pyridone Using Sulfoxonium Ylides as Carbene Precursors

Yangjie Fu,[a,b] Zhaohui Wang,[a,b] Qiyu Zhang,[a,b] Zhiyu Li, [a] Hong Liu,[a,b] Xiaoling Bi,[*, a] and Jiang Wang*[,b]

[a]Jiangsu Key Laboratory of Drug Design and Optimization, China Pharmaceutical University, 24 Tongjiaxiang, Nanjing 210009, China

[b]State Key Laboratory of Drug Research and Key Laboratory of Receptor Research, Shanghai Institute of Materia Medica, Chinese Academy of Sciences, 555 Zu Chong Zhi Road, Shanghai 201203, China.

Contents

(A) General Information ········ S3

(B) General Procedures for the Substrates ········ S4

(C) General Procedures for the Products ········ S6

(D) Characterization Data ········ S7

(E) Gram-scale Synthesis of Compound 3aa ········ S24

(F) Synthetic Transformations of 3aa ········ S25

(G) Mechanistic Studies ········ S26

(H) Copies of ^{1}H NMR, ^{13}C NMR, and ^{19}F NMR Spectra for the Products ······ S29

(A) General information

Unless otherwise specified, the reagents were purchased from commercial sources, and used without further purification. All reactions were carried out in 1,1,1,3,3,3-Hexafluoro-2-propanol (HFIP). All products were characterized by their NMR and HRMS spectra. ^{1}H NMR, ^{13}C NMR, and ^{19}F NMR spectra were recorded on a 500, or 600 MHz instrument. The chemical shifts were reported in parts per million (ppm, δ) downfield from tetramethylsilane (TMS). Proton coupling patterns were described as singlet (s), doublet (d), triplet (t), quartet (q), multiplet (m), doublet of doublets (dd), triplet of doublets(td), doublet of triplets(dt) and broad (br). Highresolution mass spectra (HRMS) were measured on a Micromass Ultra Q-TOF spectrometer. Analytical thin-layer chromatography (TLC) was performed on HSGF 254 (0.2-0.3 mm thickness). Column chromatography was performed on silica gel (300-400 mesh) using ethyl acetate/methanol.

(B) General Procedures for the Substrates

General procedure for the preparation of pyridones **1a-1r**

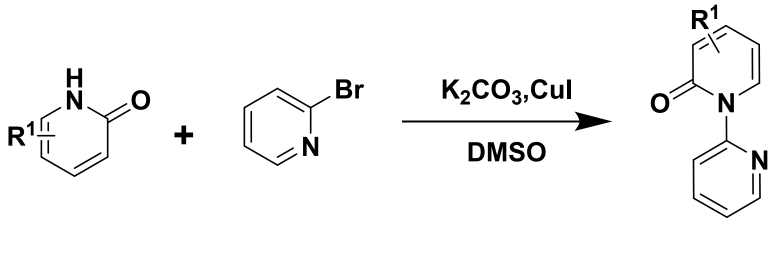

1a-1r

To a solution of pyridone (1.0 equiv.) and 2-bromopyridine (2.0 equiv.) in DMSO was added K_2CO_3 (1.2 equiv.) and CuI (0.1 equiv.) at room temperature. The resulting mixture was heated to 150 °C for 12 h. After cooled to room temperature water (15 mL) and ethyl acetate (20 mL) were added to the resulting slurry. The layers were separated and the aqueous layer was washed with ethyl acetate (2 x 30 mL) and the organic layers were combined. The organic solution was washed by brine and dried over anhydrous sodium sulphate (Na_2SO_4), filtered over a sintered funnel and evaporated to dryness. The crude product was purified by flash chromatography over silica gel using hexane/EtOAc (5:1) to afford the corresponding pyridones **1a-1r**.

General procedure for the preparation of sulfoxonium ylides **2a-2q**

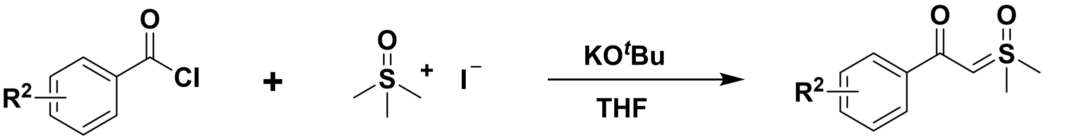

2a-2q

To a stirred solution of potassiumtert-butoxide (3.0 g, 27.2 mmol) in THF (30 mL) was added trimethylsulfoxonium iodide (5.0 g, 20.6 mmol) at room temperature. The resulting mixture is refluxed for 2 h. Then reaction mixture is cooled to 0 °C, followed by addition of acyl chlorides (7 mmol) in THF (5 mL). The reaction was allowed to room temperature and stirred for 3 h. Next, the solvent was evaporated, water (15 mL) and ethylacetate (20 mL) were added to the resulting slurry. The layers were separated and the aqueous layer was washed with ethyl acetate (2 x 30 mL) and the organic layers were combined. The organic solution was dried over anhydrous

sodium sulfate (Na_2SO_4), filtered over a sintered funnel and evaporated to dryness. The crude product was purified by flash chromatography over silica gel using EtOAc/MeOH (20:1) to afford the corresponding sulfoxonium ylides **2a-2q.**

(C) General procedures for the products 3aa-3ra, 3ab-3aq (compound 3aa as the example)

A tube was charged with [Ru(*p*-cymene)Cl_2]$_2$ (12.2 mg, 5 mol %), $AgSbF_6$ (15.6 mg, 10 mol %), sulfoxonium ylide (**2a**, 0.8 mmol), pyridone (**1a**, 0.4 mmol) and HFIP (3 mL). The reaction mixture was stirred at 60 °C for 24 h under air condition. After that, the solvent was filtered through a celite pad. The filtrate was extracted with EtOAc. The organic layer was washed with brine, dried over anhydrous Na_2SO_4, and concentrated in vacuo. The residue was purified by silica gel chromatography using EtOAc /MeOH (40:1) to afford the product **3aa** as a light yellow solid (yield: 91 %).

(D) Characterization Data

6-(2-oxo-2-phenylethyl)-2*H*-[1,2'-bipyridin]-2-one (3aa)

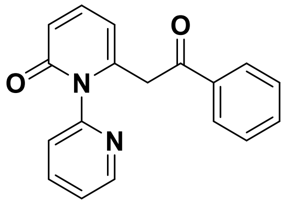

Light yellow solid (106 mg, yield: 91 %), m.p.:164-167 °C; ^{1}H NMR (500 MHz, DMSO-d_6) δ 8.41 (dd, J = 5.1, 1.9 Hz, 1H), 7.83 (td, J = 7.7, 1.9 Hz, 1H), 7.70 – 7.64 (m, 2H), 7.62 – 7.56 (m, 1H), 7.54 (dd, J = 9.3, 6.8 Hz, 1H), 7.43 (t, J = 7.8 Hz, 2H), 7.31 (dd, J = 7.6, 4.9, 1.1 Hz, 1H), 7.26 (d, J = 7.9 Hz, 1H), 6.47 (dd, J = 9.3, 1.2 Hz, 1H), 6.36 (dd, J = 6.8, 1.2 Hz, 1H), 4.23 (s, 2H). ^{13}C NMR (125 MHz, DMSO-d_6) δ 195.4, 162.9, 151.7, 149.6, 144.3, 141.0, 138.9, 136.1, 134.0, 129.1, 128.2, 125.3, 124.5, 119.3, 108.8, 43.3. HRMS (ESI) calculated for $C_{18}H_{14}N_2O_2$ [M+H]$^+$: 290.1055; found: 290.1048.

3-fluoro-6-(2-oxo-2-phenylethyl)-2*H*-[1,2'-bipyridin]-2-one (3ba)

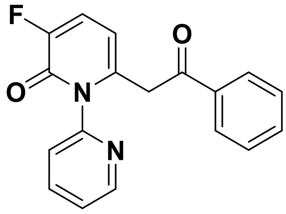

Light yellow solid (107 mg, yield: 87 %), m.p.:210-213 °C; ^{1}H NMR (600 MHz, DMSO-d_6) δ 8.42 (dd, J = 4.8, 1.1 Hz, 1H), 7.87 (td, J = 7.7, 1.9 Hz, 1H), 7.66 (dd, J = 8.3, 1.4 Hz, 2H), 7.60 – 7.56 (m, 1H), 7.55 (dd, J = 10.3, 7.7 Hz, 1H), 7.45 – 7.40 (m, 2H), 7.36 – 7.31 (m, 2H), 6.35 (dd, J = 7.7, 4.5 Hz, 1H), 4.23 (s, 2H). ^{13}C NMR (125 MHz, DMSO-d_6) δ 195.32, 156.55 (d, J = 26.5 Hz), 151.03 (d, J = 245.0 Hz), 150.8, 149.8, 139.7 (d, J = 5.2 Hz), 139.2, 136.0, 134.0, 129.1, 128.2, 125.2, 125.0, 121.7 (d, J = 16.2 Hz), 106.7 (d, J = 5.7 Hz), 42.9. ^{19}F NMR (470 MHz, $CDCl_3$) δ -131.46. HRMS (ESI) calculated for $C_{18}H_{13}FN_2O_2$ [M+H]$^+$: 308.0961; found: 308.0967.

3-bromo-6-(2-oxo-2-phenylethyl)-2*H*-[1,2'-bipyridin]-2-one (3ca)

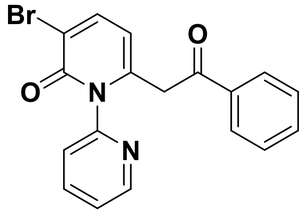

Light yellow solid (122 mg, yield: 83 %), m.p.:186-188 °C; ^{1}H NMR (500 MHz, DMSO-d_6) δ 8.42 (dd, J = 4.9, 1.9, 0.8 Hz, 1H), 8.07 (d, J = 7.5 Hz, 1H), 7.87 (td, J = 7.7, 1.9 Hz, 1H), 7.67 (dd, J = 8.3, 1.3 Hz, 2H), 7.59 (td, J = 7.3, 1.3 Hz, 1H), 7.46 – 7.41 (m, 2H), 7.35 (dd, J = 7.5, 4.8, 1.1 Hz, 1H), 7.32 (dd, J = 8.0, 1.0 Hz, 1H), 6.37 (d, J = 7.5 Hz, 1H), 4.23 (s, 2H). ^{13}C NMR (125 MHz, DMSO-d_6) δ 196.4, 160.6, 152.9, 151.1, 145.8, 144.4, 140.7, 137.4, 135.5, 130.6, 129.6, 126.4, 115.5, 110.7, 44.6. HRMS (ESI) calculated for $C_{18}H_{13}BrN_2O_2$ [M+H]$^+$: 368.0160; found: 368.0168.

6-(2-oxo-2-phenylethyl)-3-(trifluoromethyl)-2*H*-[1,2'-bipyridin]-2-one (3da)

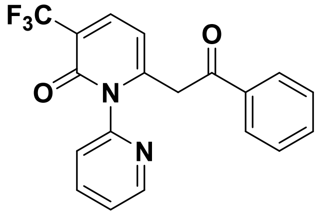

Light yellow solid (103 mg, yield: 72 %), m.p.:185-187 °C; ^{1}H NMR (500 MHz, DMSO-d_6) δ 8.44 (dd, J = 5.1, 1.8 Hz, 1H), 8.11 (d, J = 7.4 Hz, 1H), 7.89 (td, J = 7.7, 1.9 Hz, 1H), 7.71 – 7.67 (m, 2H), 7.63 – 7.58 (m, 1H), 7.44 (t, J = 7.8 Hz, 2H), 7.40 – 7.35 (m, 2H), 6.57 (d, J = 7.4 Hz, 1H), 4.34 (s, 2H). ^{13}C NMR (125 MHz, DMSO-d_6) δ 194.7, 158.9, 150.5, 150.0, 149.9, 140.9, 139.4, 135.8, 134.2, 129.2, 128.3, 125.2, 125.1, 124.2 (q, J = 305 Hz), 117.5 (q, J = 30.6 Hz), 107.7, 43.7. ^{19}F NMR (470 MHz, $CDCl_3$) δ -65.97. HRMS (ESI) calculated for $C_{19}H_{13}F_3N_2O_2$ [M+H]$^+$: 358.0929; found: 358.0924.

3-methyl-6-(2-oxo-2-phenylethyl)-2H-[1,2'-bipyridin]-2-one(3ea)

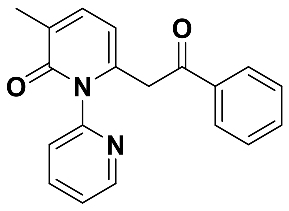

Light yellow solid (102 mg, yield: 84 %), m.p.:133-136 °C; ^{1}H NMR (500 MHz, DMSO-d_6) δ 8.38 (dd, J = 4.8, 1.8, 0.9 Hz, 1H), 7.80 (td, J = 7.7, 1.9 Hz, 1H), 7.63 (dd, J = 8.4, 1.3 Hz, 2H), 7.58 – 7.52 (m, 1H), 7.43 – 7.35 (m, 3H), 7.28 (dd, J = 7.5, 4.8, 1.1 Hz, 1H), 7.24 – 7.19 (m, 1H), 6.25 (d, J = 6.9 Hz, 1H), 4.15 (s, 2H), 2.01 (s, J = 1.1 Hz, 3H).^{13}C NMR (125 MHz, DMSO-d_6) δ 195.1, 162.9, 151.6, 149.0, 140.8, 138.4, 137.3, 135.7, 133.5, 128.6, 127.7, 127.2, 124.9, 124.0, 107.9, 42.7, 16.7. HRMS (ESI) calculated for $C_{19}H_{16}N_2O_2$ $[M+H]^+$: 304.1212; found: 304.1218.

4-(benzyloxy)-6-(2-oxo-2-phenylethyl)-2*H*-[1,2'-bipyridin]-2-one (3fa)

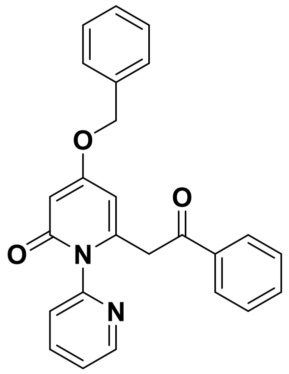

Light yellow solid (133 mg, yield: 84 %), m.p.:142-144 °C; ^{1}H NMR (600 MHz, DMSO-d_6) δ 8.37 (d, J = 3.0 Hz, 1H), 7.80 (t, J = 6.8 Hz, 1H), 7.65 (d, J = 7.7 Hz, 2H), 7.57 (t, J = 7.4 Hz, 1H), 7.48 (d, J = 7.4 Hz, 2H), 7.43 (dt, J = 11.4, 7.5 Hz, 4H), 7.38 (t, J = 7.3 Hz, 1H), 7.27 (dd, J = 7.5, 4.8 Hz, 1H), 7.21 (d, J = 7.9 Hz, 1H), 6.18 (d, J = 2.7 Hz, 1H), 5.98 (d, J = 2.7 Hz, 1H), 5.16 (s, 2H), 4.20 (s, 2H). ^{13}C NMR (125 MHz, DMSO-d_6) δ 195.2, 167.1, 164.3, 151.5, 149.4, 144.2, 138.7, 136.4, 136.0, 134.0, 129.1, 129.1, 128.7, 128.5, 128.2, 125.7, 124.3, 103.6, 96.9, 70.2, 43.2. HRMS (ESI) calculated for $C_{25}H_{20}N_2O_3$ $[M+H]^+$: 396.1474; found: 396.1480.

4-methyl-6-(2-oxo-2-phenylethyl)-2*H*-[1,2'-bipyridin]-2-one (3ga)

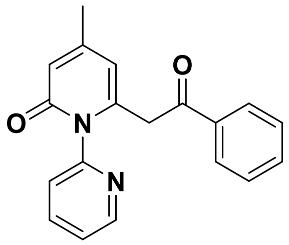

Light yellow solid (101 mg, yield: 83 %), m.p.:136-138 °C; ^{1}H NMR (500 MHz, DMSO-d_6) δ 8.39 (dd, J = 5.0, 1.9 Hz, 1H), 7.81 (td, J = 7.7, 1.9 Hz, 1H), 7.68 – 7.63 (m, 2H), 7.58 (t, J = 7.5 Hz, 1H), 7.43 (t, J = 7.7 Hz, 2H), 7.28 (dd, J = 7.3, 5.1 Hz, 1H), 7.22 (d, J = 8.0 Hz, 1H), 6.29 (s, 1H), 6.22 (d, J = 1.8 Hz, 1H), 4.19 (s, 2H), 2.21 (s, 3H). ^{13}C NMR (125 MHz, DMSO-d_6) δ 195.4, 162.8, 151.9, 151.7, 149.5, 142.9, 138.8, 136.1, 134.0, 129.1, 128.2, 125.5, 124.4, 117.5, 111.2, 43.2, 21.4. HRMS (ESI) calculated for $C_{19}H_{16}N_2O_2$ $[M+H]^+$: 304.1212; found: 304.1210.

4-bromo-6-(2-oxo-2-phenylethyl)-2*H*-[1,2'-bipyridin]-2-one (3ha)

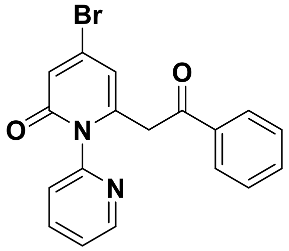

Light yellow solid (119 mg, yield: 81 %), m.p.:182-184 °C; ^{1}H NMR (500 MHz, DMSO-d_6) δ 8.40 (dd, J = 4.9, 2.0, 0.8 Hz, 1H), 7.85 (td, J = 7.7, 1.9 Hz, 1H), 7.69 – 7.64 (m, 2H), 7.62 – 7.57 (m, 1H), 7.46 – 7.41 (m, 2H), 7.33 (dd, J = 7.6, 4.9, 1.1 Hz, 1H), 7.29 (d, J = 7.9 Hz, 1H), 6.86 (d, J = 2.1 Hz, 1H), 6.69 (d, J = 2.1 Hz, 1H), 4.25 (s, 2H). ^{13}C NMR (125 MHz, DMSO-d_6) δ 196.3, 163.1, 152.3, 151.1, 146.7, 140.6, 137.8, 137.3, 135.6, 130.6, 129.6, 126.6, 126.3, 122.4, 114.0, 44.4. HRMS (ESI) calculated for $C_{18}H_{13}BrN_2O_2$ $[M+H]^+$: 368.0160; found: 368.0166.

6-(2-oxo-2-phenylethyl)-4-(trifluoromethyl)-2*H*-[1,2'-bipyridin]-2-one (3ia)

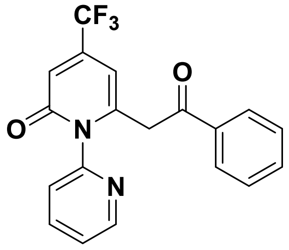

Light yellow solid (101 mg, yield: 72 %), m.p.:145-148 ºC; ^{1}H NMR (600 MHz, DMSO-d_6) δ 8.41 (dd, J = 5.1, 1.8 Hz, 1H), 7.88 (td, J = 7.7, 1.9 Hz, 1H), 7.66 (d, J = 6.9 Hz, 2H), 7.59 (t, J = 7.4 Hz, 1H), 7.43 (t, J = 7.8 Hz, 2H), 7.37 – 7.31 (m, 2H), 6.93 (s, 1H), 6.71 (d, J = 2.0 Hz, 1H), 4.35 (s, 2H). ^{13}C NMR (125 MHz, DMSO-d_6) δ 194.8, 161.9, 150.8, 149.8, 147.5, 140.5 (q, J = 33.0 Hz), 139.3, 135.8, 134.2, 129.2, 128.2, 125.1, 125.0, 122.8 (q, J = 274.3 Hz), 117.0 (d, J = 4.2 Hz), 103.9 – 102.5 (m), 43.5. ^{19}F NMR (470 MHz, DMSO-d_6) δ -65.53. HRMS (ESI) calculated for $C_{19}H_{13}F_3N_2O_2$ $[M+H]^+$: 358.0929; found: 358.0923.

5-methyl-6-(2-oxo-2-phenylethyl)-2*H*-[1,2'-bipyridin]-2-one (3ja)

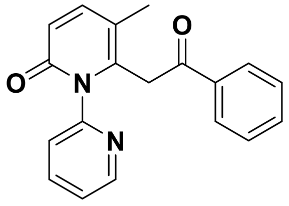

Light yellow solid (103 mg, yield: 85 %), m.p.:206-209 ºC; ^{1}H NMR (600 MHz, DMSO-d_6) δ 8.36 (dd, J = 5.3, 1.7 Hz, 1H), 7.83 (td, J = 7.7, 1.9 Hz, 1H), 7.67 (dd, J = 8.3, 1.4 Hz, 2H), 7.62 – 7.57 (m, 1H), 7.48 (d, J = 9.4 Hz, 1H), 7.46 – 7.40 (m, 2H), 7.31 (dd, J = 7.5, 4.9, 1.1 Hz, 1H), 7.20 (d, J = 7.9 Hz, 1H), 6.43 (d, J = 9.4 Hz, 1H), 4.11 (s, 2H), 2.04 (s, 3H). ^{13}C NMR (125 MHz, DMSO-d_6) δ 194.7, 162.2, 152.4, 149.8, 144.5, 140.6, 139.2, 136.2, 134.0, 129.1, 128.2, 125.0, 124.5, 119.0, 114.8, 39.5, 17.2. HRMS (ESI) calculated for $C_{19}H_{16}N_2O_2$ $[M+H]^+$: 304.1212; found: 304.1205.

5-fluoro-6-(2-oxo-2-phenylethyl)-2*H*-[1,2'-bipyridin]-2-one (3ka)

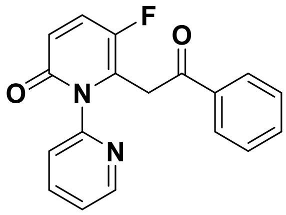

Light yellow solid (97 mg, yield: 79 %), m.p.:161-163 ^{o}C; ^{1}H NMR (500 MHz, DMSO-d_6) δ 8.37 (dd, J = 4.8, 1.9, 0.8 Hz, 1H), 7.88 (td, J = 7.7, 1.9 Hz, 1H), 7.78 (dd, J = 10.1, 8.1 Hz, 1H), 7.71 (dd, J = 8.4, 1.3 Hz, 2H), 7.63 – 7.59 (m, 1H), 7.47 – 7.42 (m, 2H), 7.35 (dd, J = 7.5, 4.9, 1.1 Hz, 1H), 7.32 (d, J = 8.0 Hz, 1H), 6.56 (dd, J = 10.1, 5.1 Hz, 1H), 4.20 (d, J = 3.0 Hz, 2H). ^{13}C NMR (125 MHz, DMSO-d_6) δ 194.0, 161.0, 150.9, 149.8, 147.2, 145.4, 139.4, 135.8, 134.2, 133.0, 132.7, 130.1, 129.9, 129.2, 128.3, 125.2, 124.9, 120.5, 120.4, 37.1. ^{19}F NMR (470 MHz, CDCl$_3$) δ -148.66. HRMS (ESI) calculated for $C_{18}H_{13}FN_2O_2$ [M+H]$^+$: 308.0961; found: 308.0967.

5-chloro-6-(2-oxo-2-phenylethyl)-2*H*-[1,2'-bipyridin]-2-one (3la)

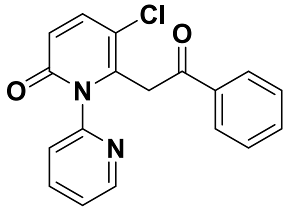

Light yellow solid (109 mg, yield: 84 %), m.p.:189-190 ^{o}C; ^{1}H NMR (600 MHz, DMSO-d_6) δ 8.41 (dd, J = 4.8, 1.8 Hz, 1H), 7.89 (td, J = 7.7, 1.9 Hz, 1H), 7.75 (d, J = 6.9 Hz, 2H), 7.71 (d, J = 9.9 Hz, 1H), 7.62 (t, J = 7.4 Hz, 1H), 7.45 (t, J = 7.8 Hz, 2H), 7.37 (dd, J = 6.8, 4.6 Hz, 1H), 7.30 (d, J = 7.9 Hz, 1H), 6.58 (d, J = 9.9 Hz, 1H), 4.25 (s, 2H). ^{13}C NMR (125 MHz, DMSO-d_6) δ 193.4, 161.5, 151.5, 150.0, 142.0, 141.4, 139.6, 135.7, 134.3, 129.2, 128.3, 125.1, 124.8, 120.8, 113.2, 41.6. HRMS (ESI) calculated for $C_{18}H_{13}ClN_2O_2$ [M+H]$^+$: 324.0666; found: 324.0672.

5-bromo-6-(2-oxo-2-phenylethyl)-2*H*-[1,2'-bipyridin]-2-one (3ma)

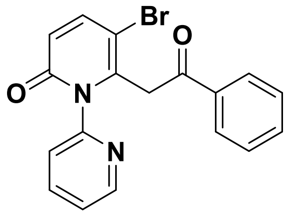

Light yellow solid (113 mg, yield: 77 %), m.p.:184-186 °C; ^{1}H NMR (600 MHz, DMSO-d_6) δ 8.42 (dd, J = 4.9, 1.9, 0.7 Hz, 1H), 7.89 (td, J = 7.7, 1.9 Hz, 1H), 7.78 (d, J = 9.9 Hz, 1H), 7.76 (dd, J = 8.4, 1.3 Hz, 2H), 7.64 – 7.60 (m, 1H), 7.47 – 7.43 (m, 2H), 7.37 (dd, J = 7.6, 4.9, 1.1 Hz, 1H), 7.29 (d, J = 7.9 Hz, 1H), 6.51 (d, J = 9.8 Hz, 1H), 4.27 (s, 2H). ^{13}C NMR (125 MHz, DMSO-d_6) δ 194.7, 163.1, 153.2, 151.4, 145.8, 144.1, 141.0, 137.1, 135.8, 130.7, 129.7, 126.5, 126.1, 122.4, 103.2, 45.5. HRMS (ESI) calculated for $C_{18}H_{13}BrN_2O_2$ [M+H]$^+$: 368.0160; found: 368.0168.

3-(2-oxo-2-phenylethyl)-2-(pyridin-2-yl)isoquinolin-1(2*H*)-one (3na)

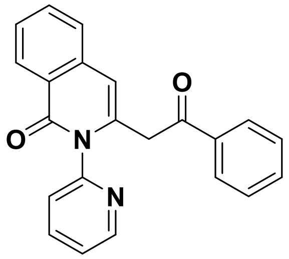

Light yellow solid (105 mg, yield: 77 %), m.p.:168-170 °C; ^{1}H NMR (600 MHz, DMSO-d_6) δ 8.42 (dd, J = 4.9, 1.1 Hz, 1H), 8.21 (d, J = 6.8 Hz, 1H), 7.84 (td, J = 7.7, 1.9 Hz, 1H), 7.81 – 7.77 (m, 1H), 7.70 (d, J = 6.9 Hz, 1H), 7.68 – 7.65 (m, 2H), 7.60 – 7.54 (m, 2H), 7.45 – 7.41 (m, 2H), 7.34 – 7.30 (m, 2H), 6.75 (s, 1H), 4.26 (s, 2H). ^{13}C NMR (125 MHz, DMSO-d_6) δ 195.8, 162.6, 151.9, 149.5, 138.9, 137.6, 137.1, 136.2, 133.9, 133.7, 129.1, 128.2, 127.7, 127.3, 126.5, 125.8, 125.1, 124.4, 108.5, 43.4. HRMS (ESI) calculated for $C_{22}H_{16}N_2O_2$ [M+H]$^+$: 340.1212; found: 340.1204.

6-methoxy-3-(2-oxo-2-phenylethyl)-2-(pyridin-2-yl)isoquinolin-1(2*H*)-one (3oa)

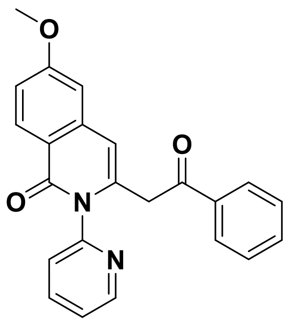

Light yellow solid (120 mg, yield: 81 %), m.p.:174-176 °C; ^{1}H NMR (500 MHz, DMSO-d_6) δ 8.42 (dd, *J* = 5.5, 1.8 Hz, 1H), 8.12 (d, *J* = 8.8 Hz, 1H), 7.83 (td, *J* = 7.7, 1.9 Hz, 1H), 7.70 – 7.66 (m, 2H), 7.59 (td, *J* = 7.3, 1.3 Hz, 1H), 7.44 (t, *J* = 7.8 Hz, 2H), 7.33 – 7.29 (m, 2H), 7.16 (d, *J* = 2.5 Hz, 1H), 7.13 (dd, *J* = 8.8, 2.5 Hz, 1H), 6.66 (s, 1H), 4.24 (s, 2H), 3.92 (s, 3H). ^{13}C NMR (125 MHz, DMSO-d_6) δ 195.8, 163.4, 162.2, 151.9, 149.5, 139.3, 138.8, 138.2, 136.2, 133.9, 129.8, 129.1, 128.2, 125.9, 124.3, 118.7, 116.6, 108.3, 107.6, 56.1, 43.5. HRMS (ESI) calculated for $C_{23}H_{18}N_2O_3$ $[M+H]^+$: 370.1317; found: 370.1311.

6-bromo-3-(2-oxo-2-phenylethyl)-2-(pyridin-2-yl)isoquinolin-1(2*H*)-one (3pa)

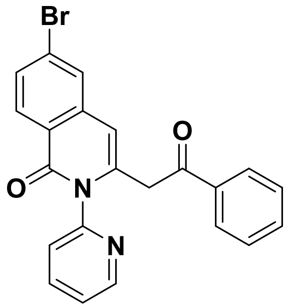

Light yellow solid (120 mg, yield: 72 %), m.p.:209-211 °C; ^{1}H NMR (500 MHz, DMSO-d_6) δ 8.42 (dd, *J* = 4.9, 1.8 Hz, 1H), 8.12 (d, *J* = 8.6 Hz, 1H), 8.01 (d, *J* = 2.0 Hz, 1H), 7.85 (td, *J* = 7.7, 1.9 Hz, 1H), 7.69 (dd, *J* = 16.4, 8.5, 1.7 Hz, 3H), 7.62 – 7.57 (m, 1H), 7.44 (t, *J* = 7.7 Hz, 2H), 7.37 – 7.30 (m, 2H), 6.74 (s, 1H), 4.27 (s, 2H). ^{13}C NMR (125 MHz, DMSO-d_6) δ 197.0, 163.6, 153.0, 151.0, 140.7, 140.4, 140.2, 137.6, 135.4, 131.7, 131.5, 130.5, 130.1, 129.6, 129.2, 127.2, 126.0, 125.3, 108.8, 44.9. HRMS (ESI) calculated for $C_{22}H_{15}BrN_2O_2$ $[M+H]^+$: 418.0317; found: 418.0323.

7-methyl-3-(2-oxo-2-phenylethyl)-2-(pyridin-2-yl)isoquinolin-1(2*H*)-one (3qa)

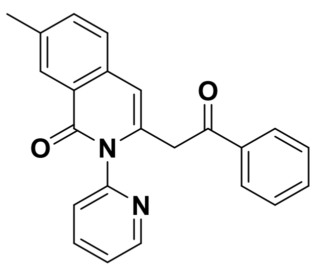

Light yellow solid (115 mg, yield: 81 %), m.p.:196-198 °C; ^{1}H NMR (500 MHz, DMSO-d_6) δ 8.43 (dd, J = 5.3, 1.8 Hz, 1H), 8.02 (s, 1H), 7.84 (td, J = 7.7, 2.0 Hz, 1H), 7.71 – 7.66 (m, 2H), 7.64 – 7.60 (m, 2H), 7.60 – 7.56 (m, 1H), 7.43 (t, J = 7.8 Hz, 2H), 7.31 (td, J = 7.3, 1.6 Hz, 2H), 6.71 (s, 1H), 4.25 (s, 2H), 2.48 (s, 3H). ^{13}C NMR (125 MHz, DMSO-d_6) δ 195.9, 162.6, 152.0, 149.5, 138.8, 136.9, 136.6, 136.2, 135.0, 134.9, 133.9, 129.1, 128.2, 127.2, 126.5, 125.9, 125.0, 124.3, 108.4, 43.3, 21.5. HRMS (ESI) calculated for $C_{23}H_{18}N_2O_2$ [M+H]$^+$: 354.1368; found: 354.1364.

7-bromo-3-(2-oxo-2-phenylethyl)-2-(pyridin-2-yl)isoquinolin-1(2*H*)-one (3ra)

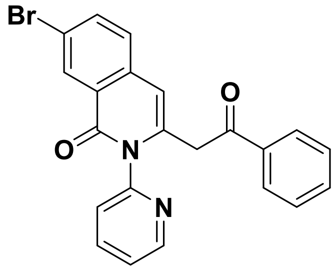

Light yellow solid (127 mg, yield: 76 %), m.p.:182-184 °C; ^{1}H NMR (600 MHz, DMSO-d_6) δ 8.42 (dd, J = 4.9, 1.9, 0.9 Hz, 1H), 8.29 (d, J = 2.2 Hz, 1H), 7.95 (dd, J = 8.5, 2.2 Hz, 1H), 7.85 (td, J = 7.7, 1.9 Hz, 1H), 7.70 (d, J = 8.5 Hz, 1H), 7.67 (dd, J = 8.4, 1.3 Hz, 2H), 7.61 – 7.56 (m, 1H), 7.45 – 7.40 (m, 2H), 7.35 – 7.31 (m, 2H), 6.79 (s, 1H), 4.27 (s, 2H). ^{13}C NMR (125 MHz, DMSO-d_6) δ 195.6, 161.5, 151.6, 149.6, 139.0, 138.5, 136.5, 136.1, 136.1, 134.0, 129.8, 129.1, 129.0, 128.2, 126.5, 125.7, 124.6, 120.0, 108.0, 43.4, 40.5, 40.4, 40.2, 40.0, 39.9, 39.7, 39.5. HRMS (ESI) calculated for $C_{22}H_{15}BrN_2O_2$ [M+H]$^+$: 418.0317; found: 418.0319.

6-(2-(2-methoxyphenyl)-2-oxoethyl)-2*H*-[1,2'-bipyridin]-2-one (3ab)

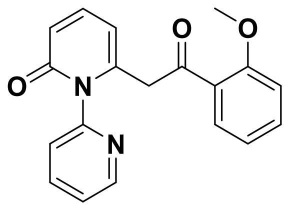

Yellow solid (105 mg, yield: 82 %), m.p.:109-112 ºC; ^{1}H NMR (600 MHz, DMSO-d_6) δ 8.45 (d, J = 3.0 Hz, 1H), 7.86 (td, J = 7.7, 1.9 Hz, 1H), 7.55 – 7.45 (m, 2H), 7.36 – 7.31 (m, 2H), 7.24 (d, J = 7.9 Hz, 1H), 7.05 (d, J = 8.4 Hz, 1H), 6.95 (t, J = 7.5 Hz, 1H), 6.44 (d, J = 9.3 Hz, 1H), 6.32 (d, J = 6.8 Hz, 1H), 4.11 (s, 2H), 3.73 (s, 3H). ^{13}C NMR (125 MHz, DMSO-d_6) δ 196.3, 162.9, 158.6, 151.8, 149.6, 144.4, 141.0, 138.9, 134.7, 130.3, 126.7, 125.2, 124.5, 120.8, 119.1, 112.8, 108.5, 56.2, 47.8. HRMS (ESI) calculated for $C_{19}H_{16}N_2O_3$[M+H]$^+$: 320.1161; found: 320.1169.

6-(2-oxo-2-(o-tolyl)ethyl)-2*H*-[1,2'-bipyridin]-2-one (3ac)

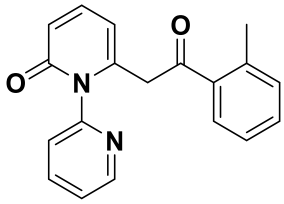

Light yellow solid (102 mg, yield: 84 %), m.p.:162-164 ºC; ^{1}H NMR (500 MHz, DMSO-d_6) δ 8.45 (dd, J = 5.0, 2.0, 0.9 Hz, 1H), 7.90 (td, J = 7.7, 1.9 Hz, 1H), 7.55 (dd, J = 9.3, 6.8 Hz, 1H), 7.41 – 7.35 (m, 2H), 7.31 (dd, J = 7.9, 1.2 Hz, 2H), 7.25 – 7.17 (m, 2H), 6.47 (dd, J = 9.3, 1.2 Hz, 1H), 6.37 (dd, J = 6.8, 1.2 Hz, 1H), 4.15 (s, 2H), 2.27 (s, 3H). ^{13}C NMR (125 MHz, DMSO-d_6) δ 198.2, 162.9, 151.8, 149.6, 144.1, 141.0, 139.0, 137.9, 136.7, 132.2, 132.2, 128.9, 126.2, 125.3, 124.6, 119.3, 109.0, 45.9, 21.0. HRMS (ESI) calculated for $C_{19}H_{16}N_2O_2$ [M+H]$^+$: 304.1212; found: 304.1206.

6-(2-(2-fluorophenyl)-2-oxoethyl)-2*H*-[1,2'-bipyridin]-2-one (3ad)

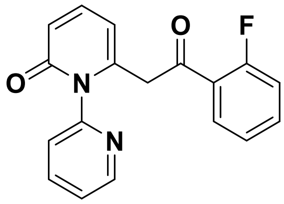

Yellow solid (96 mg, yield: 78 %), m.p.:110-112 ºC; ^{1}H NMR (500 MHz, DMSO-d_6)

δ 8.44 – 8.39 (m, 1H), 7.88 (td, *J* = 7.7, 1.9 Hz, 1H), 7.62 (dd, *J* = 8.6, 7.1, 5.1, 1.9 Hz, 1H), 7.55 (td, *J* = 9.2, 8.5, 6.4 Hz, 2H), 7.34 (dd, *J* = 7.5, 4.9, 1.1 Hz, 1H), 7.30 – 7.22 (m, 3H), 6.48 (dd, *J* = 9.3, 1.2 Hz, 1H), 6.37 (dd, *J* = 6.9, 1.2 Hz, 1H), 4.14 (s, 2H). ^{13}C NMR (125 MHz, DMSO-d_6) δ 193.23, 162.85, 161.03 (d, *J* = 253.9 Hz), 151.7, 149.6, 143.5, 141.0, 139.0, 136.0, 130.7, 125.3, 125.2, 124.8 (d, *J* = 12.2 Hz), 124.6, 119.5, 117.2 (d, *J* = 23.1 Hz), 108.9, 47.1. ^{19}F NMR (470 MHz, $CDCl_3$) δ -111.86. HRMS (ESI) calculated for $C_{18}H_{13}FN_2O_2$ $[M+H]^+$: 308.0961; found: 308.0965.

6-(2-(2-chlorophenyl)-2-oxoethyl)-2*H*-[1,2'-bipyridin]-2-one (3ae)

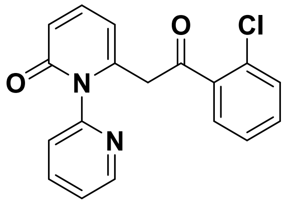

Yellow solid (105 mg, yield: 81 %), m.p.:137-140 °C; ^{1}H NMR (600 MHz, DMSO-d_6) δ 8.49 (d, *J* = 5.6 Hz, 1H), 7.93 (t, *J* = 6.9 Hz, 1H), 7.56 – 7.35 (m, 5H), 7.34 – 7.28 (m, 2H), 6.48 (d, *J* = 9.3 Hz, 1H), 6.39 (d, *J* = 6.8 Hz, 1H), 4.17 (s, 2H). ^{13}C NMR (125 MHz, DMSO-d_6) δ 196.6, 162.8, 151.5, 149.6, 142.9, 140.9, 139.2, 137.2, 133.3, 131.0, 130.2, 129.9, 127.7, 125.3, 124.7, 119.5, 108.9, 46.7. HRMS (ESI) calculated for $C_{18}H_{13}ClN_2O_2$ $[M+H]^+$: 324.0666; found: 324.0661.

6-(2-oxo-2-(m-tolyl)ethyl)-2*H*-[1,2'-bipyridin]-2-one (3af)

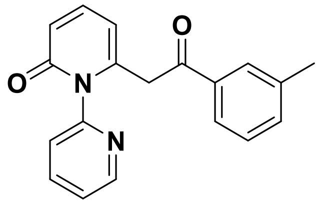

Yellow solid (101 mg, yield: 83 %), m.p.:113-116 °C; ^{1}H NMR (600 MHz, DMSO-d_6) δ 8.42 (dd, *J* = 4.9, 1.8 Hz, 1H), 7.83 (td, *J* = 7.7, 1.9 Hz, 1H), 7.53 (dd, *J* = 9.3, 6.8 Hz, 1H), 7.44 (d, *J* = 8.8 Hz, 2H), 7.39 (d, *J* = 7.5 Hz, 1H), 7.30 (q, *J* = 7.1 Hz, 2H), 7.24 (d, *J* = 7.9 Hz, 1H), 6.46 (d, *J* = 9.4 Hz, 1H), 6.34 (d, *J* = 5.6 Hz, 1H), 4.19 (s, 2H), 2.30 (s, 3H). ^{13}C NMR (125 MHz, DMSO-d_6) δ 195.4, 162.9, 151.7, 149.6, 144.3, 141.0, 138.9, 138.5, 136.1, 134.6, 129.0, 128.6, 125.4, 125.3, 124.5, 119.3,

108.7, 43.3, 21.2. HRMS (ESI) calculated for $C_{19}H_{16}N_2O_2$ $[M+H]^+$: 304.1212; found: 304.1205.

6-(2-(3-bromophenyl)-2-oxoethyl)-2*H*-[1,2'-bipyridin]-2-one (3ag)

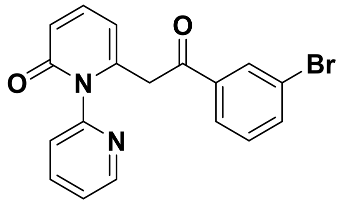

Yellow solid (116 mg, yield: 79 %), m.p.:160-164 °C; ^{1}H NMR (600 MHz, DMSO-d_6) δ 8.39 (dd, J = 5.0, 1.8 Hz, 1H), 7.85 (td, J = 7.7, 1.9 Hz, 1H), 7.79 (d, J = 6.7 Hz, 2H), 7.65 (dd, J = 7.9, 1.3 Hz, 1H), 7.53 (dd, J = 9.3, 6.8 Hz, 1H), 7.39 (t, J = 8.2 Hz, 1H), 7.32 (dd, J = 6.5, 4.8 Hz, 1H), 7.26 (d, J = 8.0 Hz, 1H), 6.47 (d, J = 8.1 Hz, 1H), 6.35 (d, J = 6.8 Hz, 1H), 4.22 (s, 2H). ^{13}C NMR (125 MHz, DMSO-d_6) δ 194.4, 162.9, 151.7, 149.6, 143.9, 141.0, 139.0, 138.1, 136.6, 131.4, 130.7, 127.3, 125.3, 124.6, 122.5, 119.4, 108.9, 43.4. HRMS (ESI) calculated for $C_{18}H_{13}BrN_2O_2$ $[M+H]^+$: 368.0160; found: 368.0169.

6-(2-(4-methoxyphenyl)-2-oxoethyl)-2*H*-[1,2'-bipyridin]-2-one (3ah)

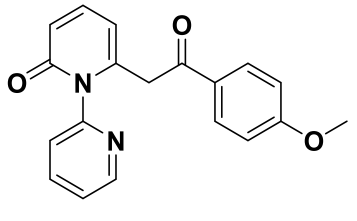

Light yellow solid (110 mg, yield: 85 %), m.p.:133-135 °C; ^{1}H NMR (500 MHz, DMSO- d_6) δ 8.43 (dd, J = 4.8, 1.9, 0.9 Hz, 1H), 7.82 (td, J = 7.7, 1.9 Hz, 1H), 7.67 – 7.63 (m, 2H), 7.53 (dd, J = 9.3, 6.8 Hz, 1H), 7.32 (dd, J = 7.5, 4.9, 1.1 Hz, 1H), 7.24 (dt, J = 8.0, 1.0 Hz, 1H), 6.95 – 6.91 (m, 2H), 6.46 (dd, J = 9.3, 1.2 Hz, 1H), 6.33 (dd, J = 6.8, 1.3 Hz, 1H), 4.15 (s, 2H), 3.81 (s, 3H). ^{13}C NMR (125 MHz, DMSO-d_6) δ 193.2, 163.3, 162.5, 151.2, 149.1, 144.2, 140.5, 138.4, 130.1, 128.5, 124.9, 124.1, 118.7, 113.8, 108.2, 55.6, 42.5. HRMS (ESI) calculated for $C_{19}H_{16}N_2O_3$ $[M+H]^+$: 320.1161; found: 320.1167.

6-(2-oxo-2-(p-tolyl)ethyl)-2*H*-[1,2'-bipyridin]-2-one (3ai)

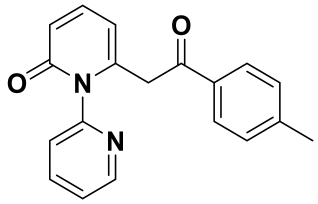

Light yellow solid (103 mg, yield: 86 %), m.p.:186-188 °C; ^{1}H NMR (500 MHz, DMSO-d_6) δ 8.42 (dd, J = 4.9, 1.9 Hz, 1H), 7.83 (td, J = 7.7, 1.9 Hz, 1H), 7.56 (d, J = 8.0 Hz, 2H), 7.53 (dd, J = 9.3, 6.7 Hz, 1H), 7.31 (dd, J = 7.5, 4.9 Hz, 1H), 7.23 (t, J = 7.6 Hz, 3H), 6.46 (d, J = 8.1 Hz, 1H), 6.34 (d, J = 5.8 Hz, 1H), 4.18 (s, 2H), 2.33 (s, 3H). ^{13}C NMR (125 MHz, DMSO-d_6) δ 194.4, 162.5, 151.2, 149.1, 144.0, 143.9, 140.5, 138.4, 133.1, 129.2, 127.9, 124.9, 124.1, 118.8, 108.3, 42.8, 21.1. HRMS (ESI) calculated for $C_{19}H_{16}N_2O_2$ $[M+H]^+$: 304.1212; found: 304.1220.

6-(2-([1,1'-biphenyl]-4-yl)-2-oxoethyl)-2*H*-[1,2'-bipyridin]-2-one (3aj)

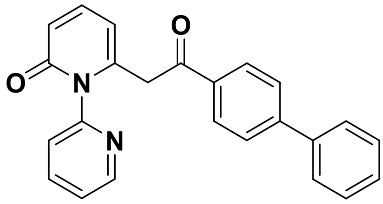

Light yellow solid (127 mg, yield: 87 %), m.p.:135-138 °C; ^{1}H NMR (600 MHz, DMSO-d_6) δ 8.43 (d, J = 4.9 Hz, 1H), 7.85 (t, J = 6.9 Hz, 1H), 7.73 (dd, J = 15.3, 6.3 Hz, 6H), 7.54 (dd, J = 9.3, 6.8 Hz, 1H), 7.49 (t, J = 7.5 Hz, 2H), 7.43 (t, J = 7.3 Hz, 1H), 7.35 – 7.30 (m, 1H), 7.28 (d, J = 8.0 Hz, 1H), 6.48 (d, J = 9.3 Hz, 1H), 6.37 (d, J = 6.8 Hz, 1H), 4.25 (s, 2H). ^{13}C NMR (125 MHz, DMSO-d_6) δ 194.4, 162.5, 151.3, 149.2, 144.8, 143.8, 140.5, 138.7, 138.5, 134.4, 129.1, 128.7, 128.5, 127.0, 126.8, 124.9, 124.1, 118.8, 108.3, 42.9. HRMS (ESI) calculated for $C_{24}H_{18}N_2O_2$ $[M+H]^+$: 336.1368; found: 336.1374

6-(2-(4-fluorophenyl)-2-oxoethyl)-2*H*-[1,2'-bipyridin]-2-one (3ak)

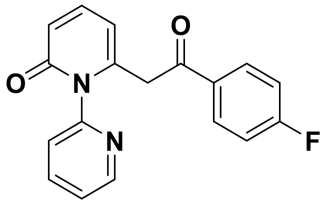

Light yellow solid (104 mg, yield: 84 %), m.p.:190-192 °C; ^{1}H NMR (600 MHz, DMSO-d_6) δ 8.39 (dd, J = 4.9, 1.2 Hz, 1H), 7.83 (td, J = 7.7, 1.9 Hz, 1H), 7.78 – 7.72 (m, 2H), 7.53 (dd, J = 9.3, 6.8 Hz, 1H), 7.31 (dd, J = 7.5, 4.9, 1.1 Hz, 1H), 7.28 – 7.22 (m, 3H), 6.46 (dd, J = 9.4, 1.2 Hz, 1H), 6.34 (dd, J = 6.9, 1.2 Hz, 1H), 4.20 (s, 2H). ^{13}C NMR (125 MHz, DMSO-d_6) δ 193.5, 165.1 (d, J = 252.7 Hz), 162.4, 151.2, 149.1, 143.7, 140.5, 138.5, 132.3, 130.8 (d, J = 9.5 Hz), 124.9, 124.1, 118.9, 115.7 (d, J = 22.1 Hz), 108.4, 42.8. ^{19}F NMR (470 MHz, CDCl$_3$) δ -103.68. HRMS (ESI) calculated for $C_{18}H_{13}FN_2O_2$ [M+H]$^+$: 308.0961; found: 308.0966.

6-(2-(4-chlorophenyl)-2-oxoethyl)-2*H*-[1,2'-bipyridin]-2-one (3al)

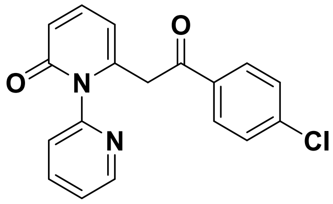

Light yellow solid (105 mg, yield: 81 %), m.p.:185-188 °C; ^{1}H NMR (600 MHz, DMSO-d_6) δ 8.38 (dd, J = 5.1, 1.9 Hz, 1H), 7.83 (td, J = 7.7, 1.9 Hz, 1H), 7.68 (d, J = 8.6 Hz, 2H), 7.53 (dd, J = 9.3, 6.8 Hz, 1H), 7.50 (d, J = 8.6 Hz, 2H), 7.31 (dd, J = 7.5, 4.9, 1.1 Hz, 1H), 7.25 (d, J = 8.0 Hz, 1H), 6.47 (d, J = 8.1 Hz, 1H), 6.34 (d, J = 5.6 Hz, 1H), 4.20 (s, 2H). ^{13}C NMR (125 MHz, DMSO-d_6) δ 194.4, 162.9, 151.7, 149.6, 144.0, 141.0, 139.0, 138.9, 134.7, 130.1, 129.3, 125.3, 124.6, 119.4, 108.9, 43.3. HRMS (ESI) calculated for $C_{18}H_{13}ClN_2O_2$ [M+H]$^+$: 324.0666; found: 324.0661.

6-(2-(4-bromophenyl)-2-oxoethyl)-2*H*-[1,2'-bipyridin]-2-one (3am)

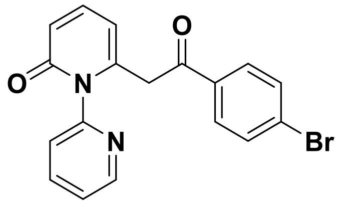

Light yellow solid (112 mg, yield: 76 %), m.p.:181-183 °C; ^{1}H NMR (500 MHz,

DMSO-d_6) δ 8.40 (dd, *J* = 4.9, 2.0, 0.8 Hz, 1H), 7.84 (td, *J* = 7.7, 1.9 Hz, 1H), 7.68 – 7.63 (m, 2H), 7.62 – 7.58 (m, 2H), 7.54 (dd, *J* = 9.3, 6.8 Hz, 1H), 7.32 (dd, *J* = 7.5, 4.9, 1.1 Hz, 1H), 7.26 (d, *J* = 8.0 Hz, 1H), 6.47 (dd, *J* = 9.3, 1.2 Hz, 1H), 6.35 (dd, *J* = 6.9, 1.2 Hz, 1H), 4.20 (s, 2H). ^{13}C NMR (125 MHz, DMSO-d_6) δ 194.6, 162.9, 151.6, 149.6, 144.0, 141.0, 139.0, 135.1, 132.2, 130.2, 128.1, 125.3, 124.6, 119.4, 108.9, 43.3. HRMS (ESI) calculated for $C_{18}H_{13}BrN_2O_2$ [M+H]$^+$: 368.0160; found: 368.0167.

6-(2-oxo-2-(4-(trifluoromethyl)phenyl)ethyl)-2*H*-[1,2'-bipyridin]-2-one (3an)

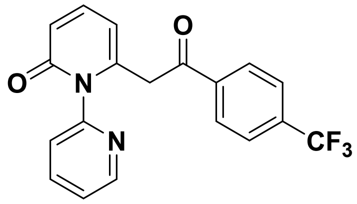

Light yellow solid (122 mg, yield: 85 %), m.p.:168-171 ^{o}C; ^{1}H NMR (600 MHz, DMSO-d_6) δ 8.38 – 8.35 (m, 1H), 7.88 – 7.79 (m, 5H), 7.55 (dd, *J* = 9.3, 6.8 Hz, 1H), 7.30 (dd, *J* = 7.5, 4.9, 1.1 Hz, 1H), 7.28 (d, *J* = 7.9 Hz, 1H), 6.48 (dd, *J* = 9.4, 1.2 Hz, 1H), 6.37 (dd, *J* = 6.8, 1.2 Hz, 1H), 4.27 (s, 2H). ^{13}C NMR (125 MHz, DMSO-d_6) δ 194.9, 162.9, 151.6, 149.6, 143.7, 141.0, 139.2, 139.0, δ 133.2 (q, *J* = 32.0 Hz), 129.1, δ 126.1 (q, *J* = 3.8 Hz), 125.3, 124.6, 124.2 (q, *J* = 374.2 Hz), 119.5, 108.9, 43.7. ^{19}F NMR (470 MHz, $CDCl_3$) δ -63.25. HRMS (ESI) calculated for $C_{19}H_{13}F_3N_2O_2$ [M+H]$^+$: 358.0929; found: 358.0919.

6-(2-oxo-2-(thiophen-2-yl)ethyl)-2*H*-[1,2'-bipyridin]-2-one (3ao)

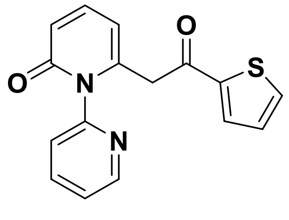

Light yellow solid (89 mg, yield: 75 %), m.p.:161-164 ^{o}C; ^{1}H NMR (600 MHz, DMSO-d_6) δ 8.42 (dd, *J* = 5.1, 1.9 Hz, 1H), 7.94 (d, *J* = 3.8 Hz, 1H), 7.82 (td, *J* = 7.7, 1.9 Hz, 1H), 7.58 (dd, *J* = 3.8, 1.2 Hz, 1H), 7.53 (dd, *J* = 9.3, 6.8 Hz, 1H), 7.31 (dd, *J* = 6.5, 4.8 Hz, 1H), 7.23 (d, *J* = 7.9 Hz, 1H), 7.10 (dd, *J* = 5.0, 3.8 Hz, 1H), 6.46 (d, *J* = 8.1 Hz, 1H), 6.37 (d, *J* = 5.6 Hz, 1H), 4.15 (s, 2H). ^{13}C NMR (125 MHz, DMSO-d_6)

δ 188.0, 162.9, 151.6, 149.6, 143.8, 142.8, 141.0, 138.9, 136.0, 134.0, 129.0, 125.4, 124.6, 119.5, 108.9, 43.5. HRMS (ESI) calculated for $C_{16}H_{12}N_2O_2S$ $[M+H]^+$: 296.0619; found: 296.0625.

6-(3-methyl-2-oxobutyl)-2*H*-[1,2'-bipyridin]-2-one (3ap)

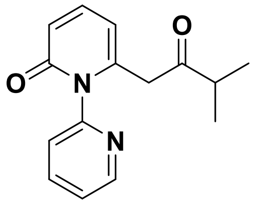

Light yellow solid (68 mg, yield: 63 %), m.p.:119-121 °C; ^{1}H NMR (500 MHz, DMSO-d_6) δ 8.60 (d, *J* = 2.9 Hz, 1H), 7.97 (t, *J* = 7.7 Hz, 1H), 7.50 (dd, *J* = 10.1, 6.6 Hz, 2H), 7.22 (d, *J* = 7.9 Hz, 1H), 6.43 (d, *J* = 9.2 Hz, 1H), 6.26 (d, *J* = 6.8 Hz, 1H), 3.71 (s, 2H), 2.23 (hept, *J* = 7.0 Hz, 1H), 0.69 (d, *J* = 6.8 Hz, 6H). ^{13}C NMR (125 MHz, DMSO-d_6) δ 208.9, 162.9, 151.7, 149.7, 144.0, 141.0, 139.1, 125.4, 124.8, 119.1, 108.5, 45.1, 39.9, 18.1. HRMS (ESI) calculated for $C_{15}H_{16}N_2O_2$ $[M+H]^+$: 256.1212; found: 256.1216.

6-(3,3-dimethyl-2-oxobutyl)-2*H*-[1,2'-bipyridin]-2-one (3aq)

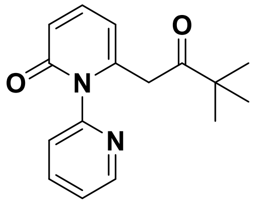

Light yellow solid (70 mg, yield: 68 %), m.p.:148-150 °C; ^{1}H NMR (500 MHz, DMSO-d_6) δ 8.61 (dd, *J* = 4.9, 1.9 Hz, 1H), 7.96 (td, *J* = 7.7, 1.9 Hz, 1H), 7.53 – 7.47 (m, 2H), 7.19 (d, *J* = 7.9 Hz, 1H), 6.42 (d, *J* = 9.3 Hz, 1H), 6.25 (d, *J* = 5.6 Hz, 1H), 3.84 (s, 2H), 0.74 (s, 9H). ^{13}C NMR (125 MHz, DMSO-d_6) δ 210.5, 163.0, 151.7, 149.7, 144.4, 140.9, 139.0, 125.6, 124.7, 119.0, 108.6, 43.9, 41.8, 26.2. HRMS (ESI) calculated for $C_{16}H_{18}N_2O_2$ $[M+H]^+$: 270.1368; found: 270.1362.

6-(2-oxo-2-phenylethyl)-1-(pyridin-2-yl)piperidin-2-one (4aa)

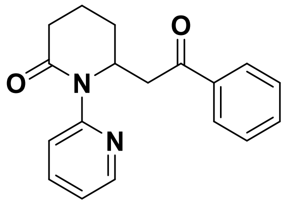

White yellow solid (41 mg, yield: 69 %), m.p.: 54-57 ^{o}C; ^{1}H NMR (600 MHz, $CDCl_3$) δ 8.43 (dd, *J* = 4.9, 1.1 Hz, 1H), 7.90 (dd, *J* = 8.3, 1.4 Hz, 2H), 7.72 – 7.67 (m, 1H), 7.62 (d, *J* = 8.2 Hz, 1H), 7.56 (t, *J* = 7.4 Hz, 1H), 7.46 – 7.41 (m, 2H), 7.11 (dd, *J* = 6.2, 4.8 Hz, 1H), 5.19 (td, *J* = 9.2, 3.9 Hz, 1H), 3.47 – 3.11 (m, 2H), 2.70 – 2.56 (m, 2H), 2.15 – 1.87 (m, 4H). ^{13}C NMR (150 MHz, $CDCl_3$) δ 197.39, 170.54, 153.15, 147.86, 136.87, 136.14, 132.89, 128.20, 127.70, 121.96, 120.87, 53.14, 42.19, 32.52, 26.71, 16.95. HRMS (ESI) calculated for $C_{16}H_{18}N_2O_2$ $[M+H]^+$: 294.1368; found: 294.1363.

(E) Gram-scale Synthesis of compound 3aa

A tube was charged with [Ru(*p*-cymene)Cl_2]$_2$ (177 mg, 5 mol%), $AgSbF_6$ (226 mg, 10 mol %), sulfoxonium ylide (**2a**, 2.28 g, 11.6 mmol), pyridone (**1a**, 1.00 g, 5.8 mmol) and HFIP (45 mL). The reaction mixture was stirred at 60 °C for 24 h under air condition. After that, the solvent was filtered through a celite pad. The filtrate was extracted with EtOAc. The organic layer was washed with brine, dried over anhydrous Na_2SO_4, and concentrated in vacuo. The residue was purified by silica gel chromatography using EtOAc/MeOH (40:1) to afford the product **3aa** as a light yellow solid (1.50 g, yield: 89 %).

(F) Synthetic Transformations of 3aa

6-(2-oxo-2-phenylethyl)-2*H*-[1,2'-bipyridin]-2-one **3aa** (0.2 mmol) was dissolved in methanol (10 mL) , 10% Pd/C was added (10 mol %). The round-bottom flask was evacuated, filled with H_2 gas and stirred 6 h at 40 ℃. The reaction mixture was filtered through a celite pad and washed with 3 x 10 mL of methanol. The solvent was removed under vacuum and the residue was purified by flash chromatography (EA/MeOH = 40/1) to give **4aa** (69 % yeild) as a colorless liquid.

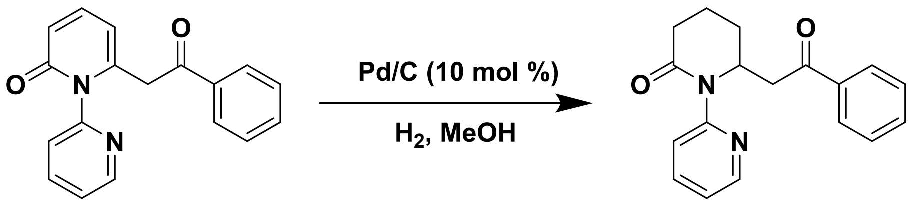

3aa **4aa 69%**

(G) Mechanistic Studies

(a) H/D Exchange Experiments

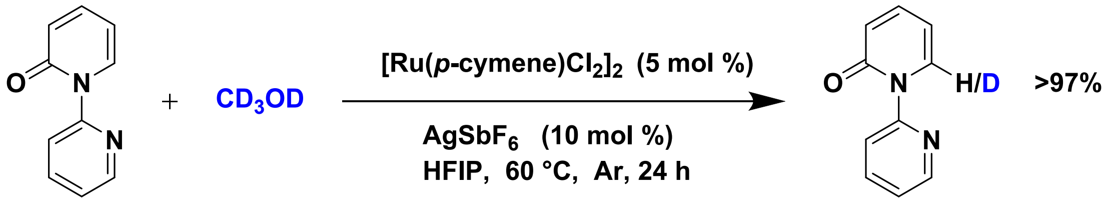

1a

A tube was charged with $[Ru(p\text{-cymene})Cl_2]_2$ (12.2 mg, 5 mol%), $AgSbF_6$ (15.6 mg, 10 mol%), pyridone (**1a**, 0.4 mmol) CD_3OD (10 equiv.) and HFIP (3 mL). The reaction mixture was stirred at 60 °C for 24 h under air condition. After that, the solvent was filtered through a celite pad. The filtrate was extracted with EtOAc. The organic layer was washed with brine, dried over anhydrous Na_2SO_4, and concentrated in vacuo. The residue was purified by silica gel chromatography using EtOAc/MeOH (40:1) to afford the product **3aa**, which was characterized by ^1H NMR spectroscopy. ^1H NMR analysis of **1a** revealed less than 3% deuteration at the 6-position of pyridone.

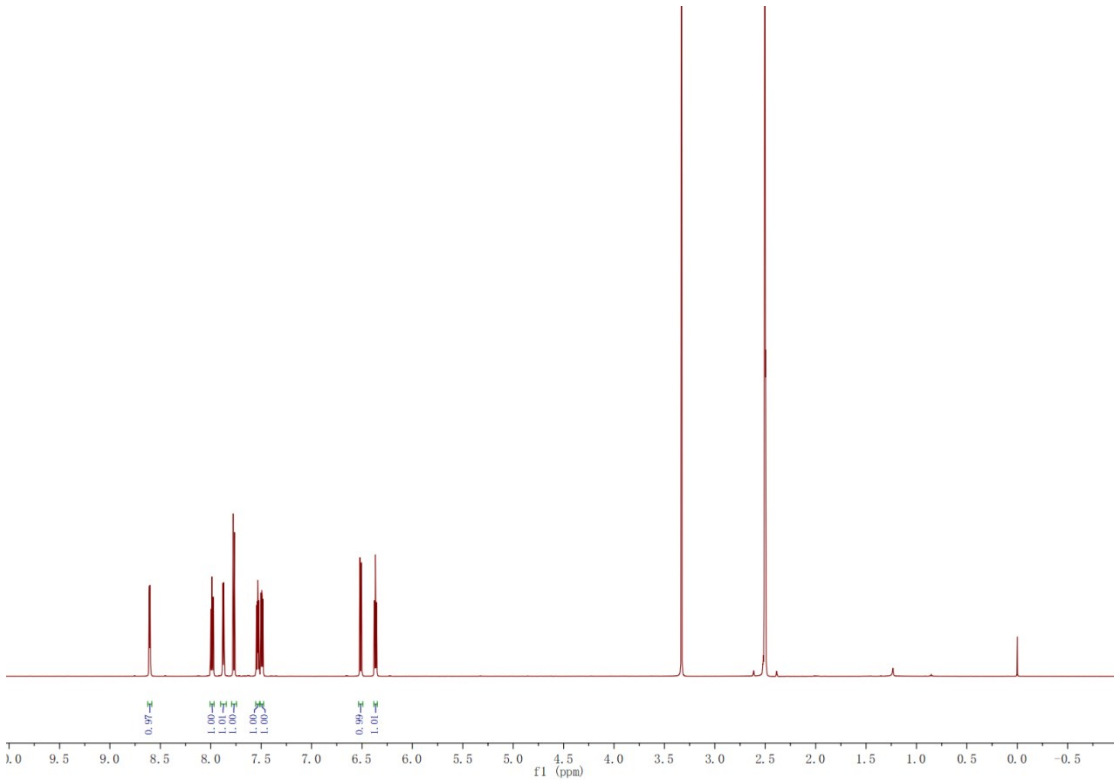

(b) KIE Experiment

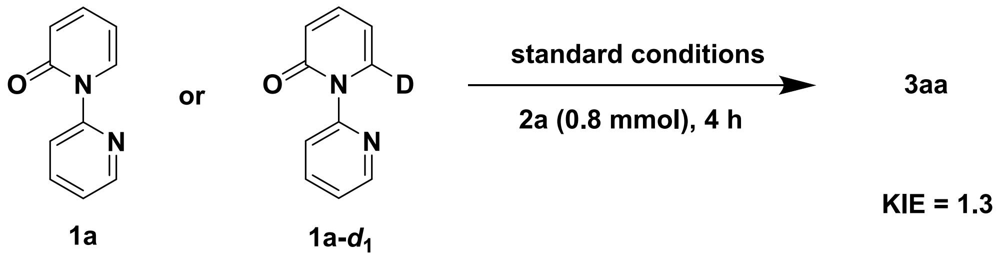

Two tubes was charged with [Ru(*p*-cymene)Cl_2]$_2$ (12.2 mg, 5 mol %), $AgSbF_6$ (15.6 mg, 10 mol %), sulfoxonium ylide (**2a**, 0.8 mmol) and HFIP (3 mL). One was added to pyridine (**1a**, 0.4 mmol), another was added to [D_1]-pyridine([D_1]-**1a**, 0.4 mmol) .The reaction mixture was stirred at 60 °C for 4 h under air condition. After that, the solvent was filtered through a celite pad. The filtrate was extracted with EtOAc. The organic layer was washed with brine, dried over anhydrous Na_2SO_4, and concentrated in vacuo. The residue was purified by silica gel chromatography using EtOAc /MeOH (40:1) to afford the product. The KIE value was determined to be k_H/k_D = 1.3.

(c) Competition Experiment

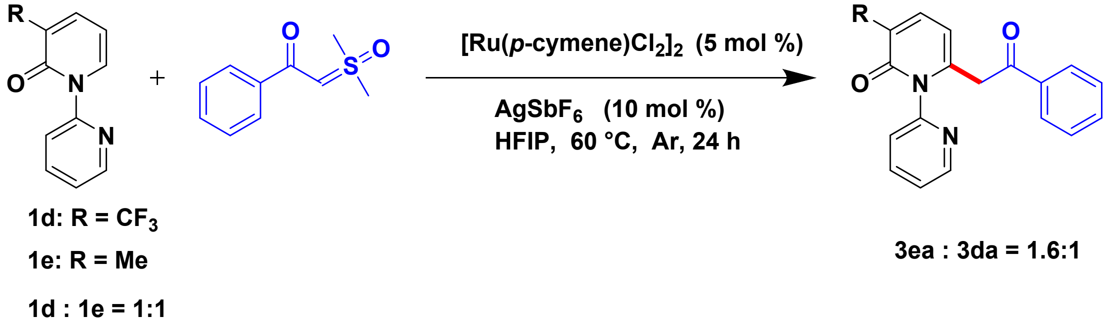

A tubes was charged with [Ru(p-cymene)Cl_2]$_2$ (6.1 mg, 5 mol %), $AgSbF_6$ (7.8 mg, 10 mol %), sulfoxonium ylide (**2a**, 0.2 mmol), **1d** (0.2 mmol), **1e** (0.4 mmol) and HFIP (3 mL). The reaction mixture was stirred at 60 °C for 24 h under air condition. After that, the solvent was filtered through a celite pad. The filtrate was extracted with EtOAc. The organic layer was washed with brine, dried over anhydrous Na_2SO_4, and concentrated in vacuo. The residue was purified by silica gel chromatography using EtOAc /MeOH (40:1) to afford a mixture of **3da** and **3ea**. The ratio of **3ea** : **3da**= 1.6 : 1 was determined on the basis of ^{1}H NMR analysis (see as below).

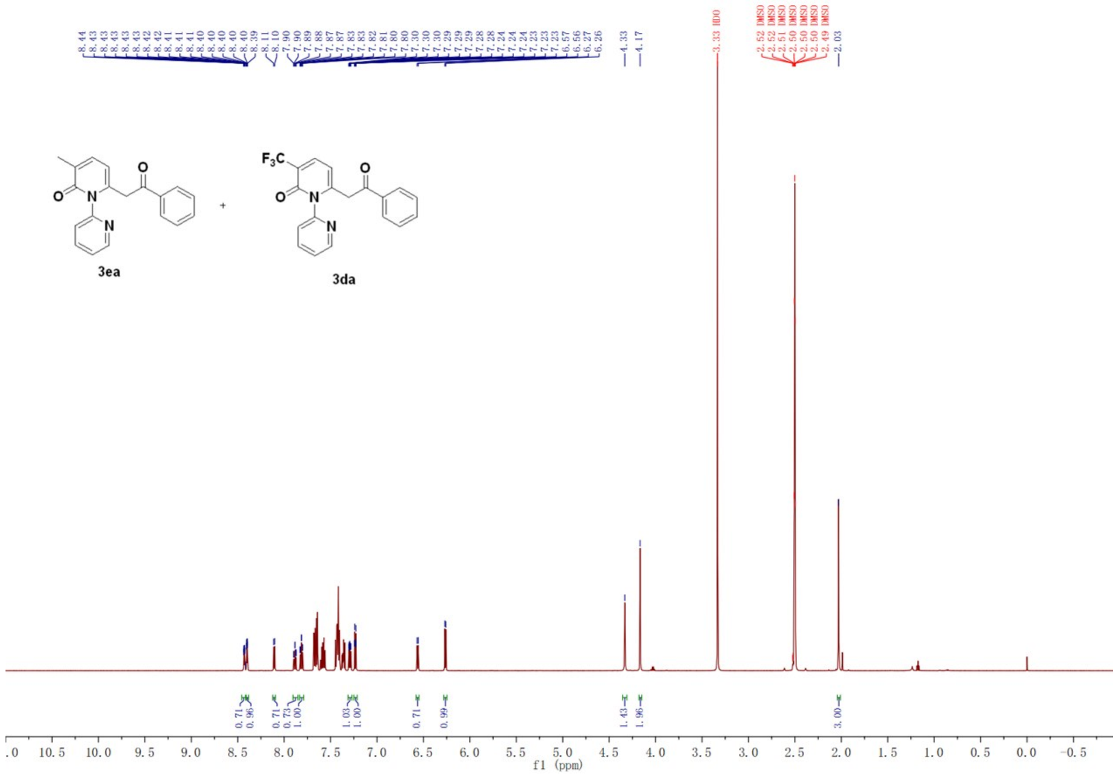

(H)Copies of ^{1}H NMR, ^{13}C NMR and ^{19}F NMR Spectra for the Products

6-(2-oxo-2-phenylethyl)-2*H*-[1,2'-bipyridin]-2-one (3aa)

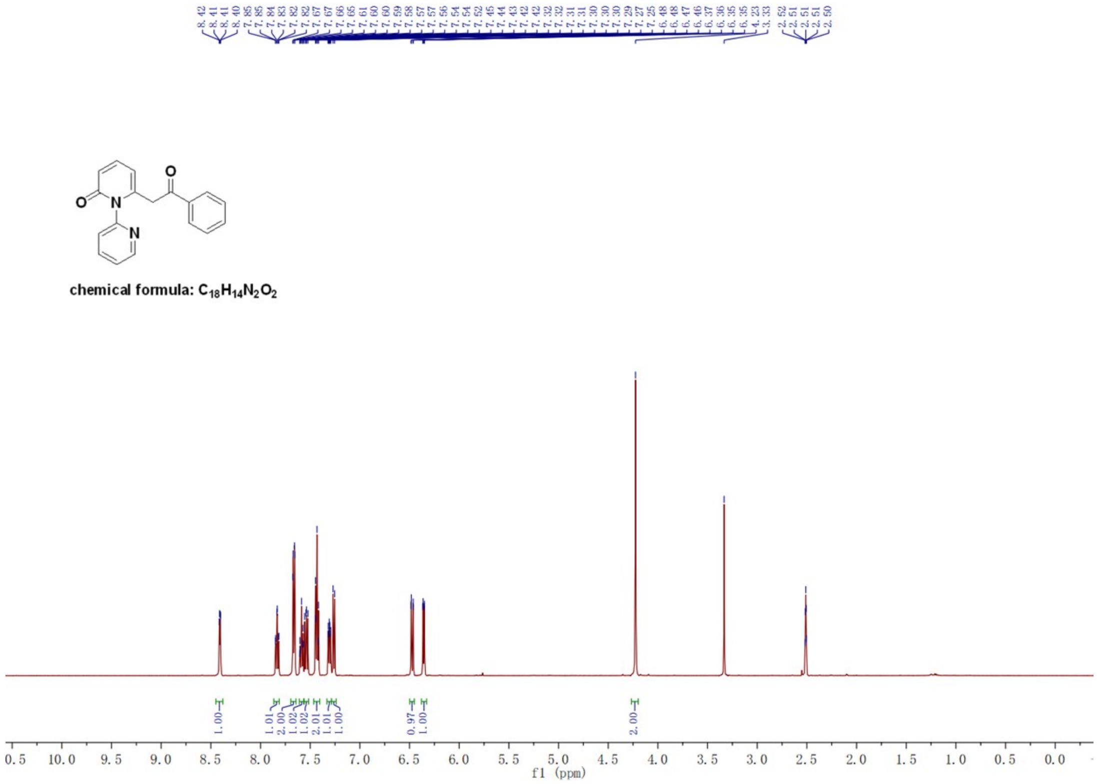

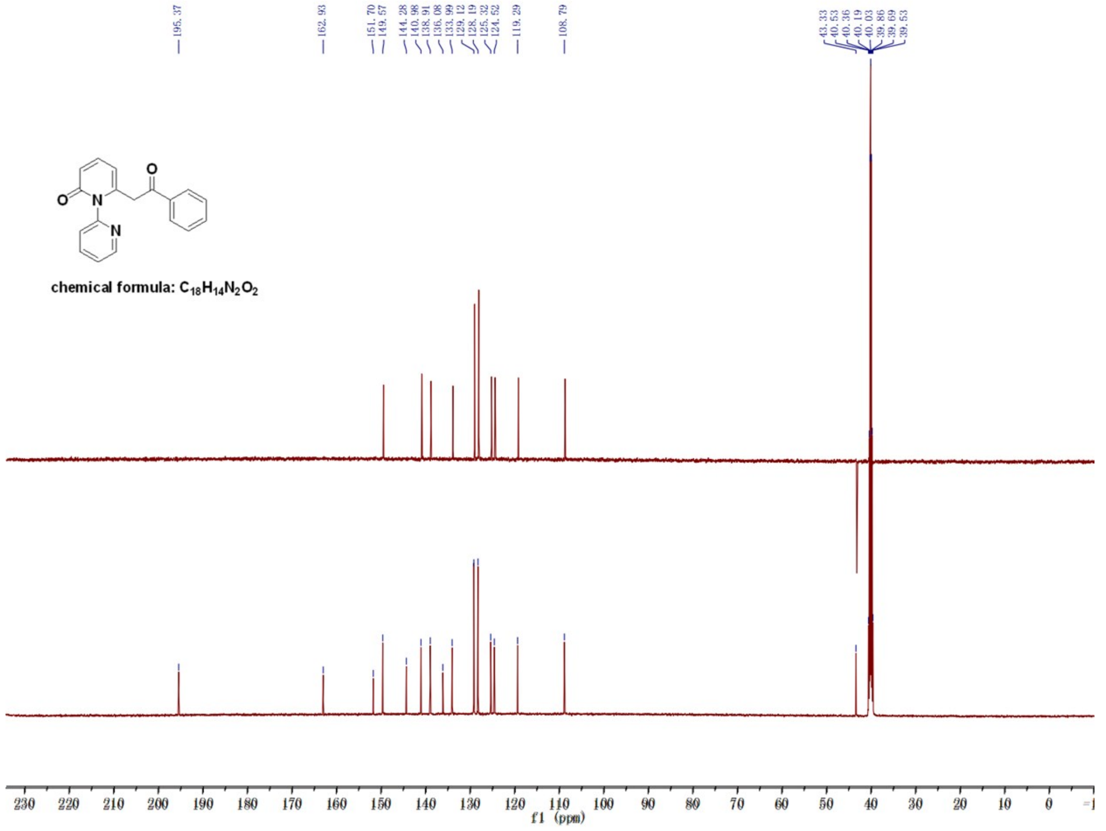

3-fluoro-6-(2-oxo-2-phenylethyl)-2*H*-[1,2'-bipyridin]-2-one (3ba)

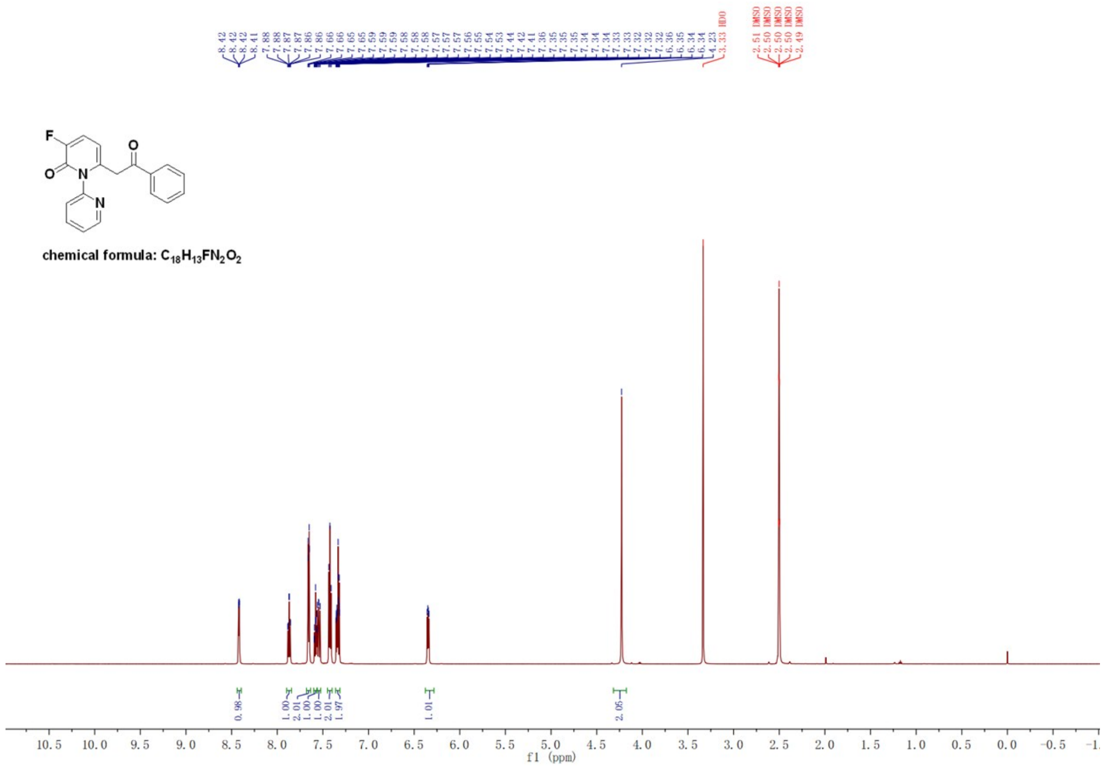

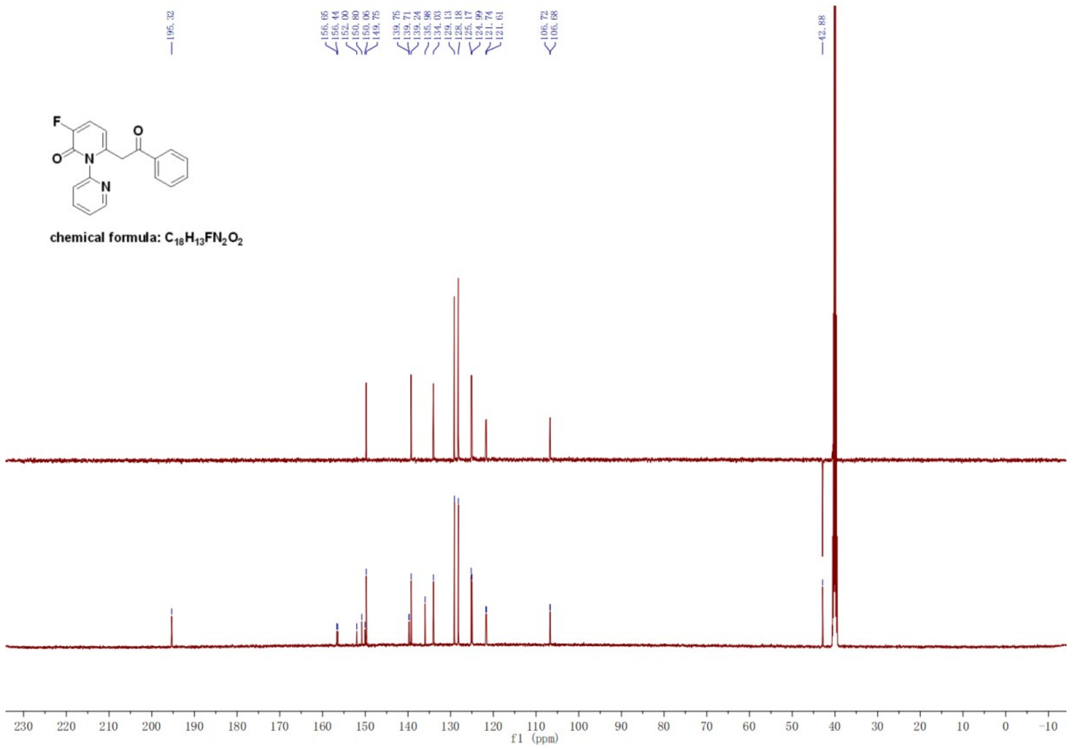

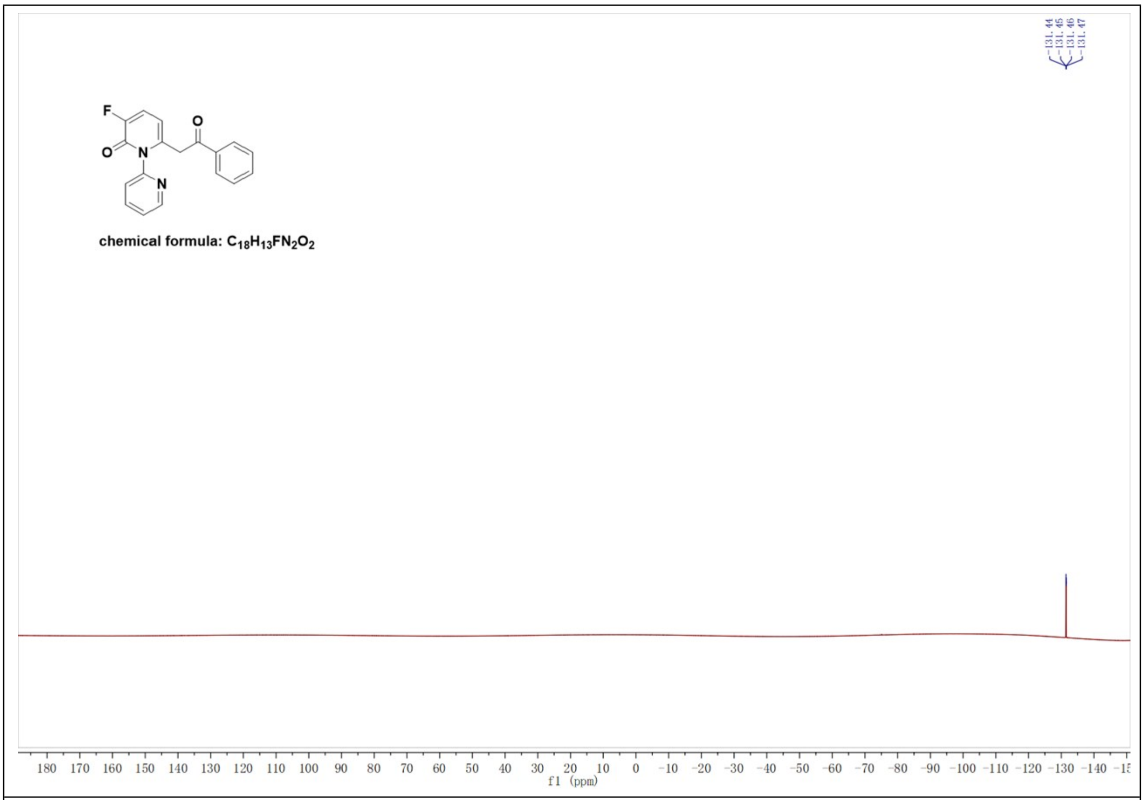

3-bromo-6-(2-oxo-2-phenylethyl)-2*H*-[1,2'-bipyridin]-2-one (3ca)

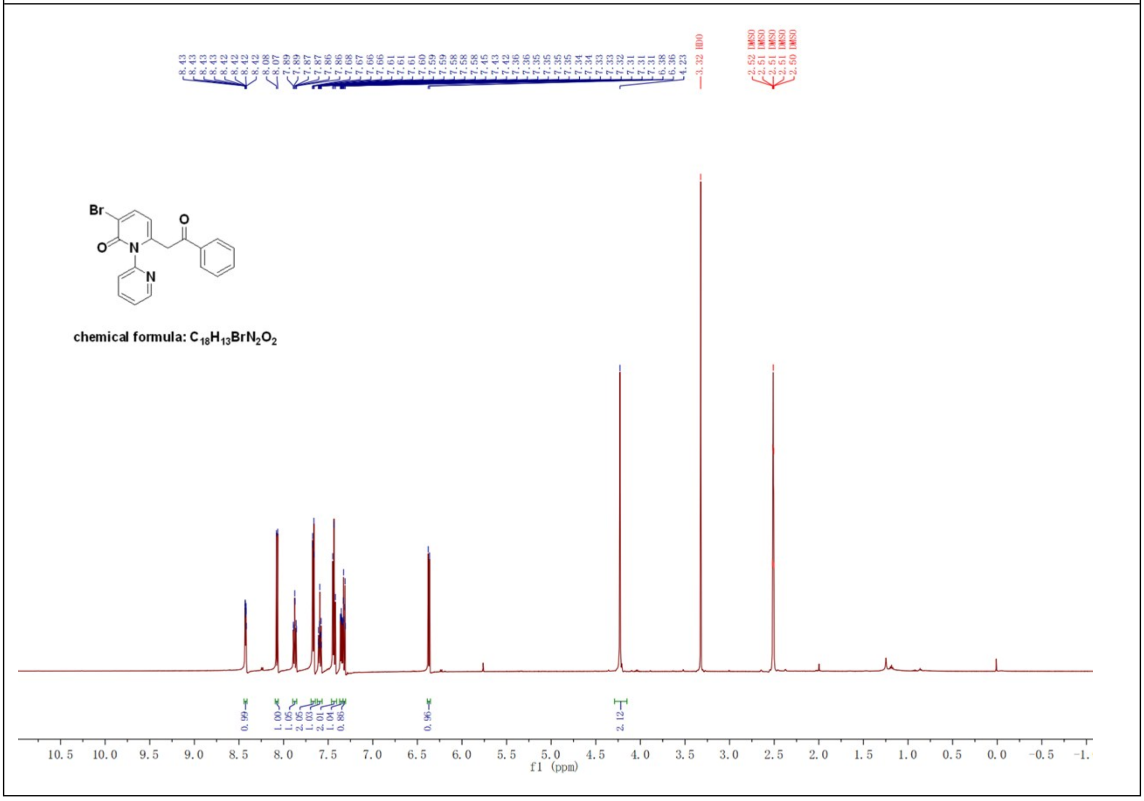

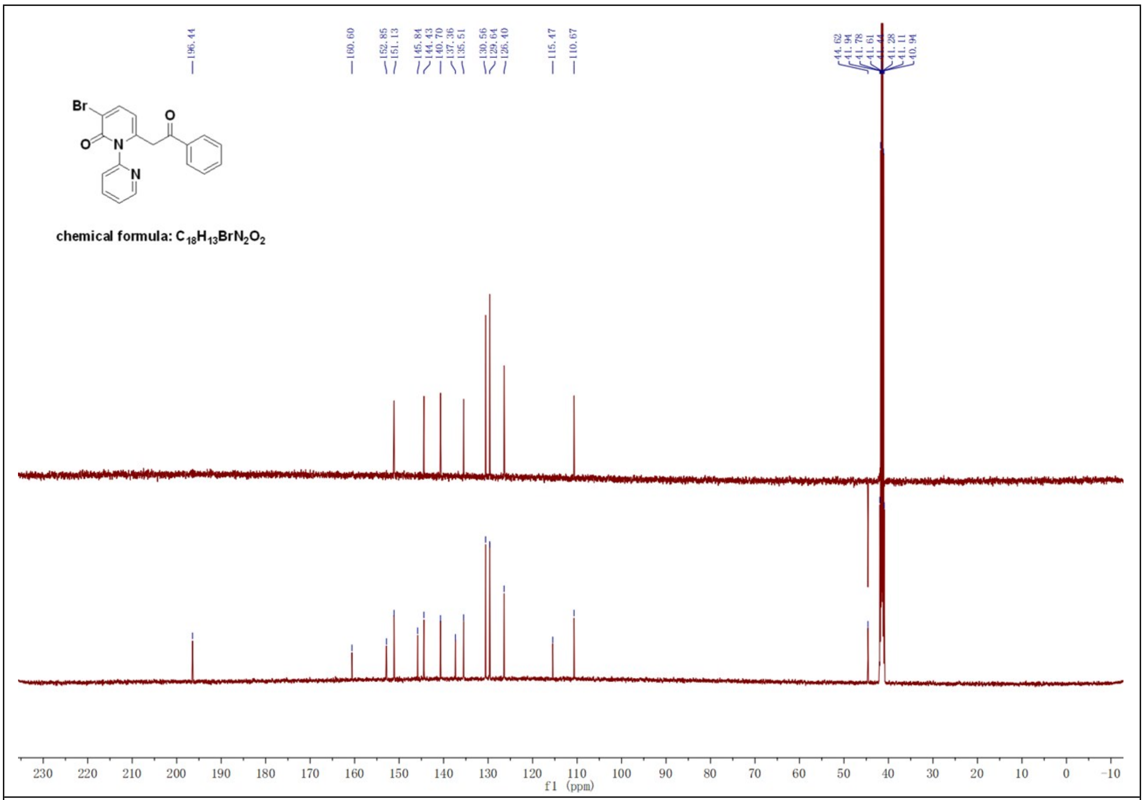

6-(2-oxo-2-phenylethyl)-3-(trifluoromethyl)-2*H*-[1,2'-bipyridin]-2-one (3da)

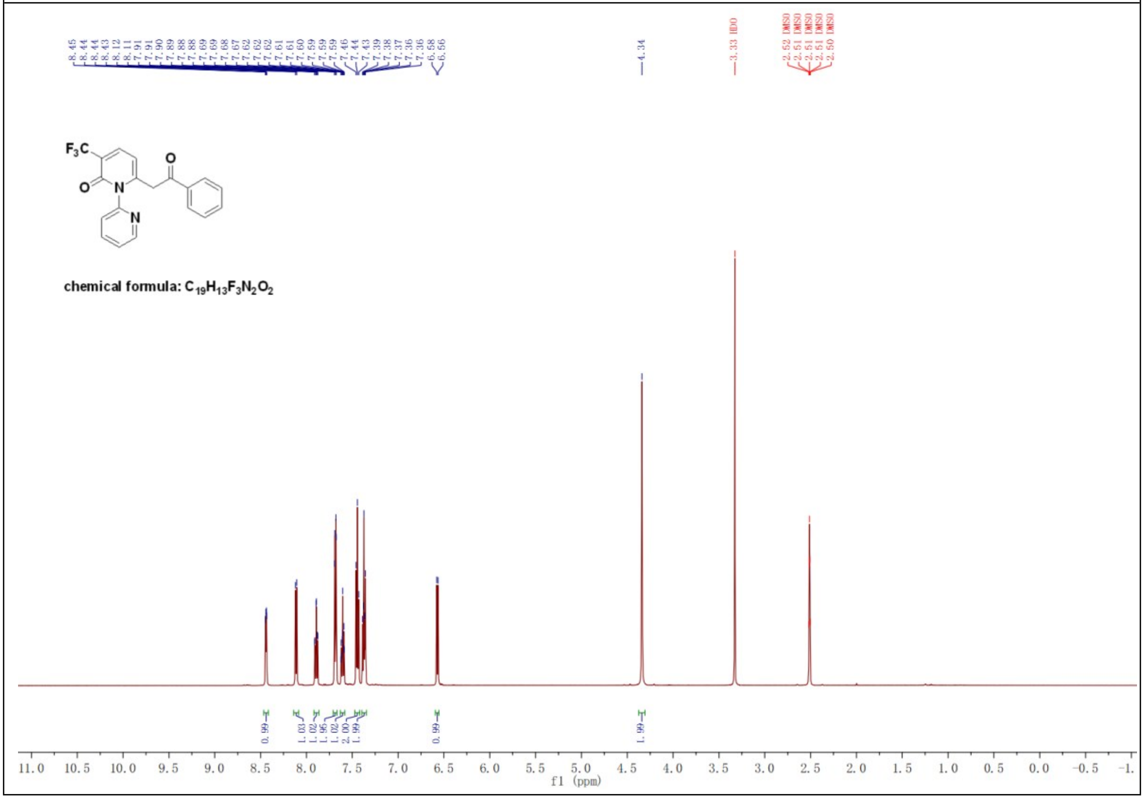

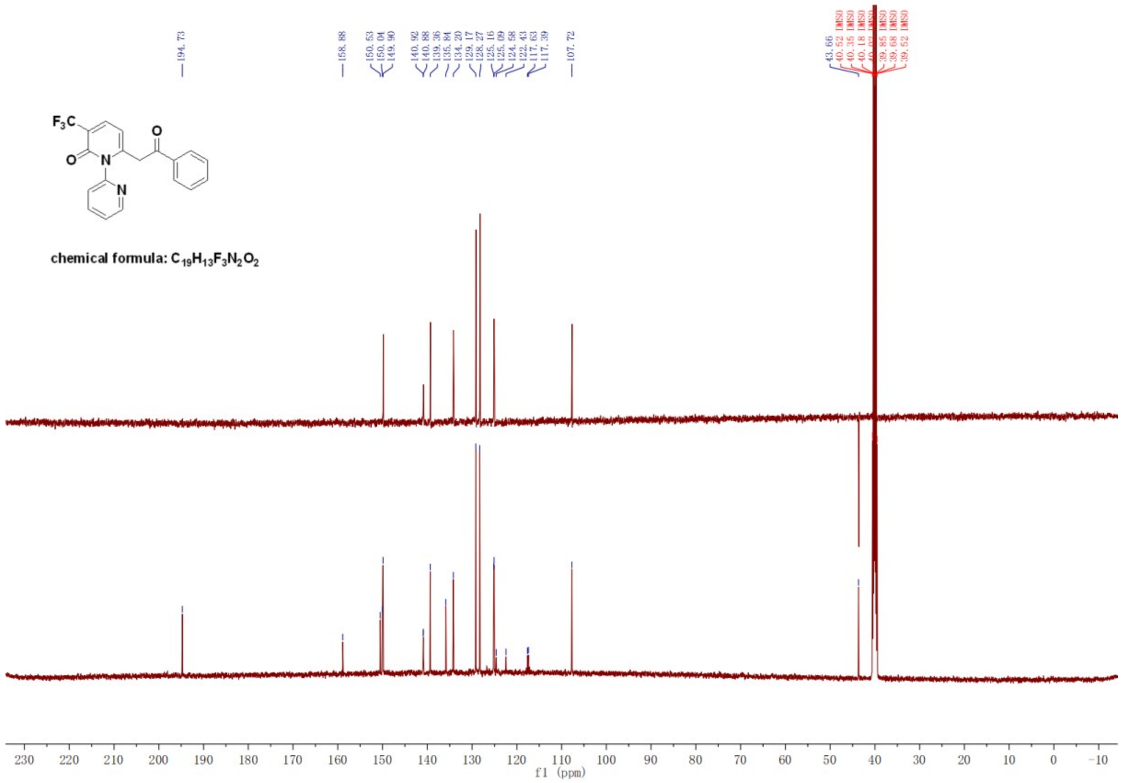
194.73
158.88
150.53
150.04
149.90
140.92
140.88
139.36
135.84
134.20
129.17
128.27
125.16
125.09
124.58
122.43
117.63
117.39
107.72
43.66
40.52 DMSO
40.35 DMSO
40.18 DMSO
39.85 DMSO
39.68 DMSO
39.52 DMSO
chemical formula: $C_{19}H_{13}F_3N_2O_2$
f1 (ppm)

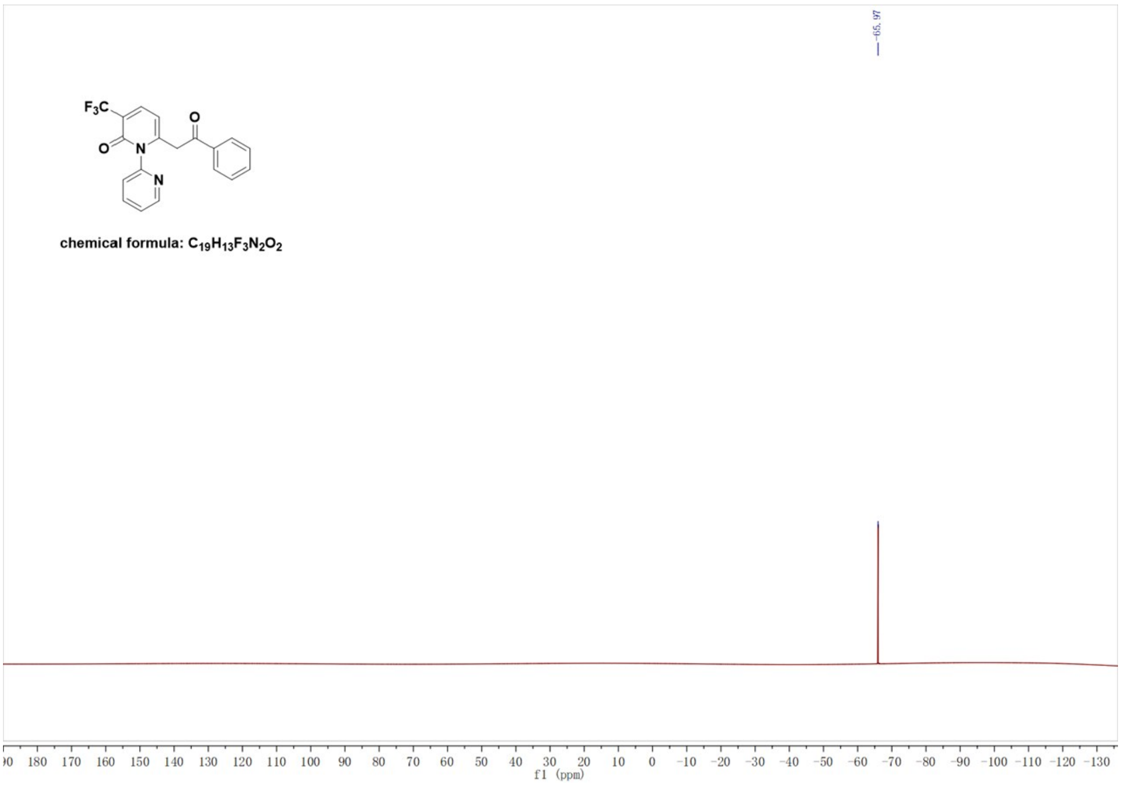
−65.97
chemical formula: $C_{19}H_{13}F_3N_2O_2$
f1 (ppm)

3-methyl-6-(2-oxo-2-phenylethyl)-2*H*-[1,2'-bipyridin]-2-one(3ea)

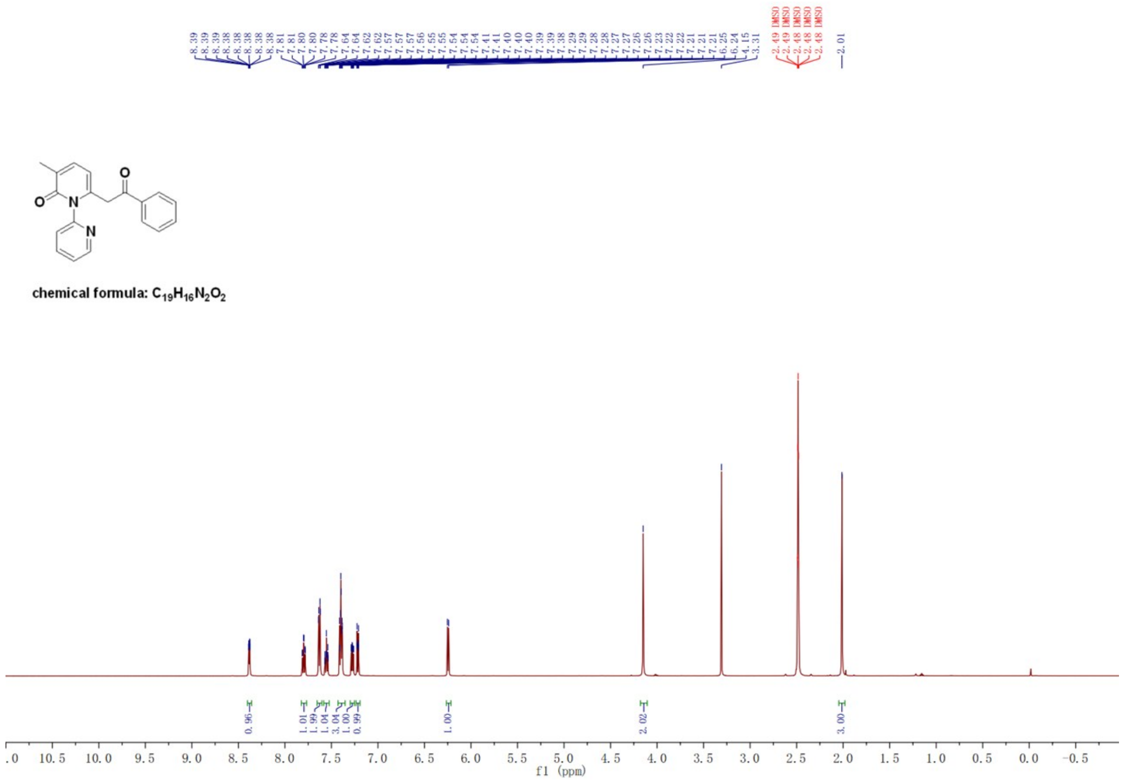

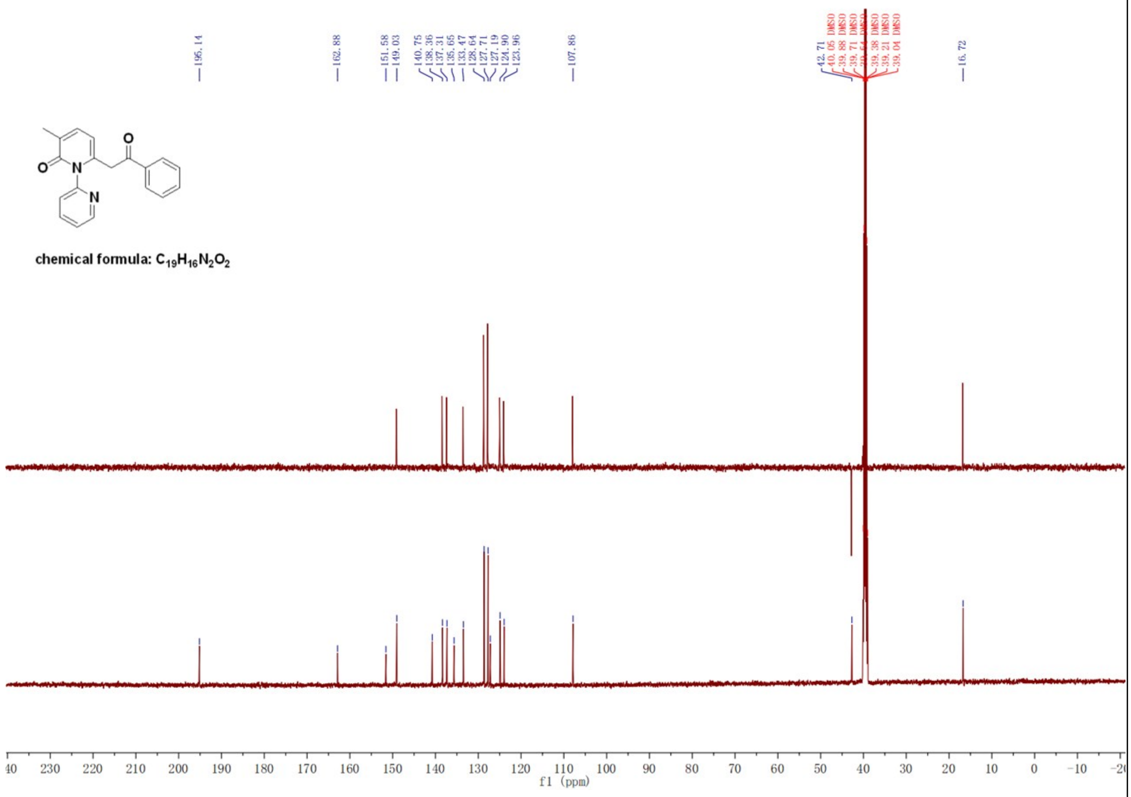

4-(benzyloxy)-6-(2-oxo-2-phenylethyl)-2*H*-[1,2'-bipyridin]-2-one (3fa)

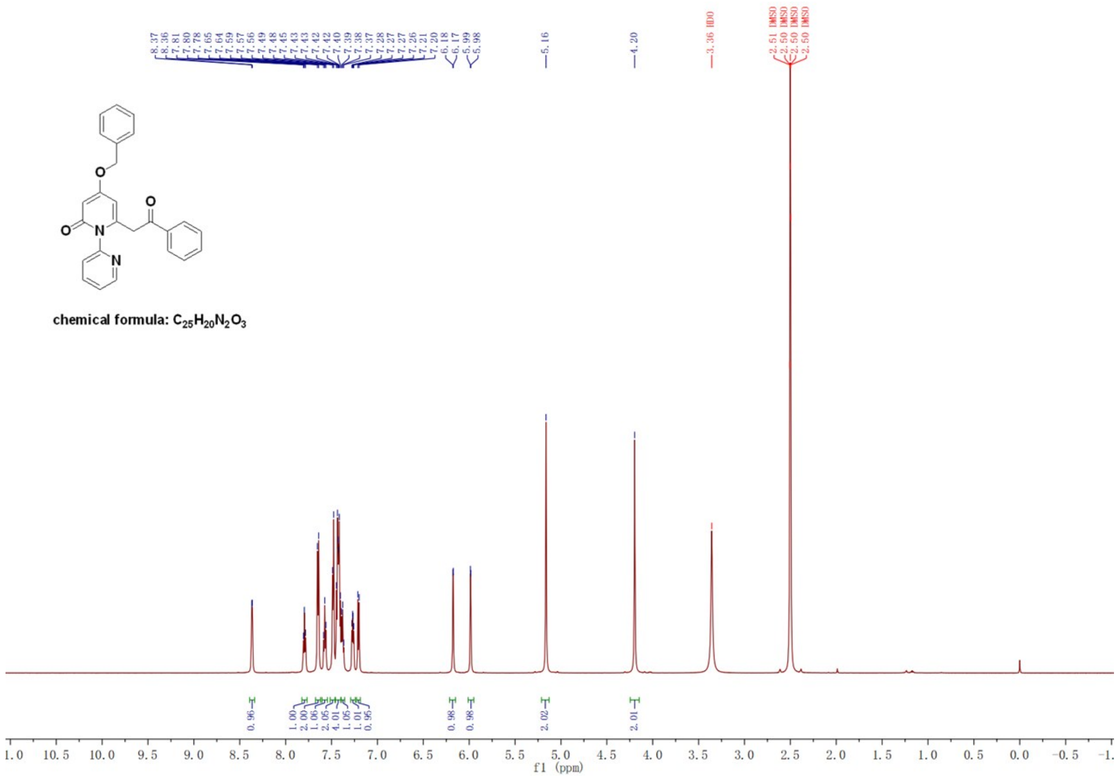

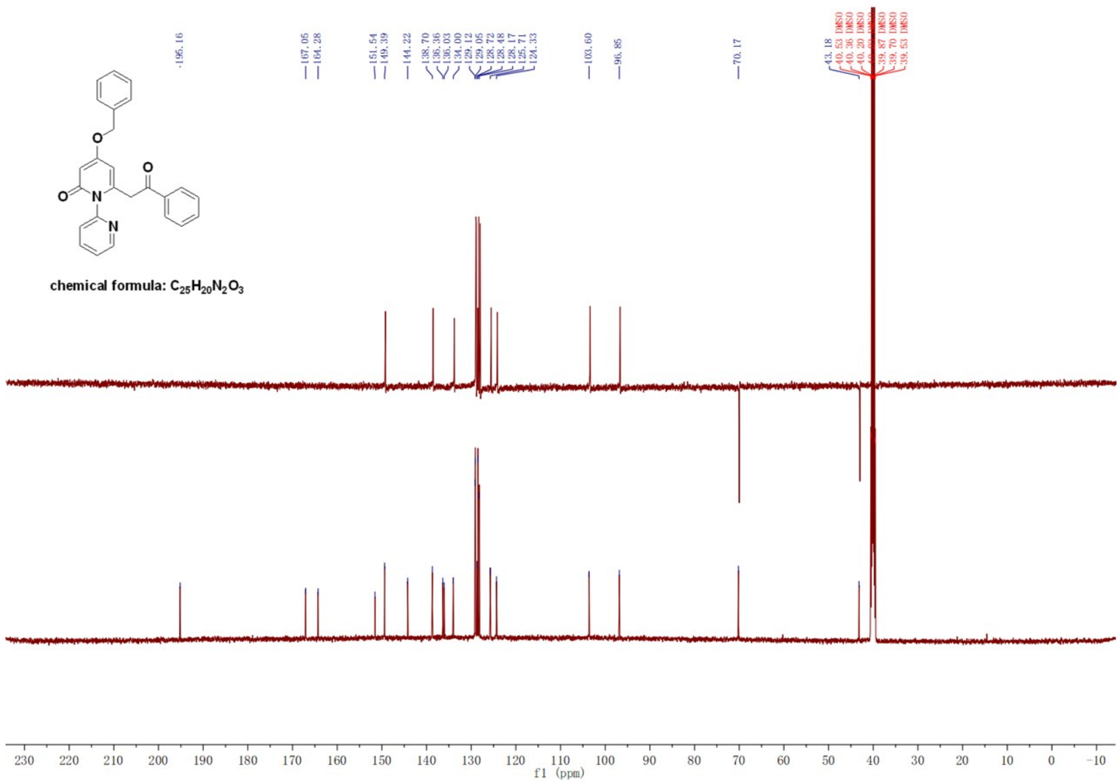

4-methyl-6-(2-oxo-2-phenylethyl)-2*H*-[1,2'-bipyridin]-2-one (3ga)

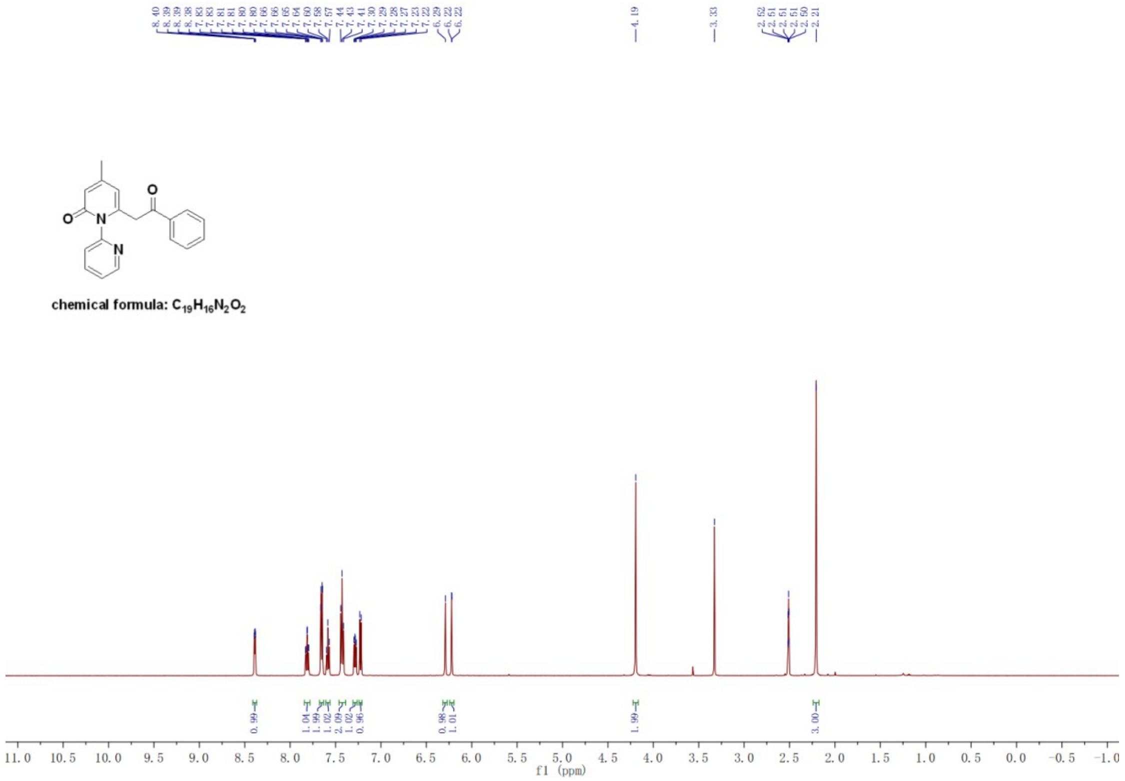

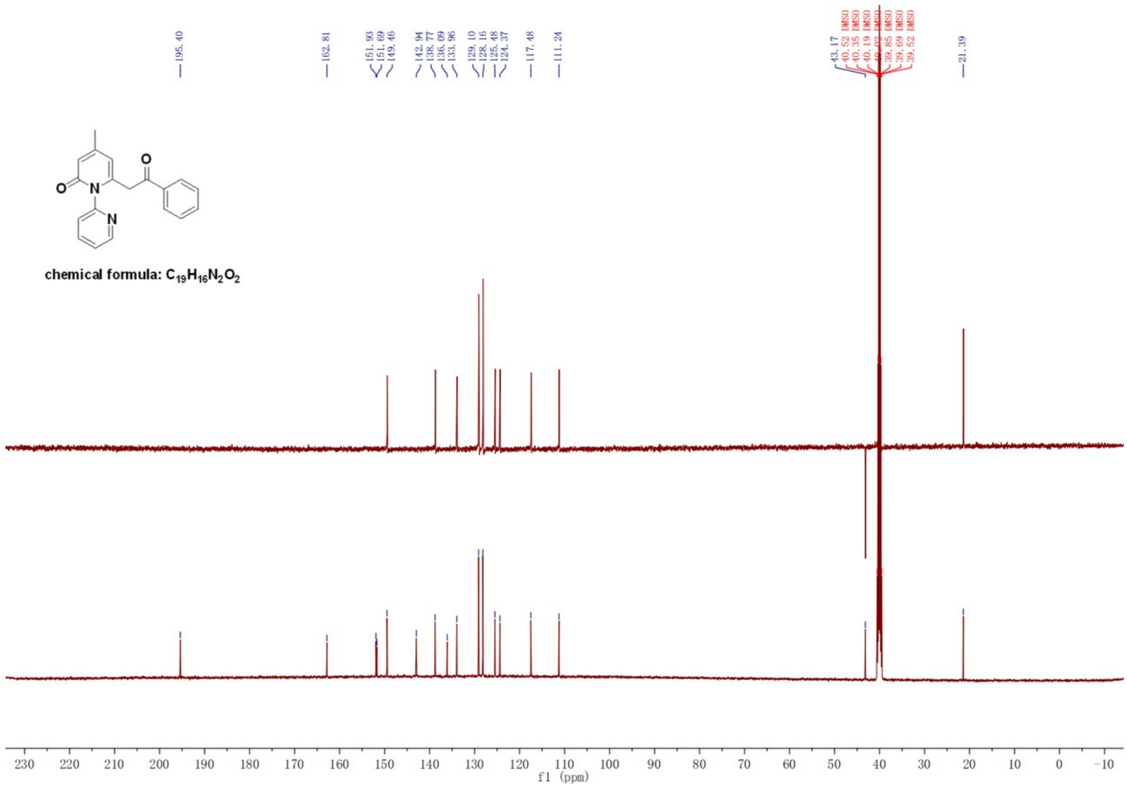

4-bromo-6-(2-oxo-2-phenylethyl)-2*H*-[1,2'-bipyridin]-2-one (3ha)

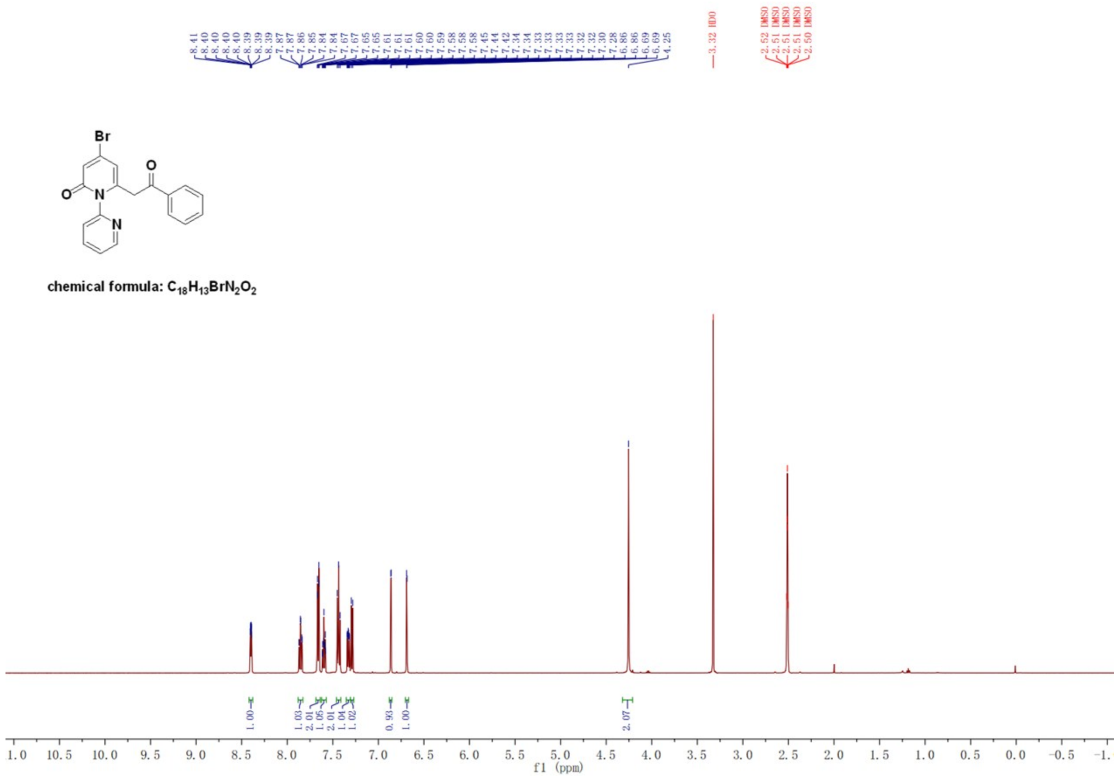

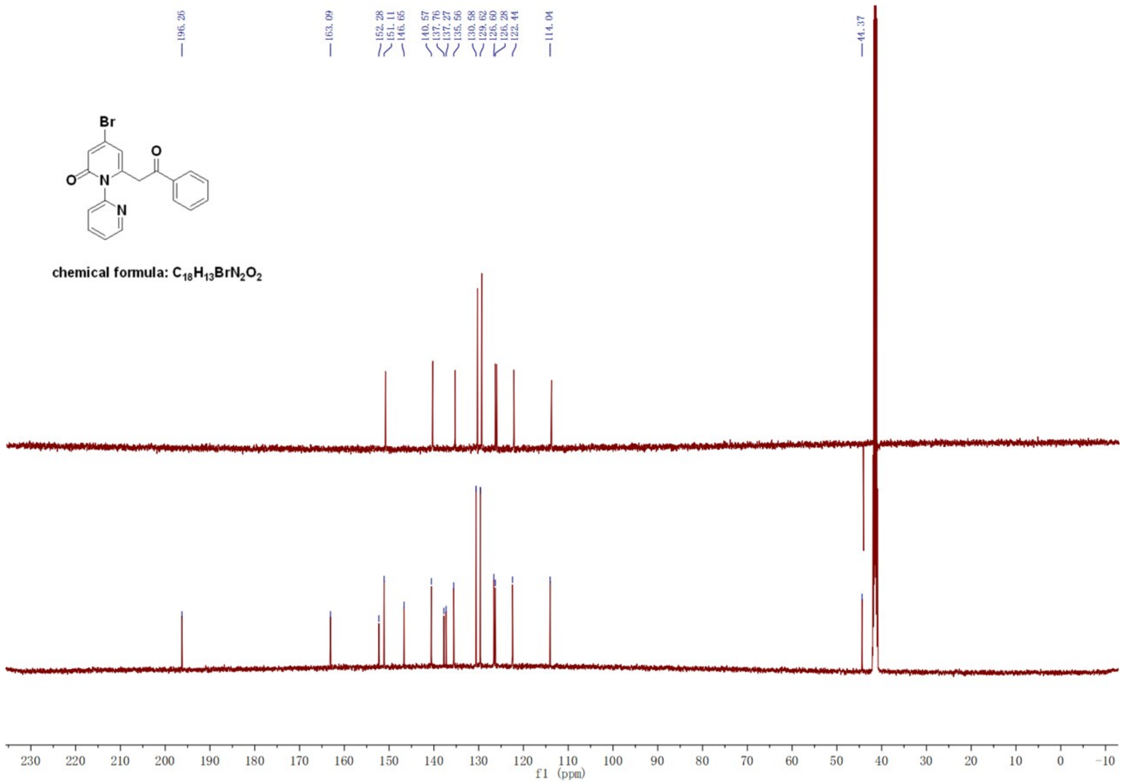

6-(2-oxo-2-phenylethyl)-4-(trifluoromethyl)-2*H*-[1,2'-bipyridin]-2-one (3ia)

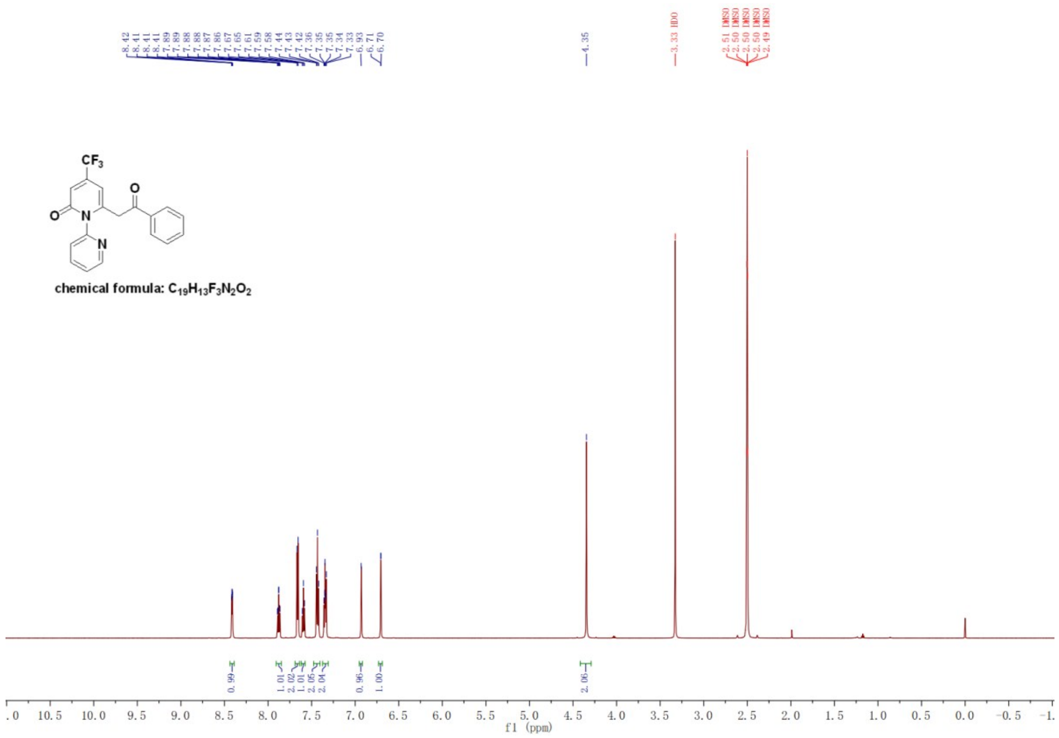

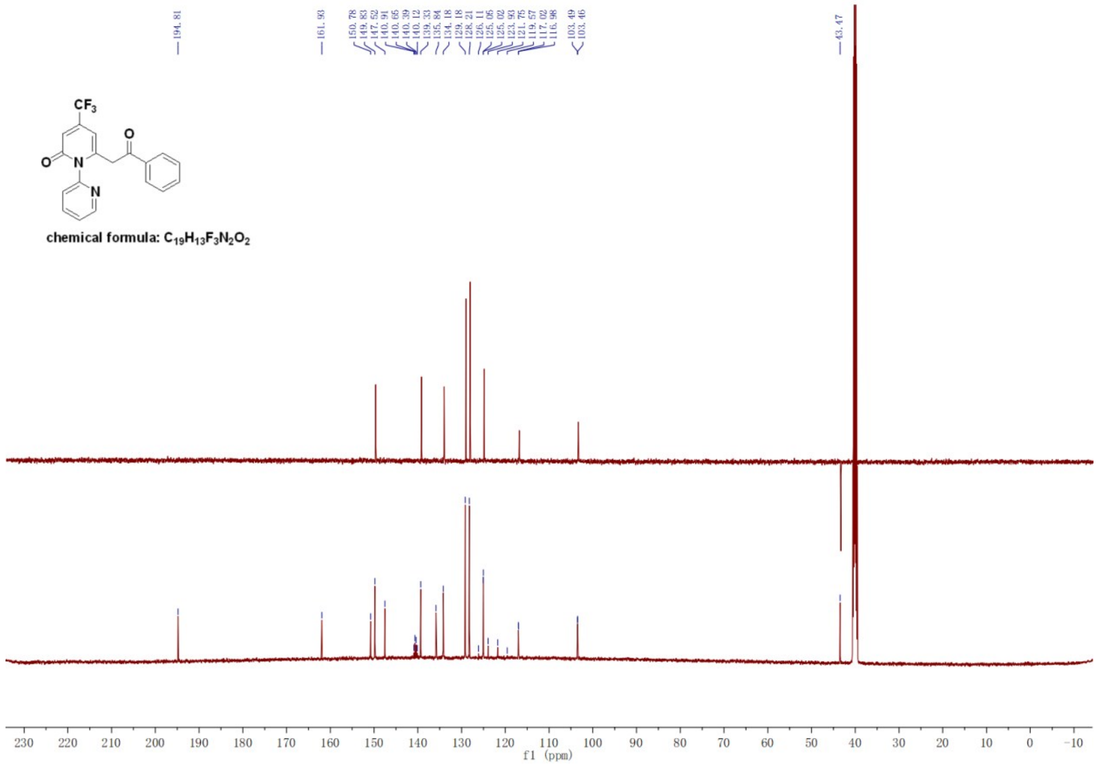

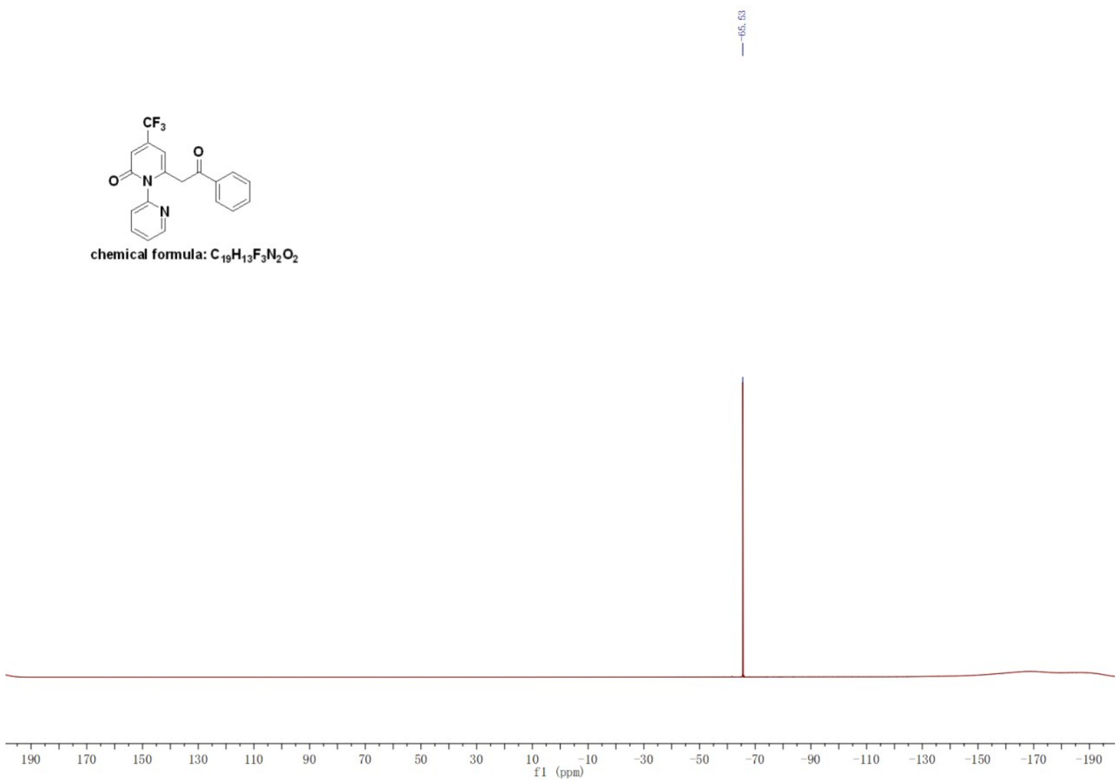

5-methyl-6-(2-oxo-2-phenylethyl)-2*H*-[1,2'-bipyridin]-2-one (3ja)

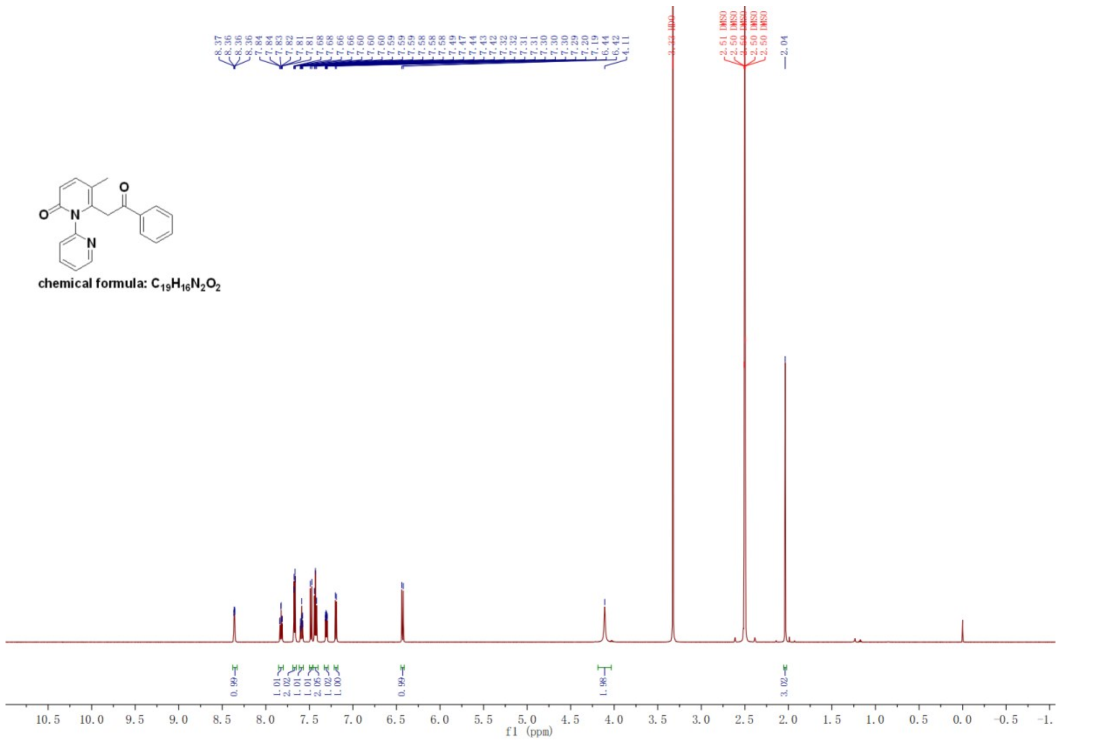

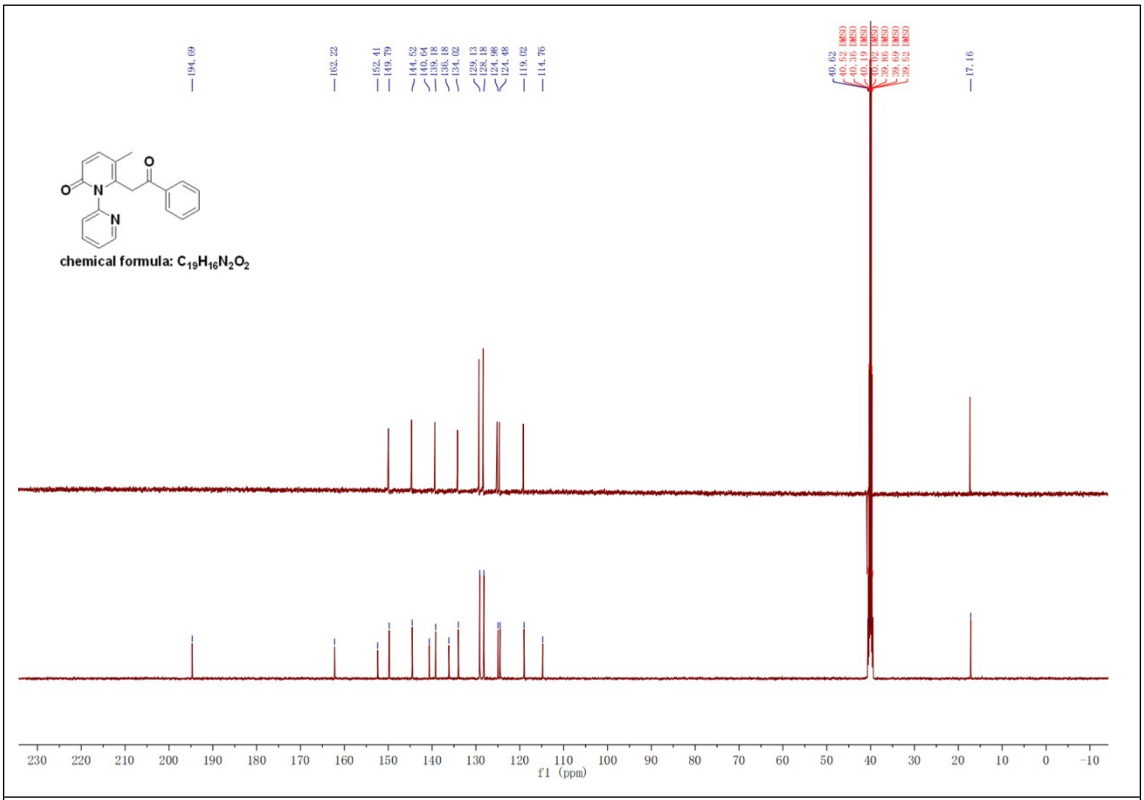

5-fluoro-6-(2-oxo-2-phenylethyl)-2*H*-[1,2'-bipyridin]-2-one (3ka)

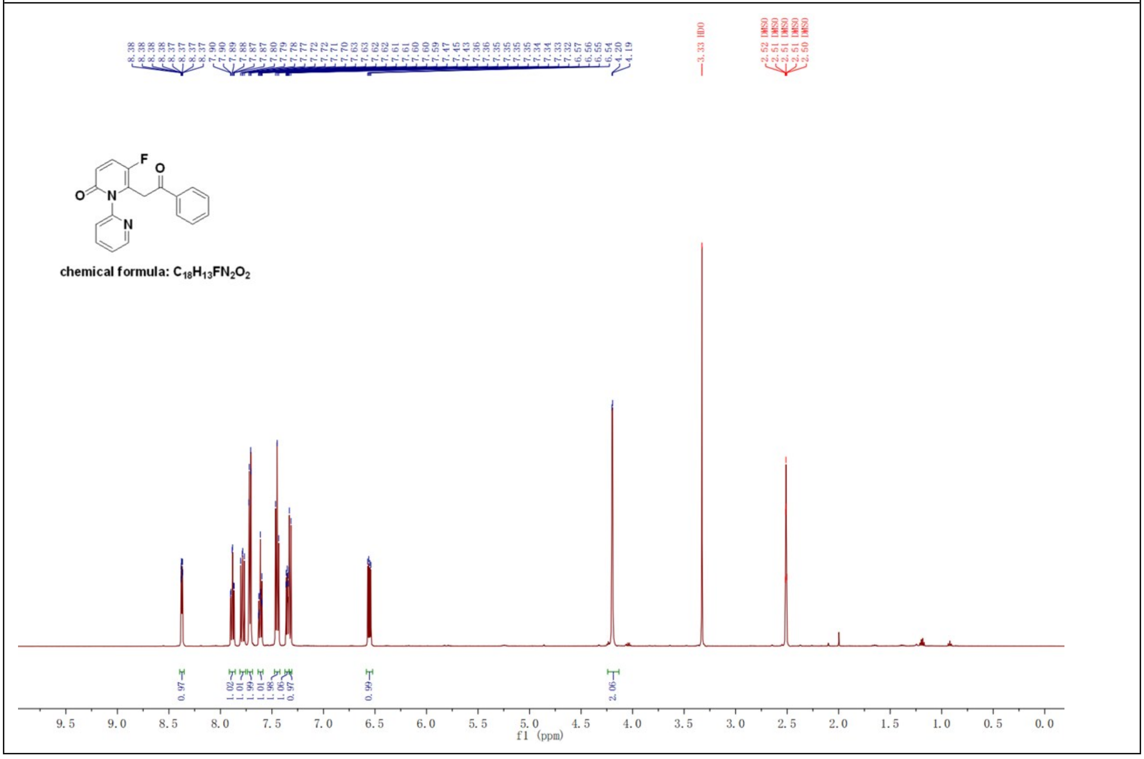

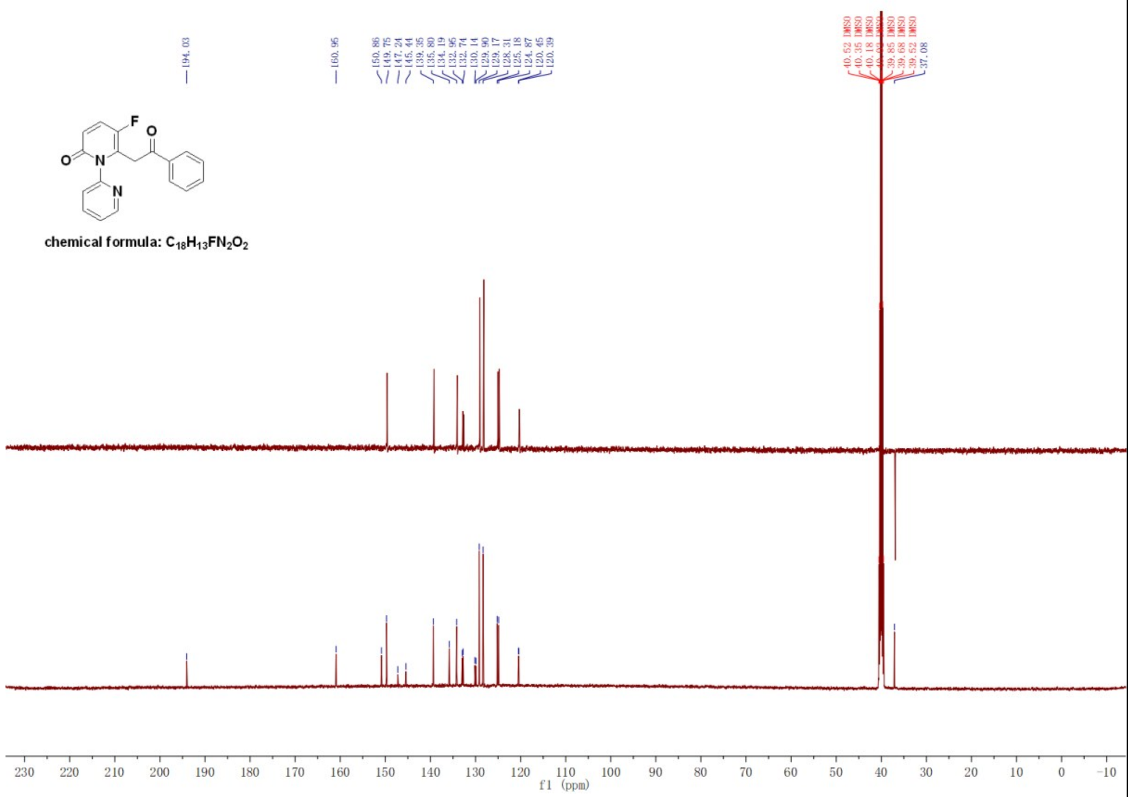
194.03
160.95
150.86
149.75
147.24
145.44
139.35
135.80
134.19
132.95
132.74
130.14
129.90
129.17
128.31
125.18
124.87
120.45
120.39
40.52 DMSO
40.35 DMSO
40.18 DMSO
39.85 DMSO
39.68 DMSO
39.52 DMSO
37.08
F
O
O
N
N
chemical formula: $C_{18}H_{13}FN_2O_2$
230 220 210 200 190 180 170 160 150 140 130 120 110 100 90 80 70 60 50 40 30 20 10 0 -10
f1 (ppm)

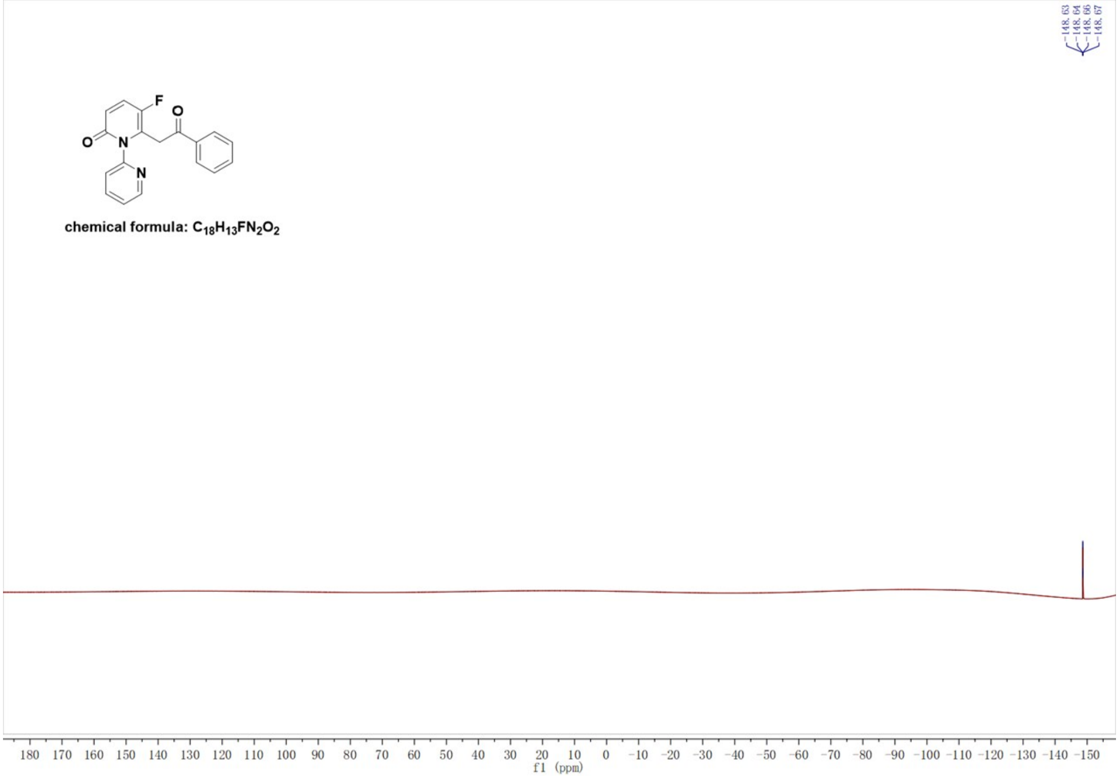
-148.63
-148.64
-148.66
-148.67
F
O
O
N
N
chemical formula: $C_{18}H_{13}FN_2O_2$
180 170 160 150 140 130 120 110 100 90 80 70 60 50 40 30 20 10 0 -10 -20 -30 -40 -50 -60 -70 -80 -90 -100 -110 -120 -130 -140 -150
f1 (ppm)

5-chloro-6-(2-oxo-2-phenylethyl)-2*H*-[1,2'-bipyridin]-2-one (3la)

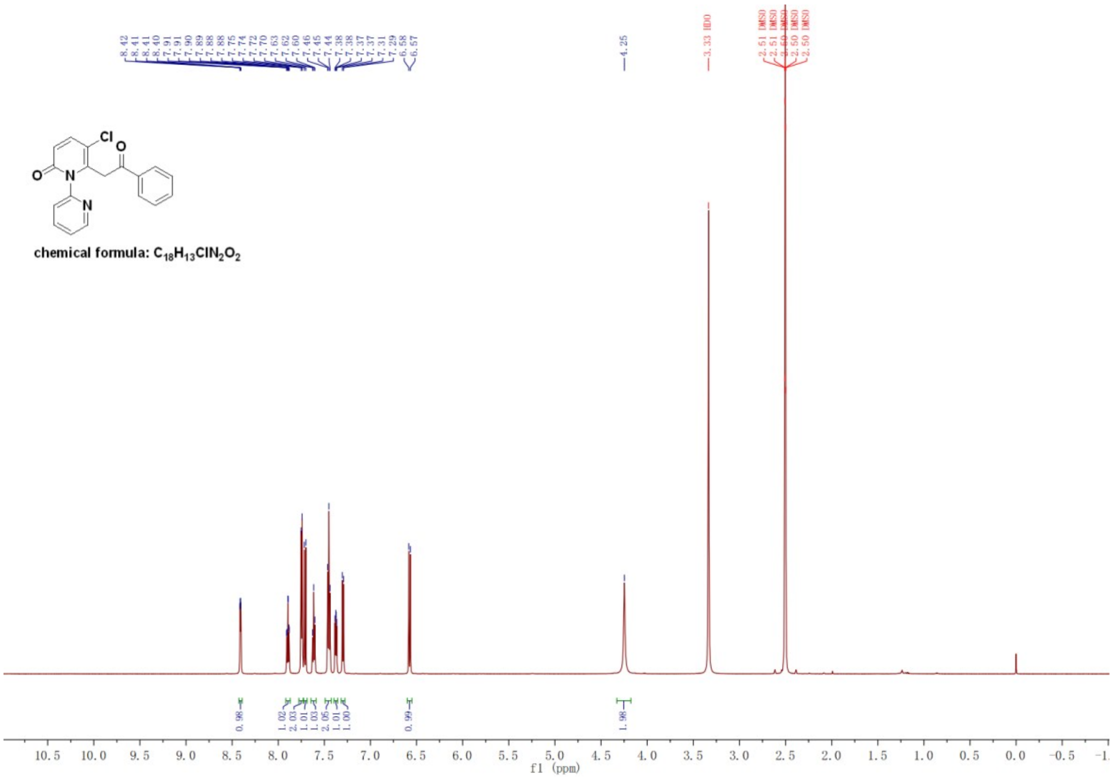

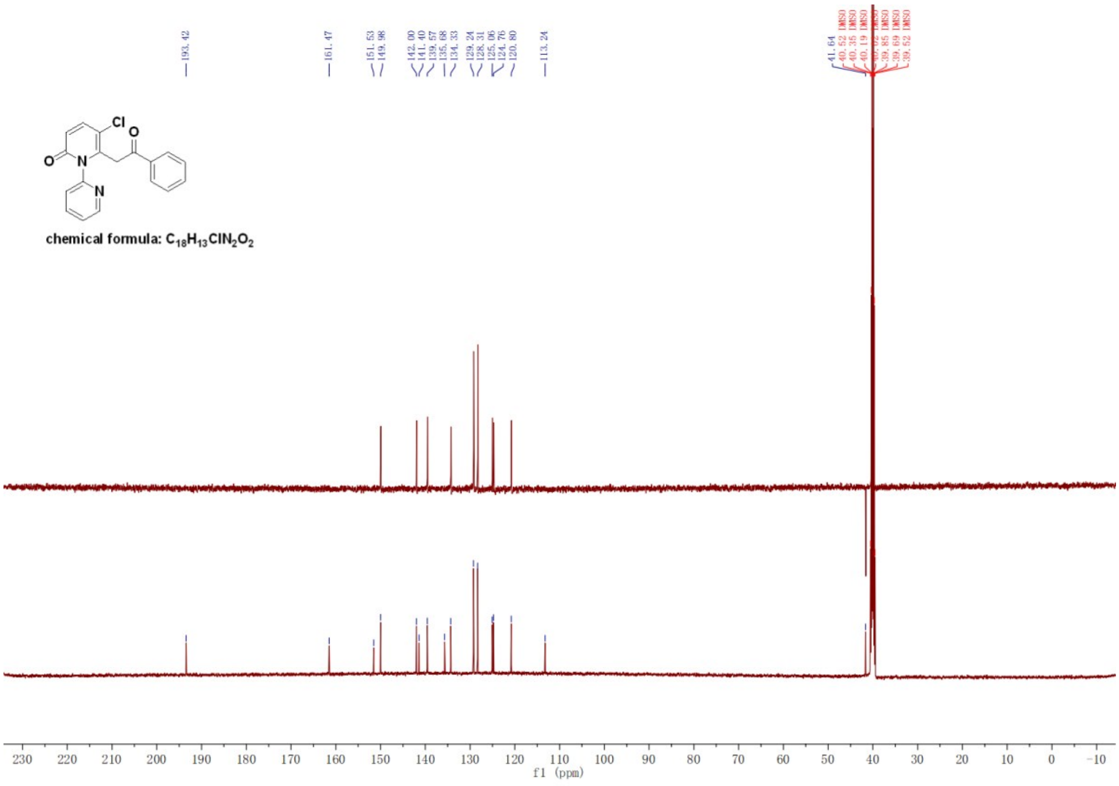

5-bromo-6-(2-oxo-2-phenylethyl)-2*H*-[1,2'-bipyridin]-2-one (3ma)

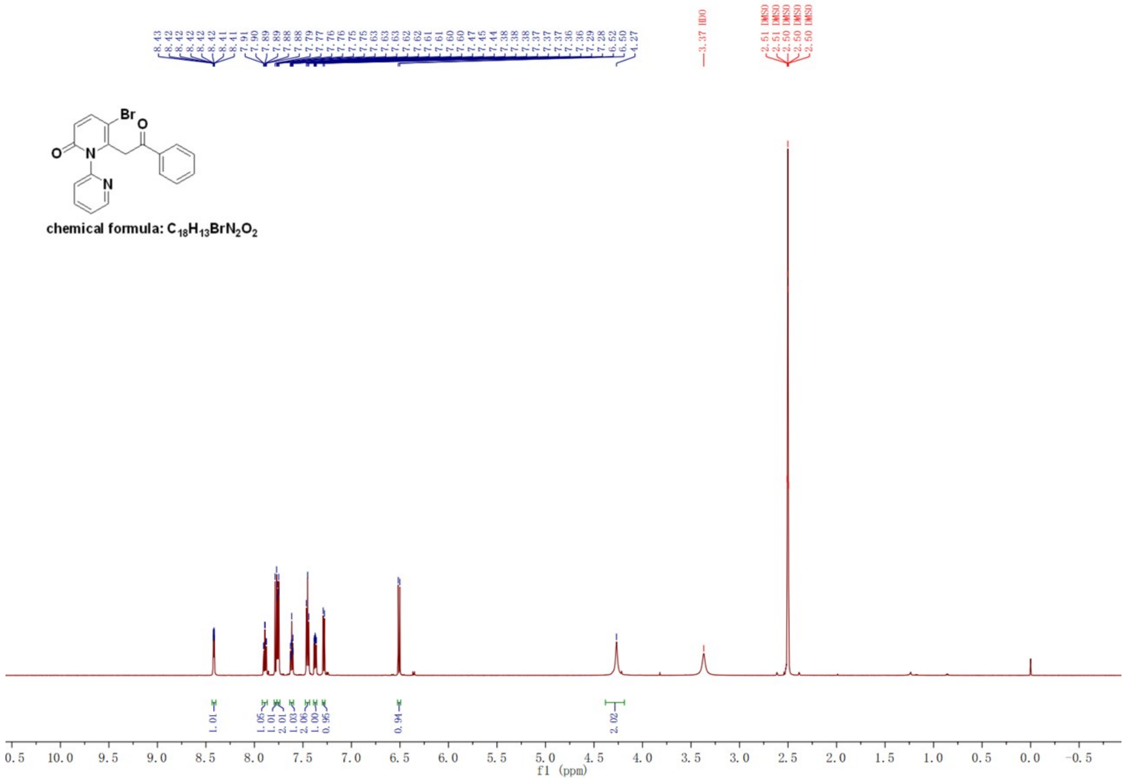

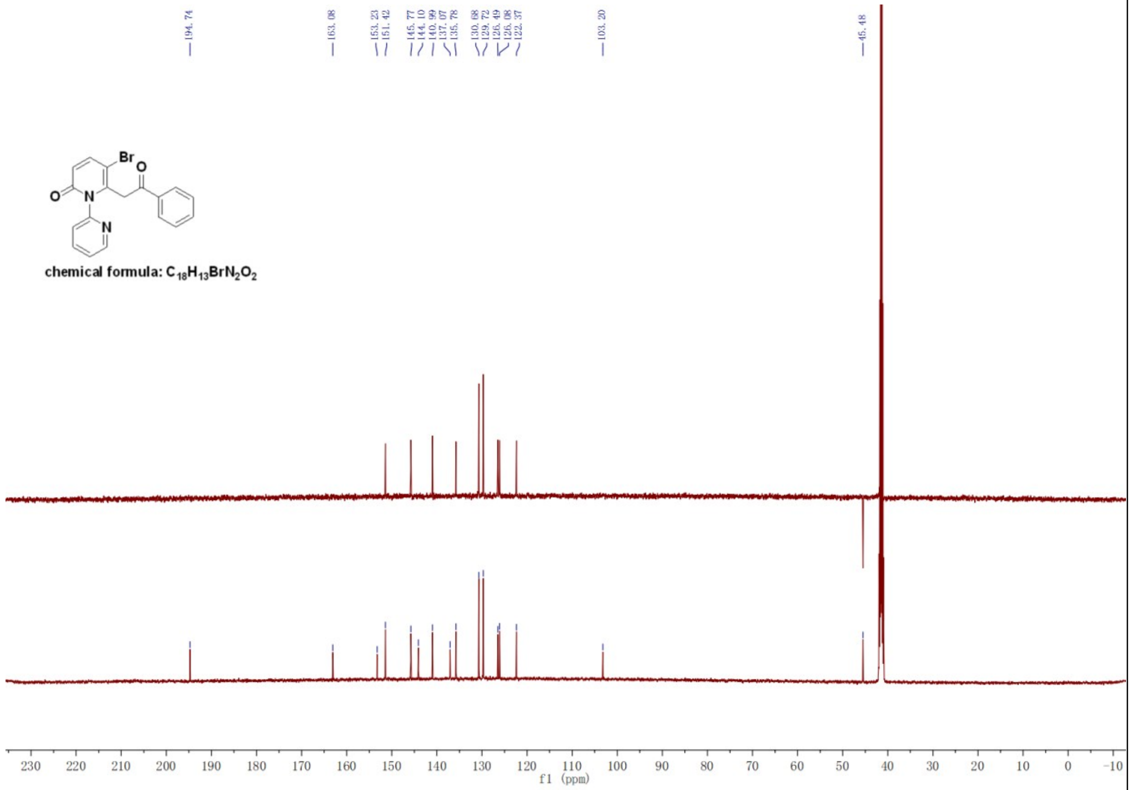

3-(2-oxo-2-phenylethyl)-2-(pyridin-2-yl)isoquinolin-1(2*H*)-one (3na)

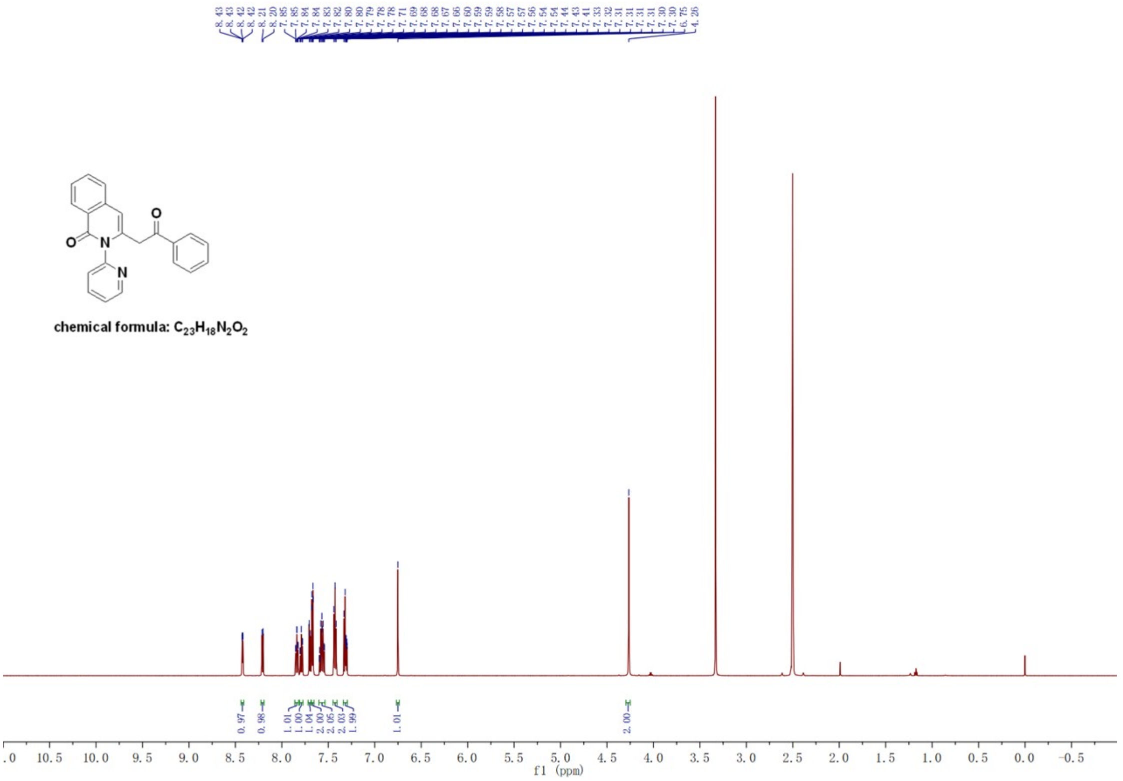

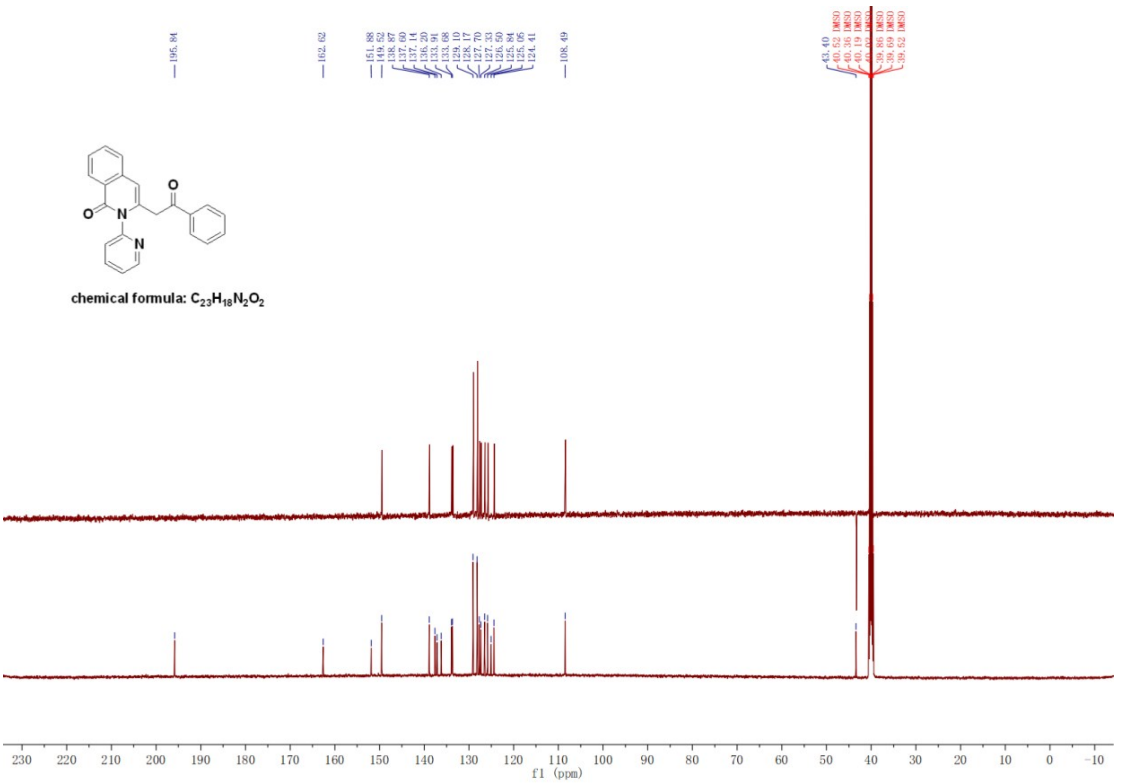

6-methoxy-3-(2-oxo-2-phenylethyl)-2-(pyridin-2-yl)isoquinolin-1(2*H*)-one (3oa)

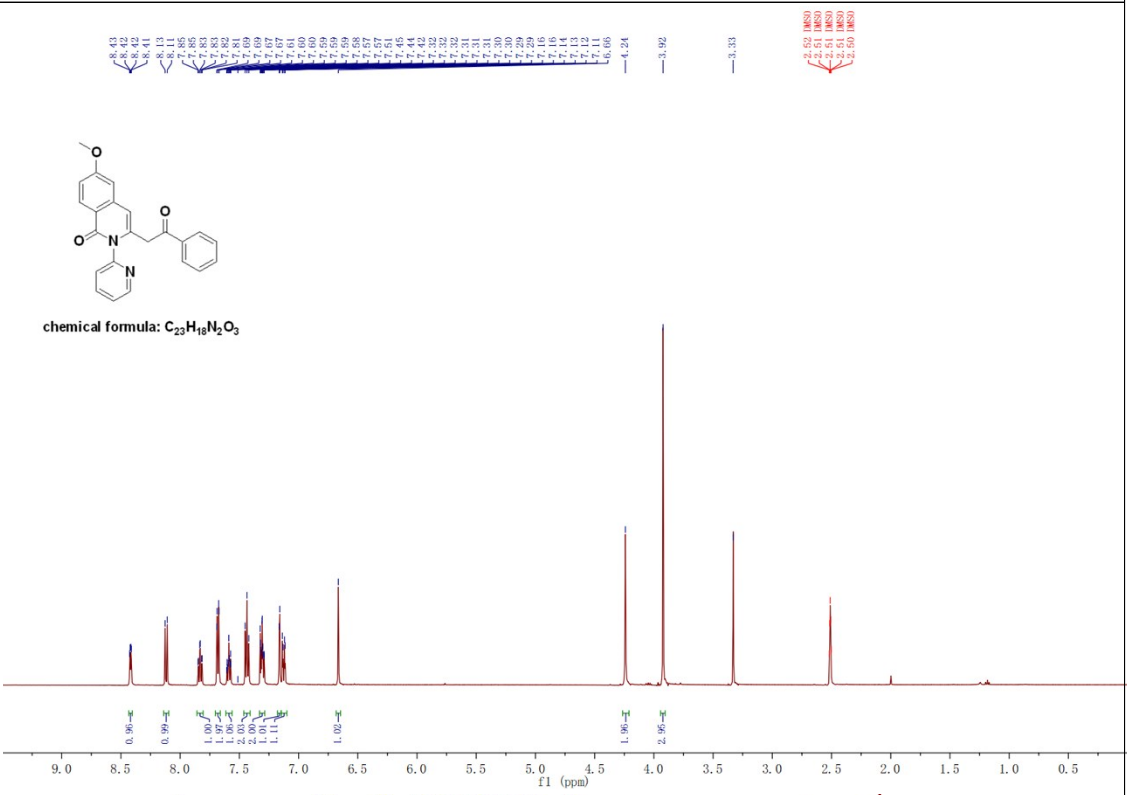

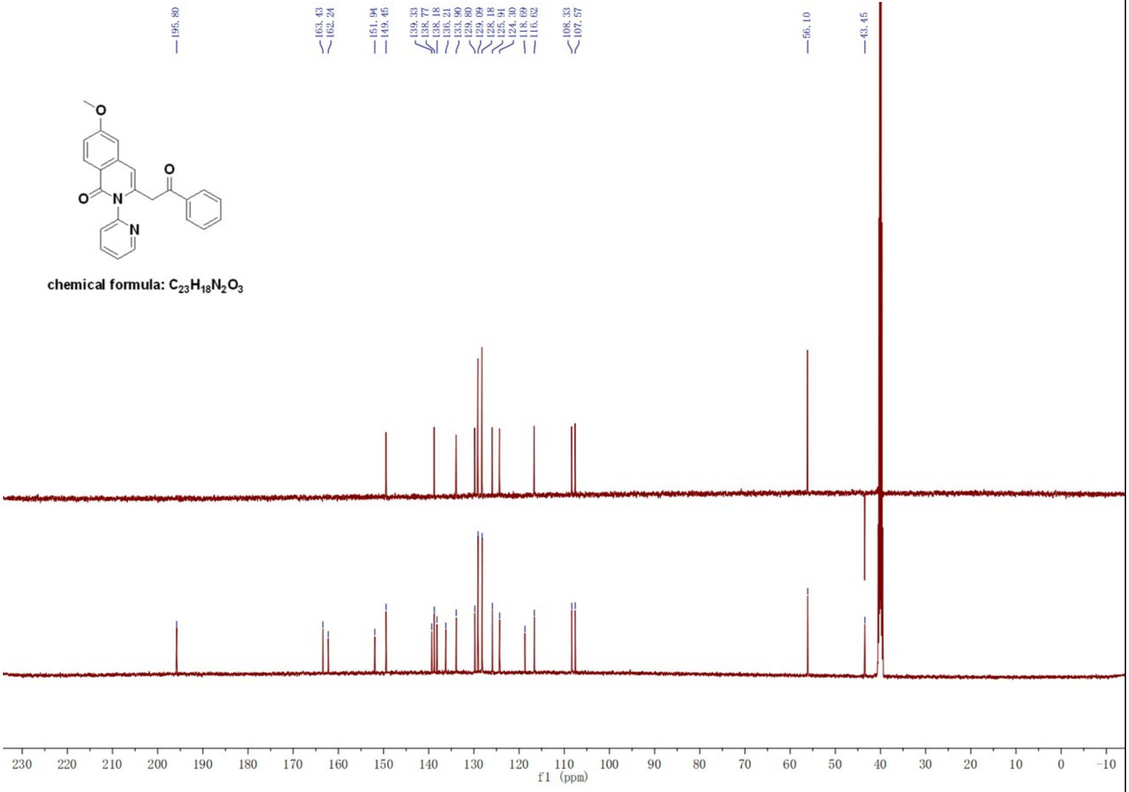

6-bromo-3-(2-oxo-2-phenylethyl)-2-(pyridin-2-yl)isoquinolin-1(2*H*)-one (3pa)

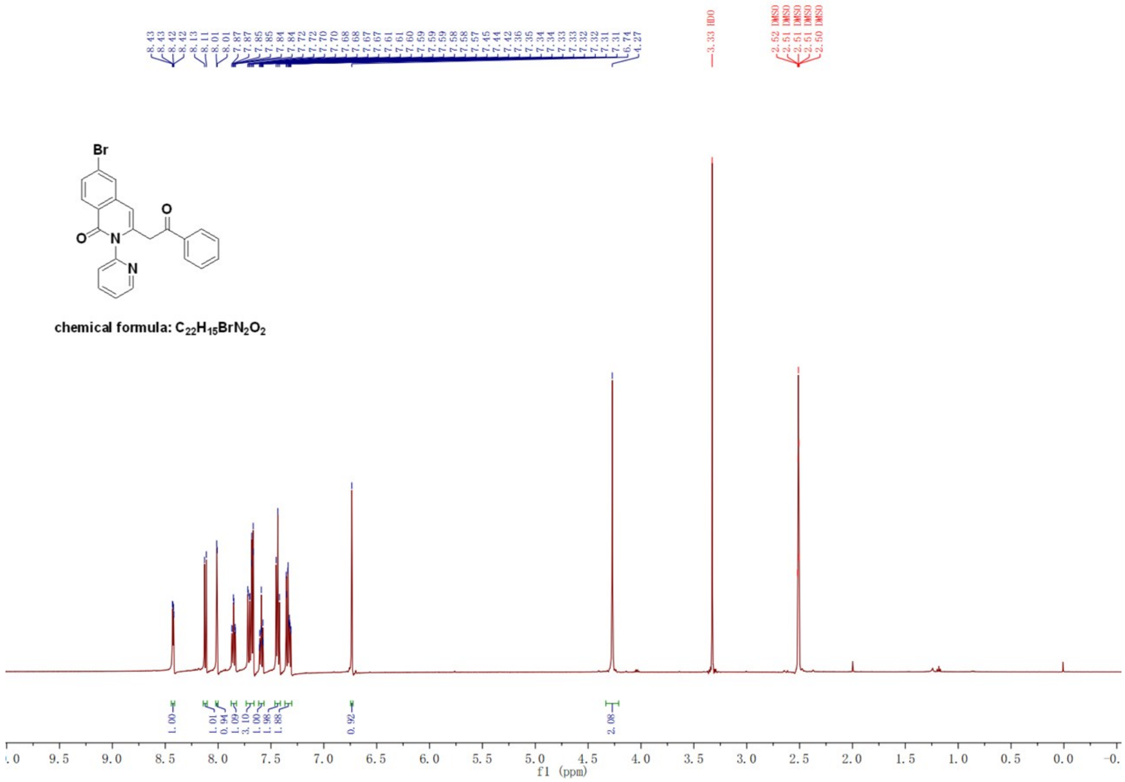

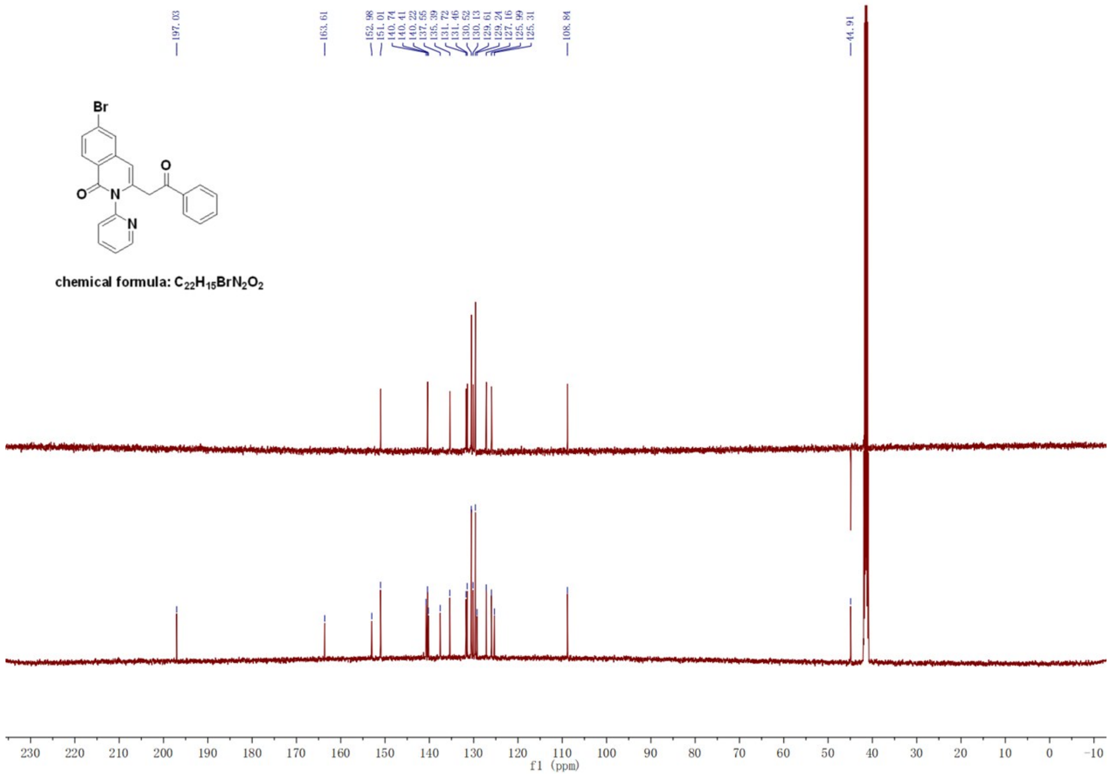

7-methyl-3-(2-oxo-2-phenylethyl)-2-(pyridin-2-yl)isoquinolin-1(2*H*)-one (3qa)

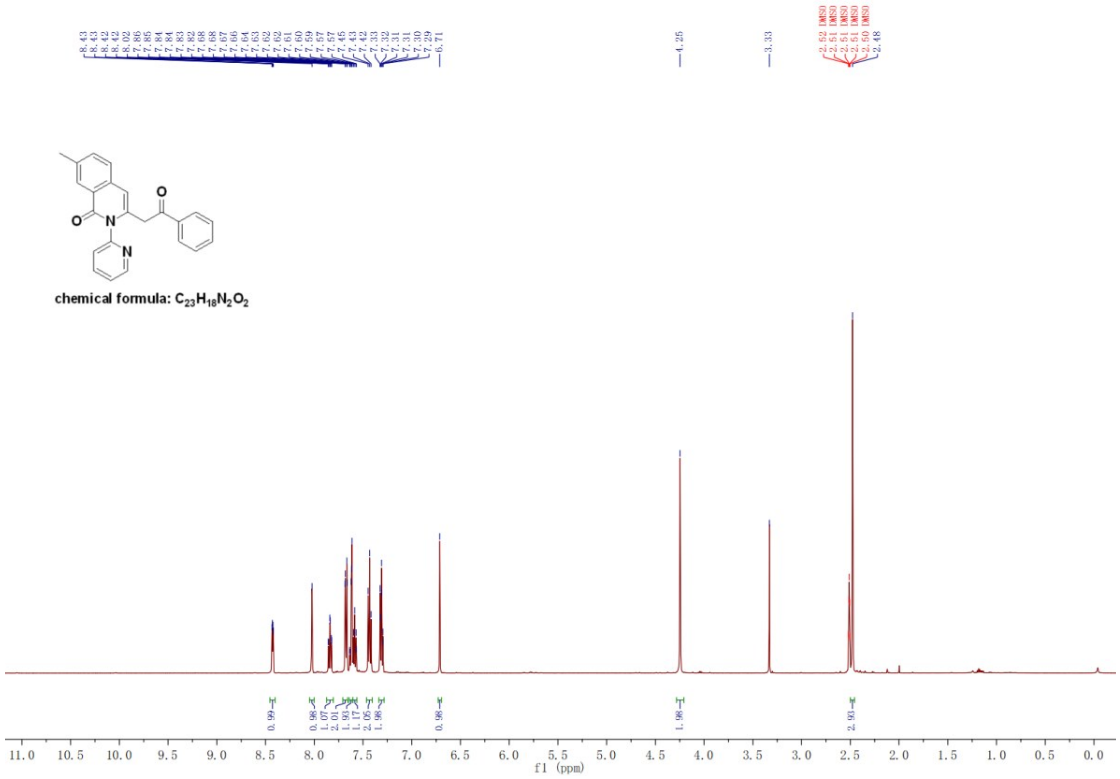

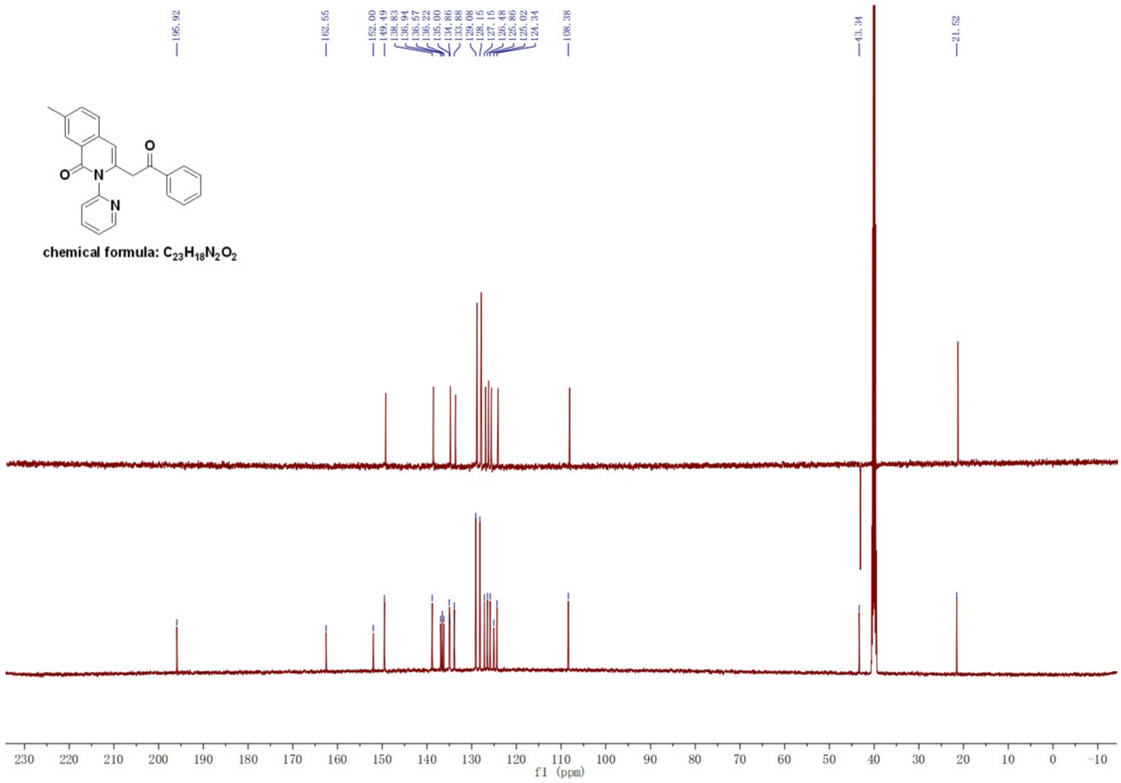

7-bromo-3-(2-oxo-2-phenylethyl)-2-(pyridin-2-yl)isoquinolin-1(2*H*)-one (3ra)

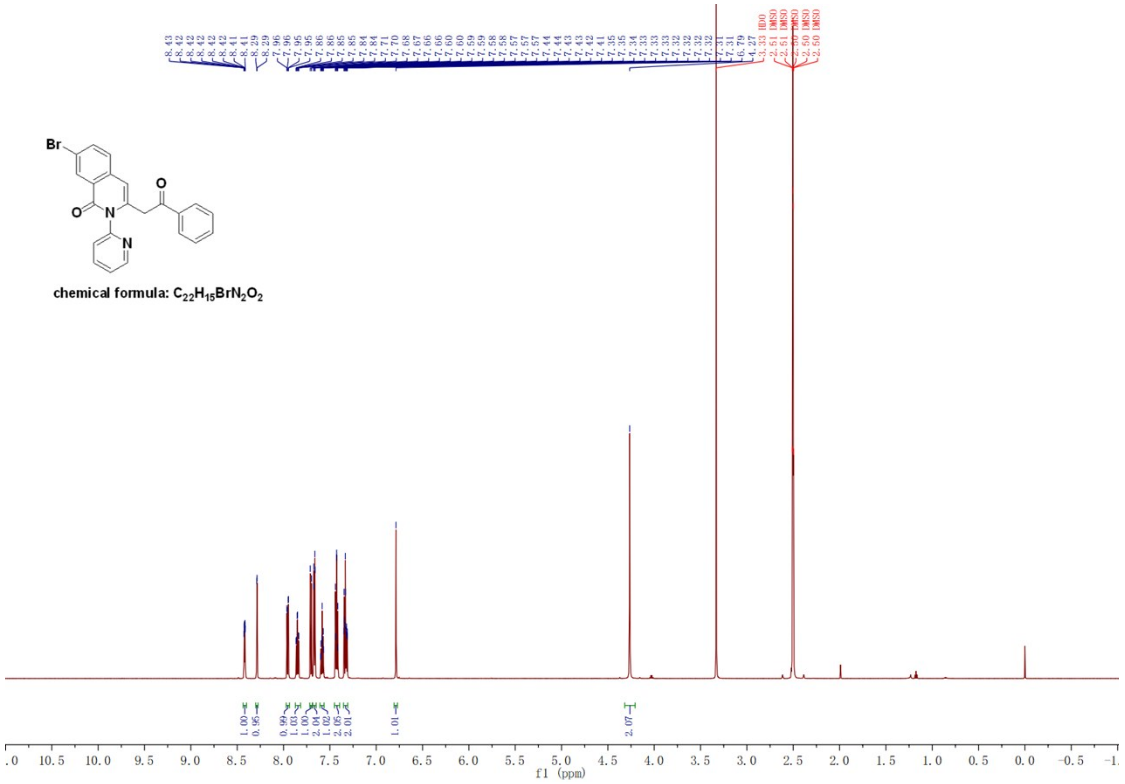

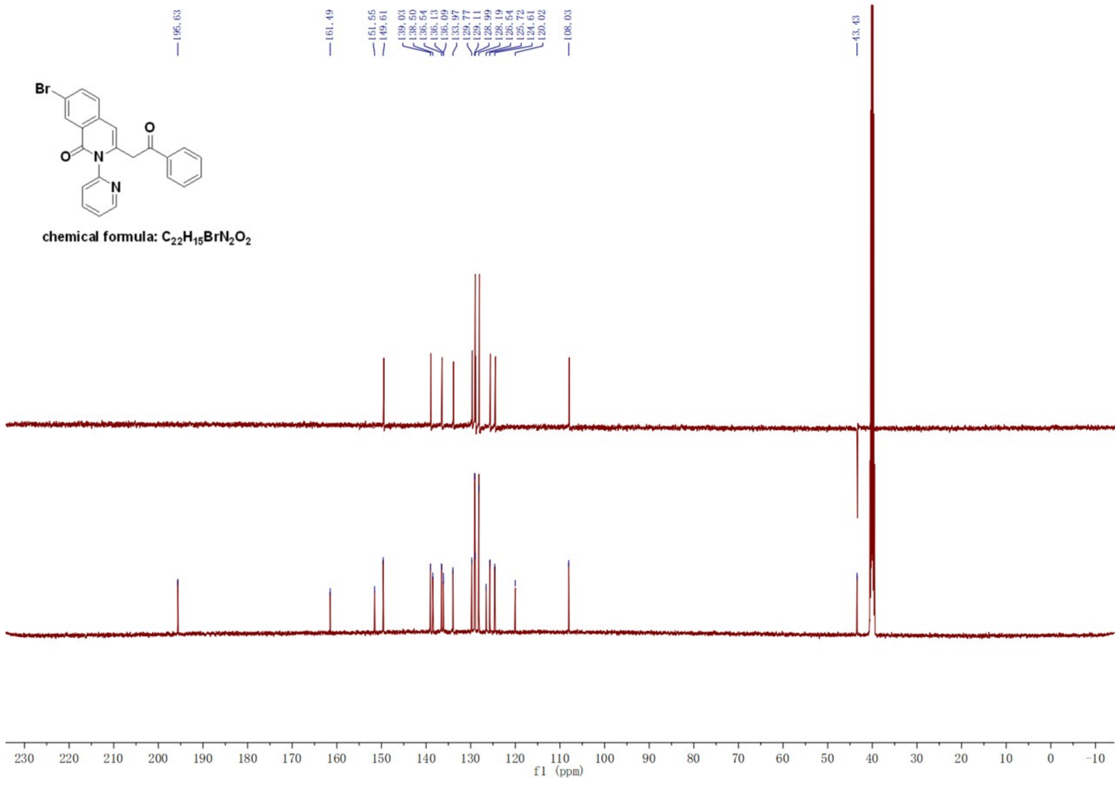

6-(2-(2-methoxyphenyl)-2-oxoethyl)-2*H*-[1,2'-bipyridin]-2-one (3ab)

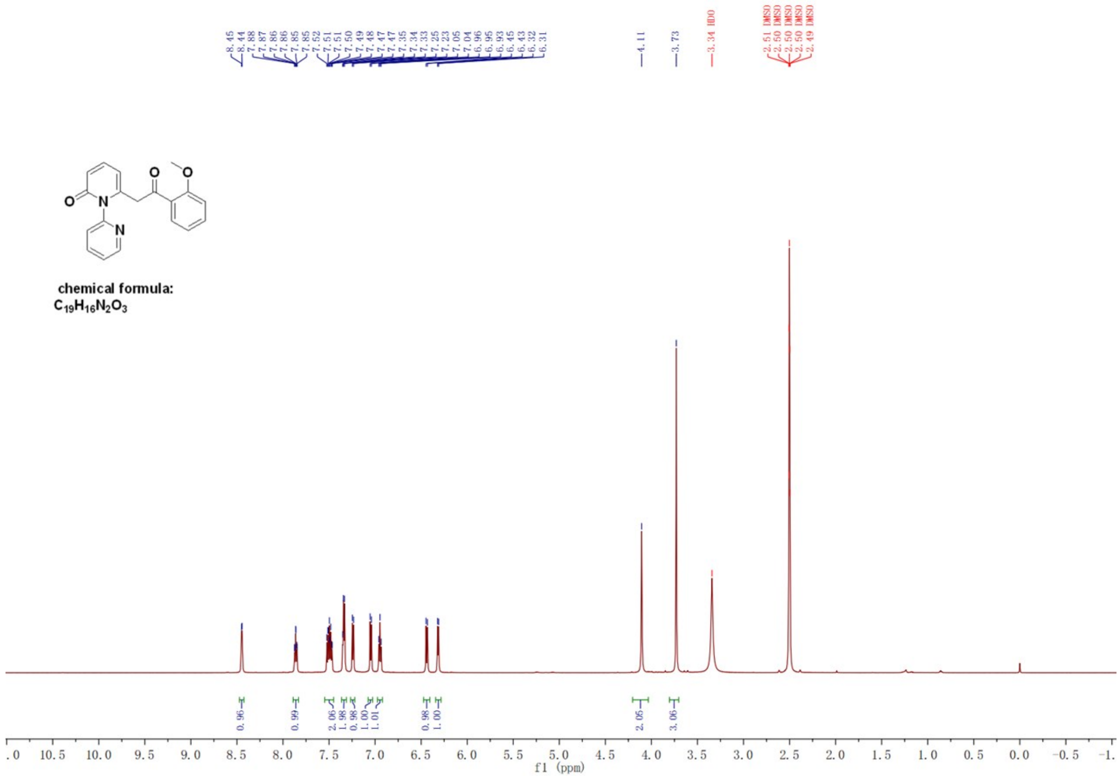

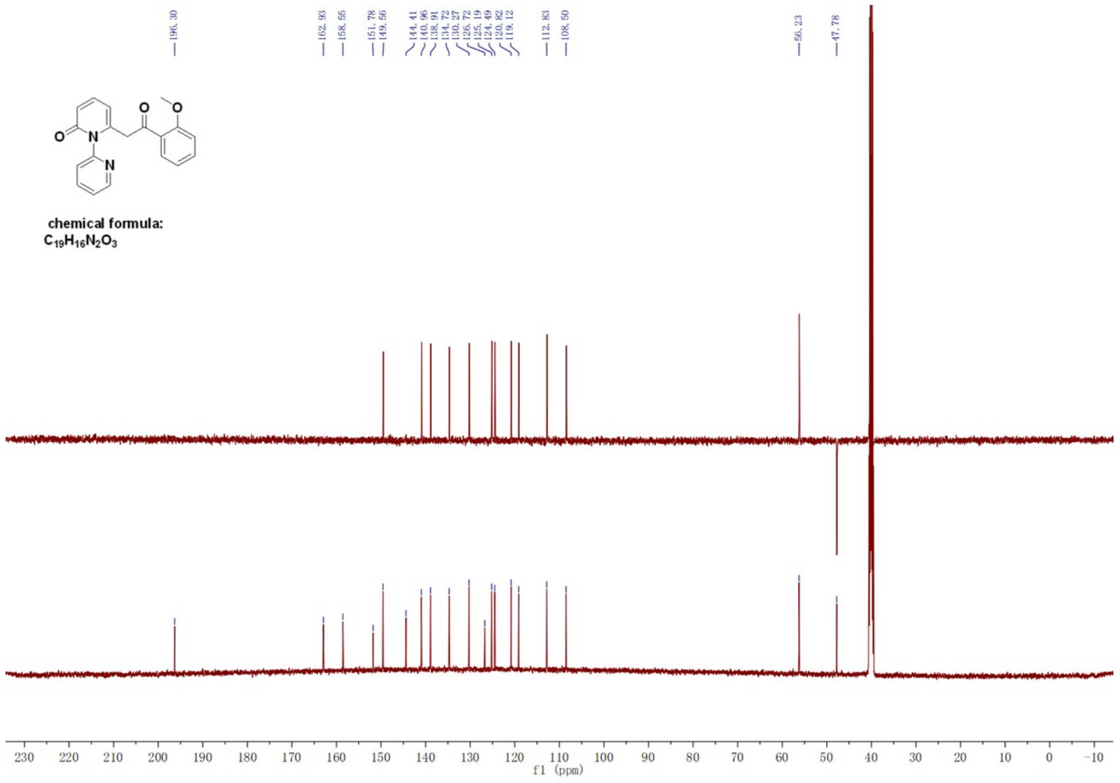

6-(2-oxo-2-(o-tolyl)ethyl)-2*H*-[1,2'-bipyridin]-2-one (3ac)

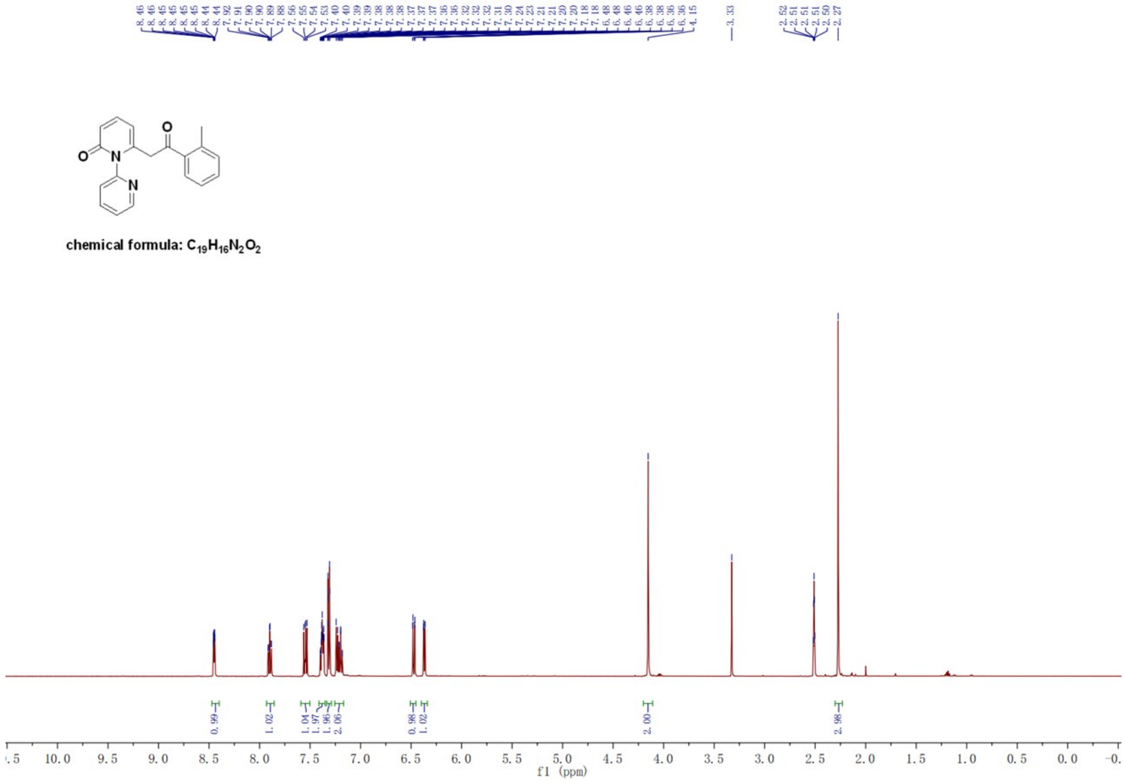

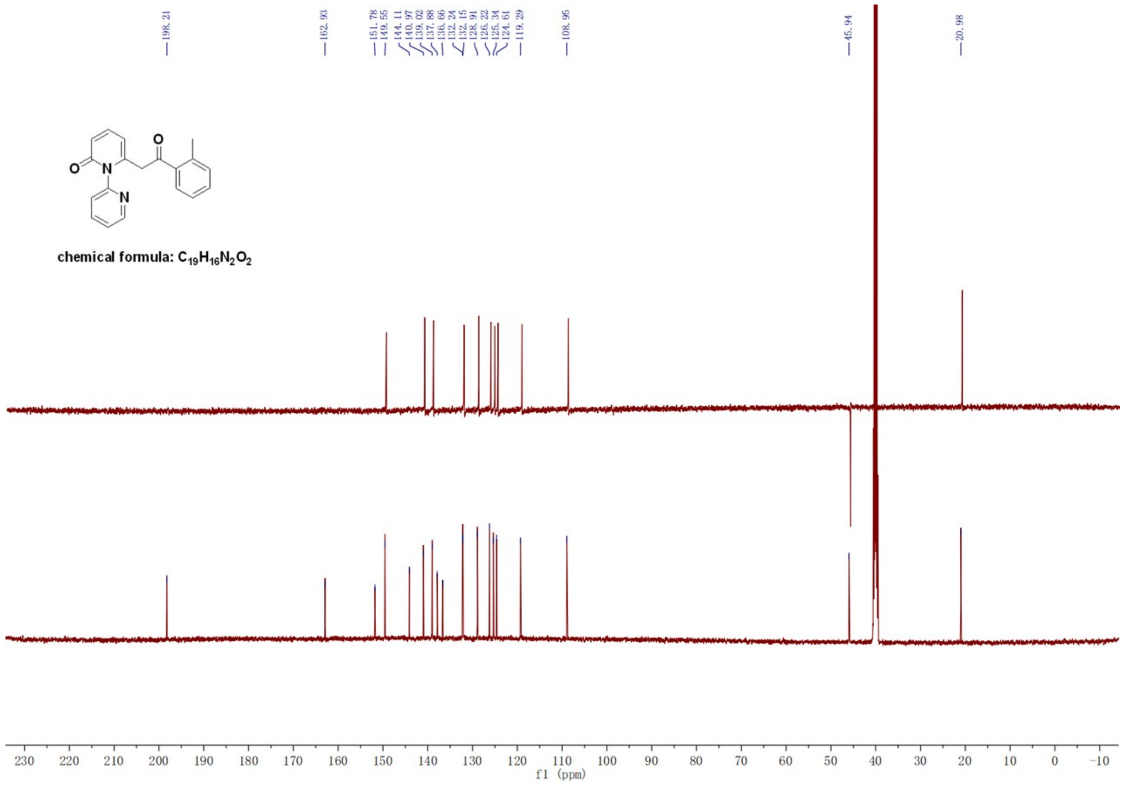

6-(2-(2-fluorophenyl)-2-oxoethyl)-2*H*-[1,2'-bipyridin]-2-one (3ad)

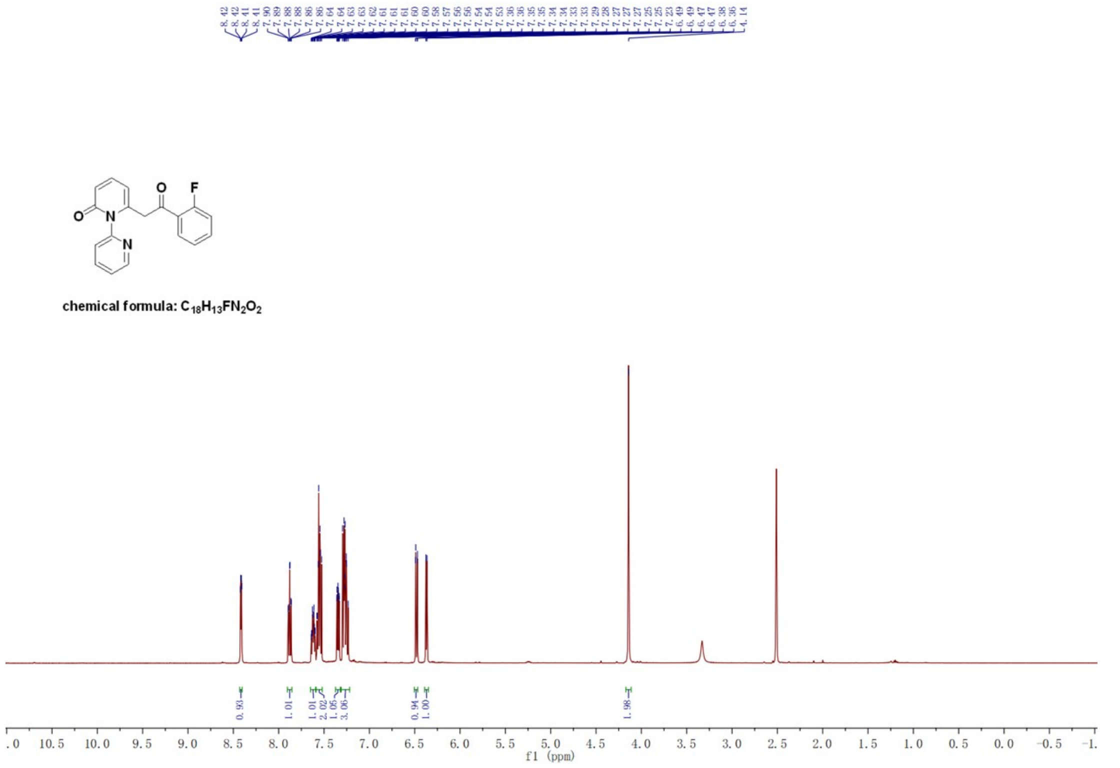

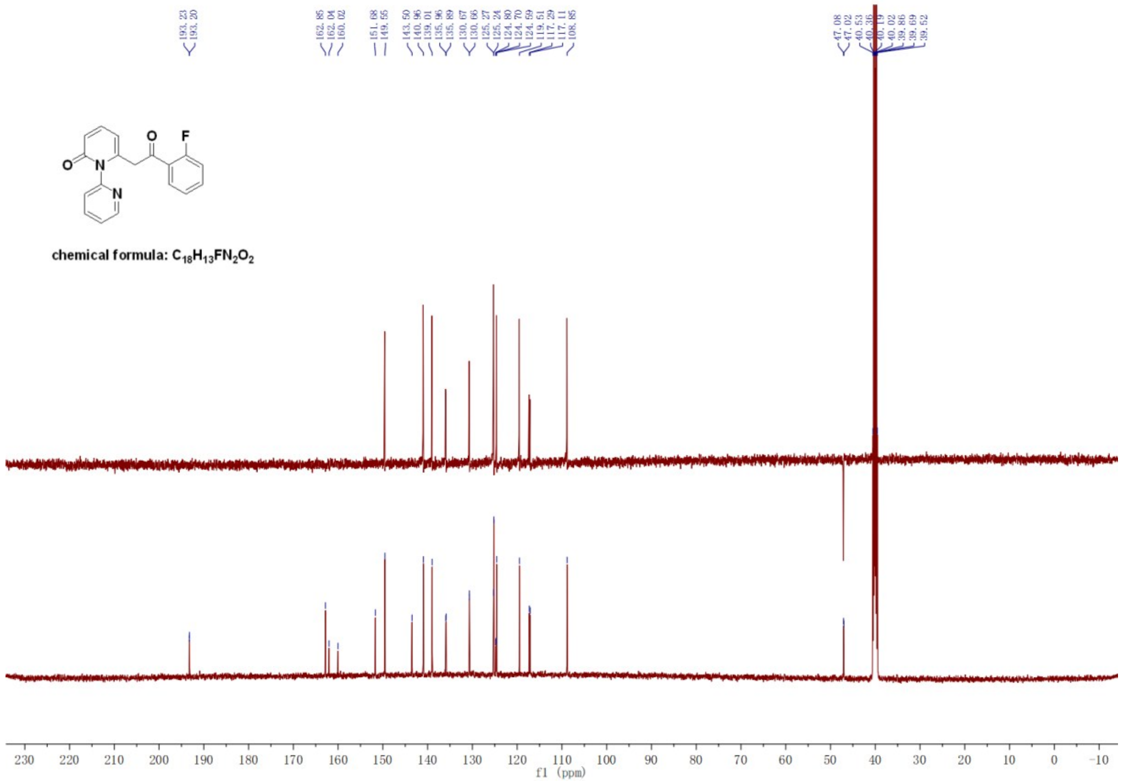

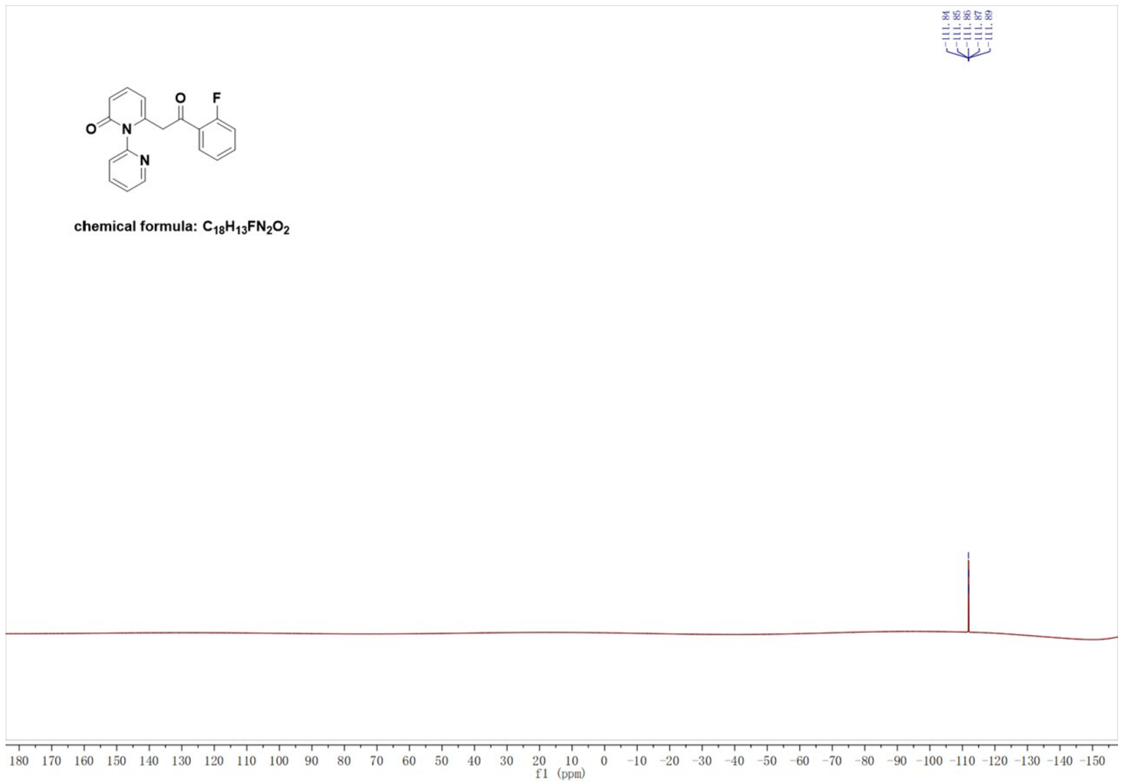

6-(2-(2-chlorophenyl)-2-oxoethyl)-2*H*-[1,2'-bipyridin]-2-one (3ae)

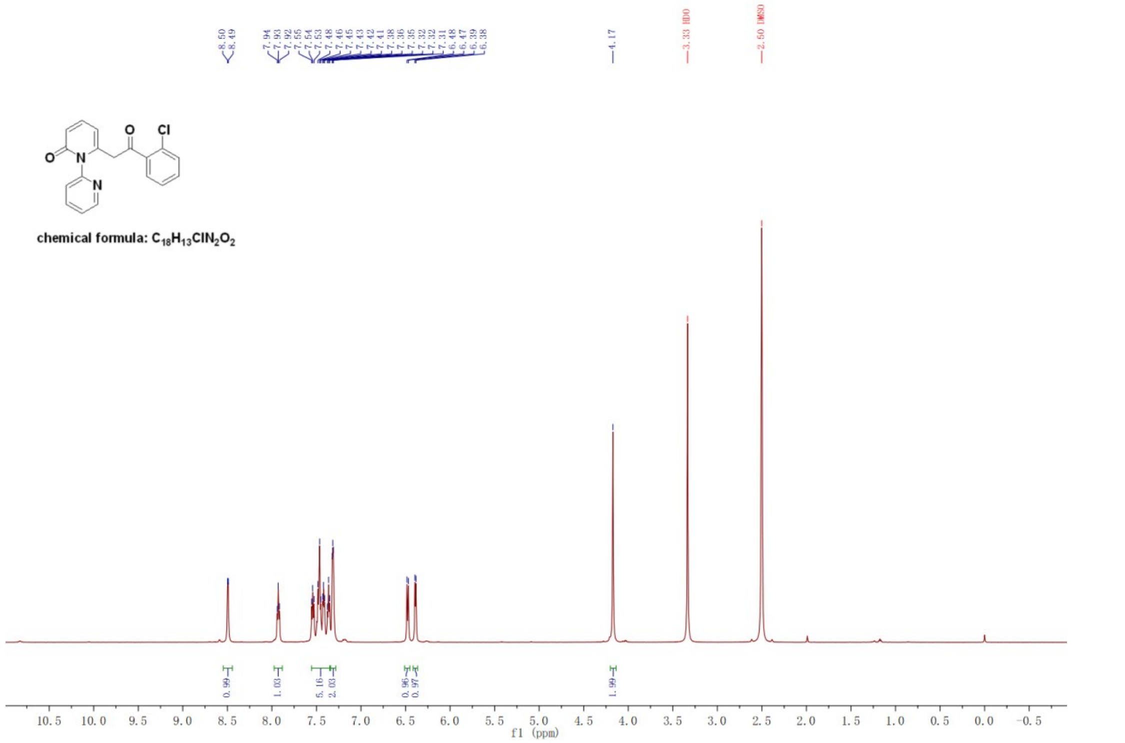

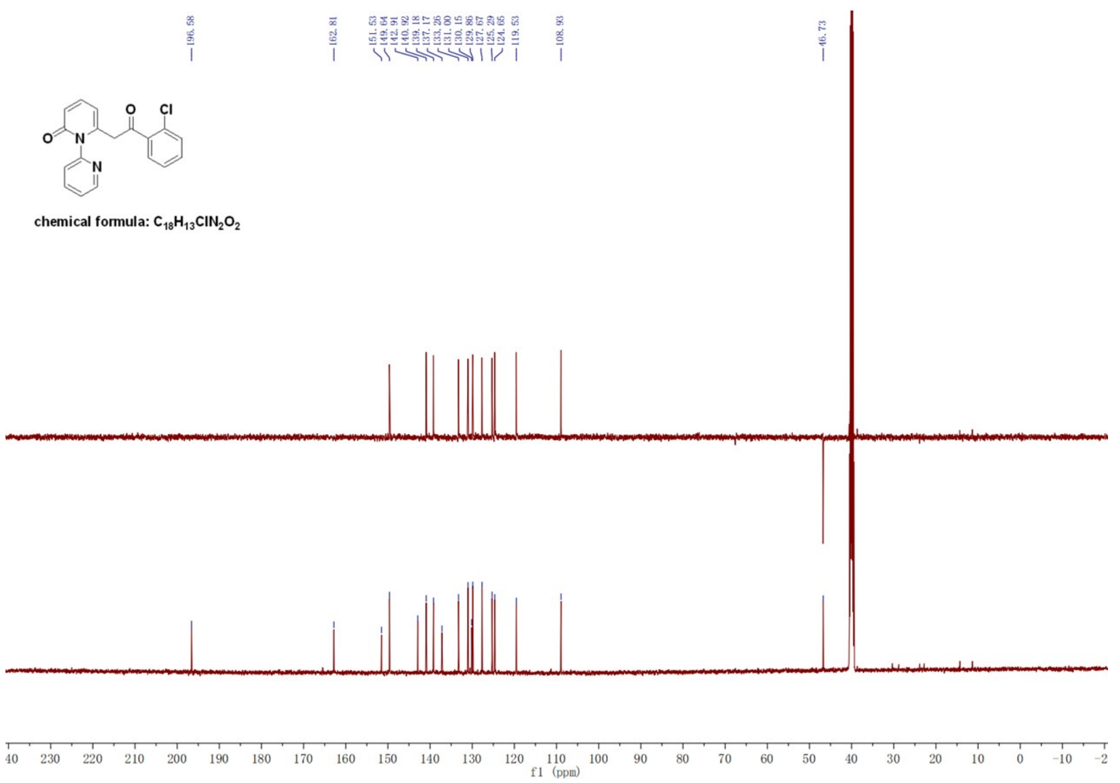

6-(2-oxo-2-(m-tolyl)ethyl)-2*H*-[1,2'-bipyridin]-2-one (3af)

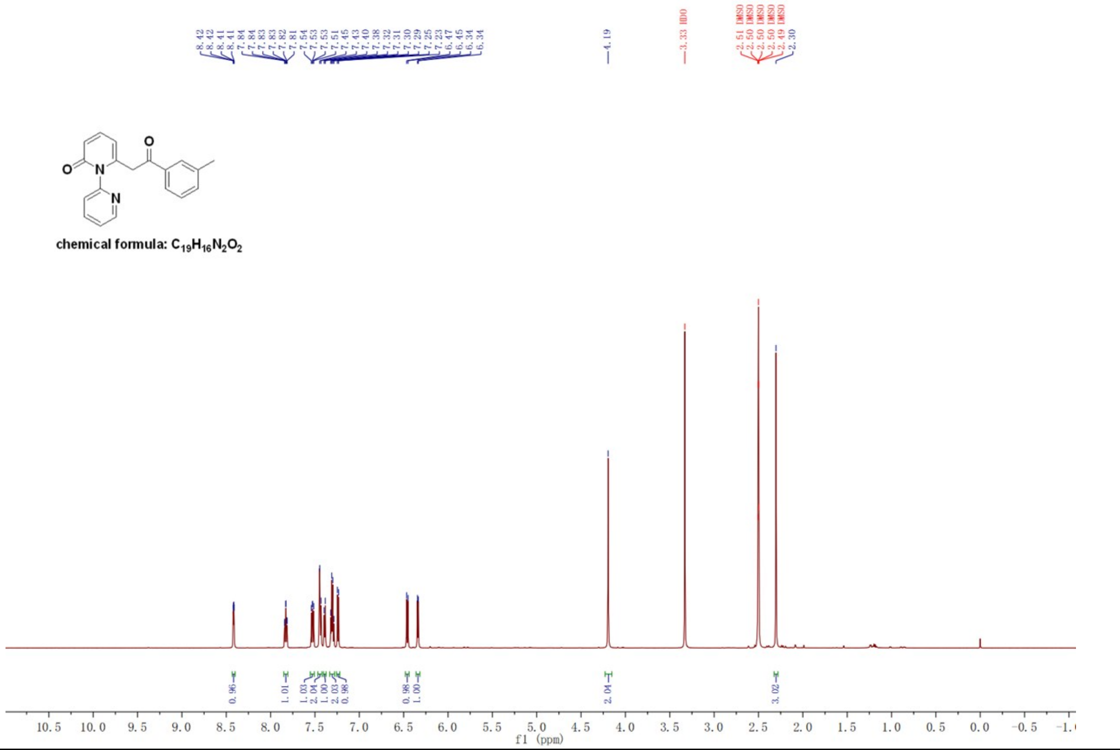

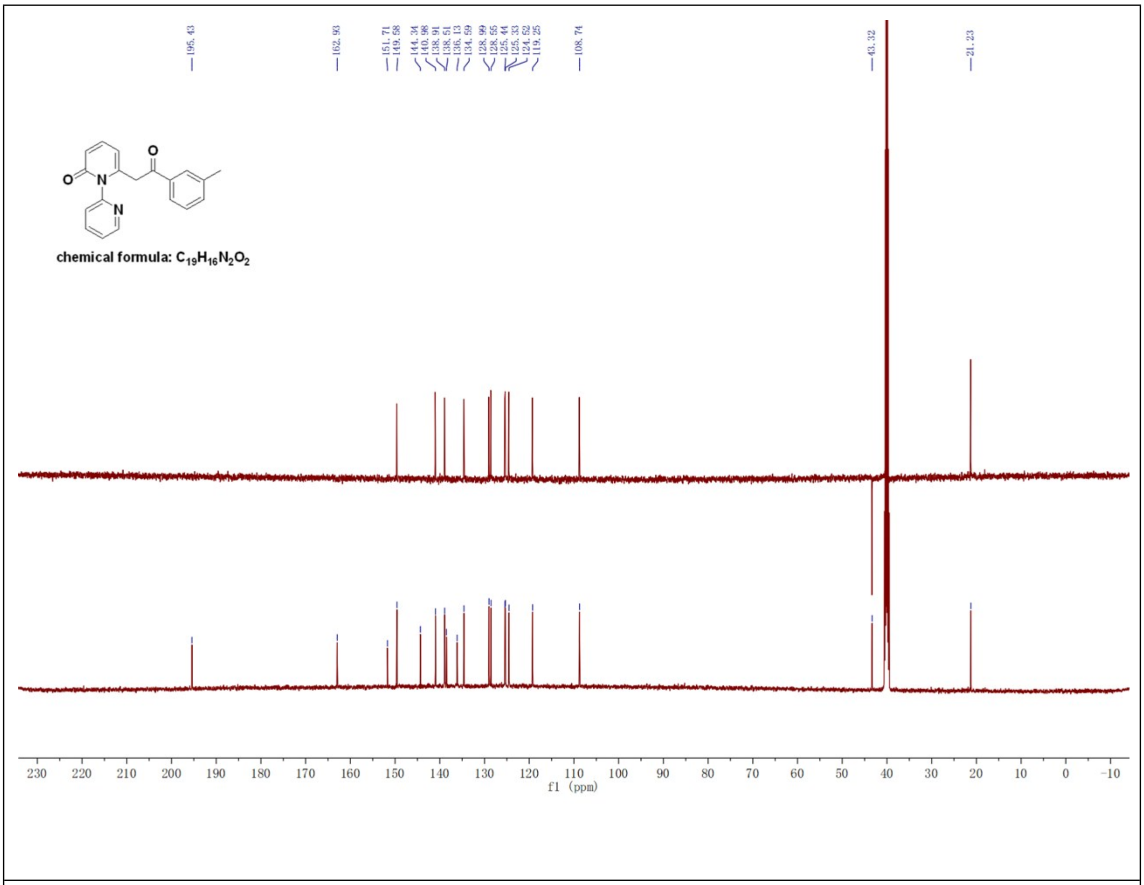

6-(2-(3-bromophenyl)-2-oxoethyl)-2*H*-[1,2'-bipyridin]-2-one (3ag)

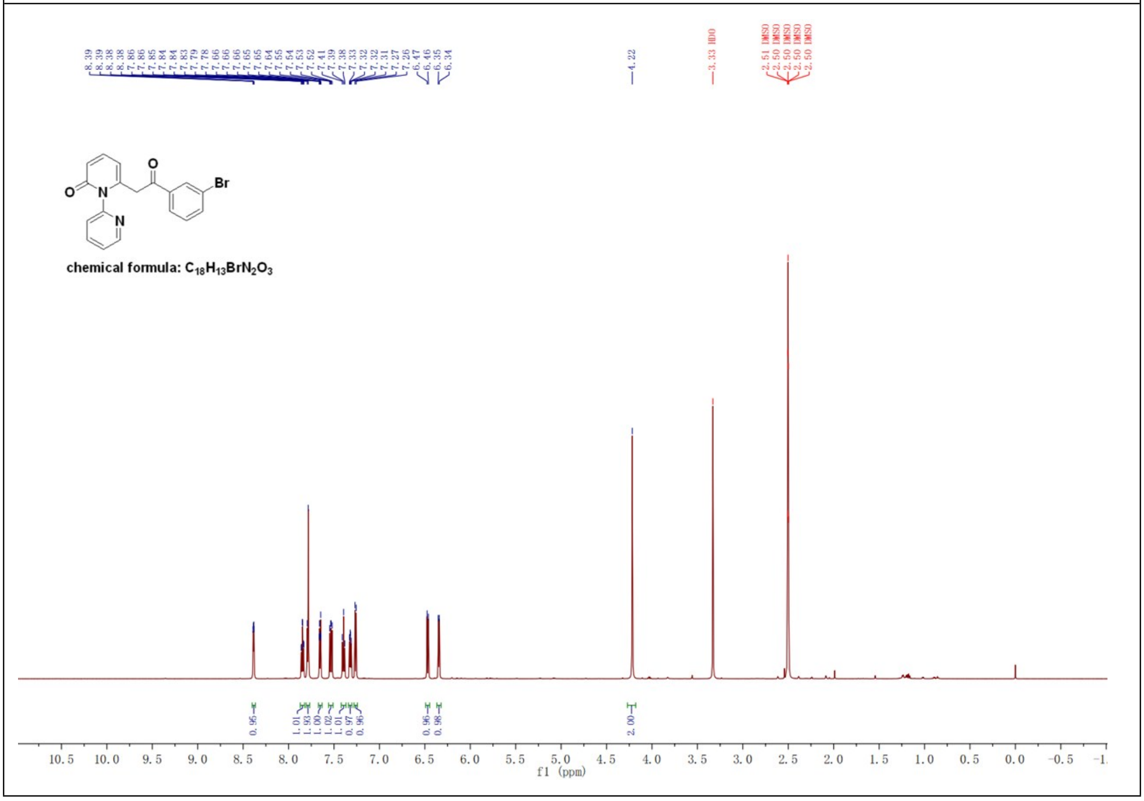

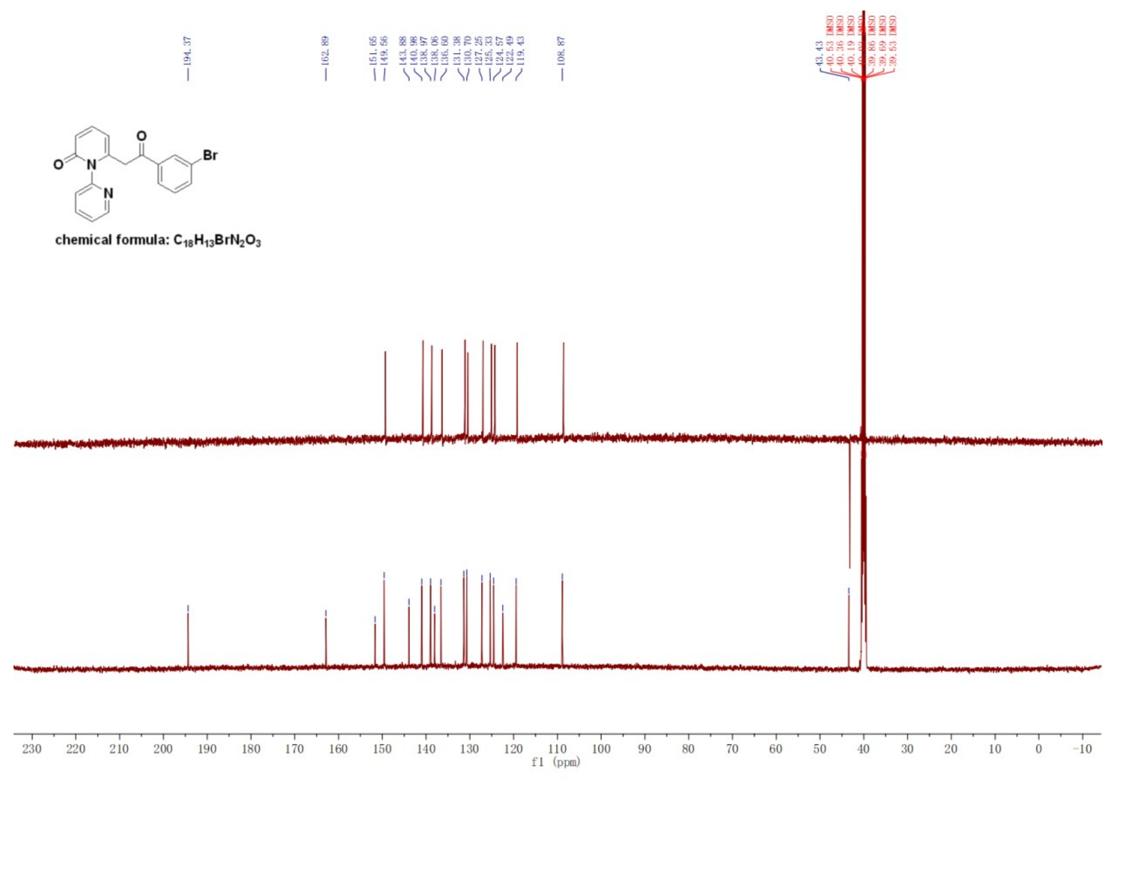

6-(2-(4-methoxyphenyl)-2-oxoethyl)-2*H*-[1,2'-bipyridin]-2-one (3ah)

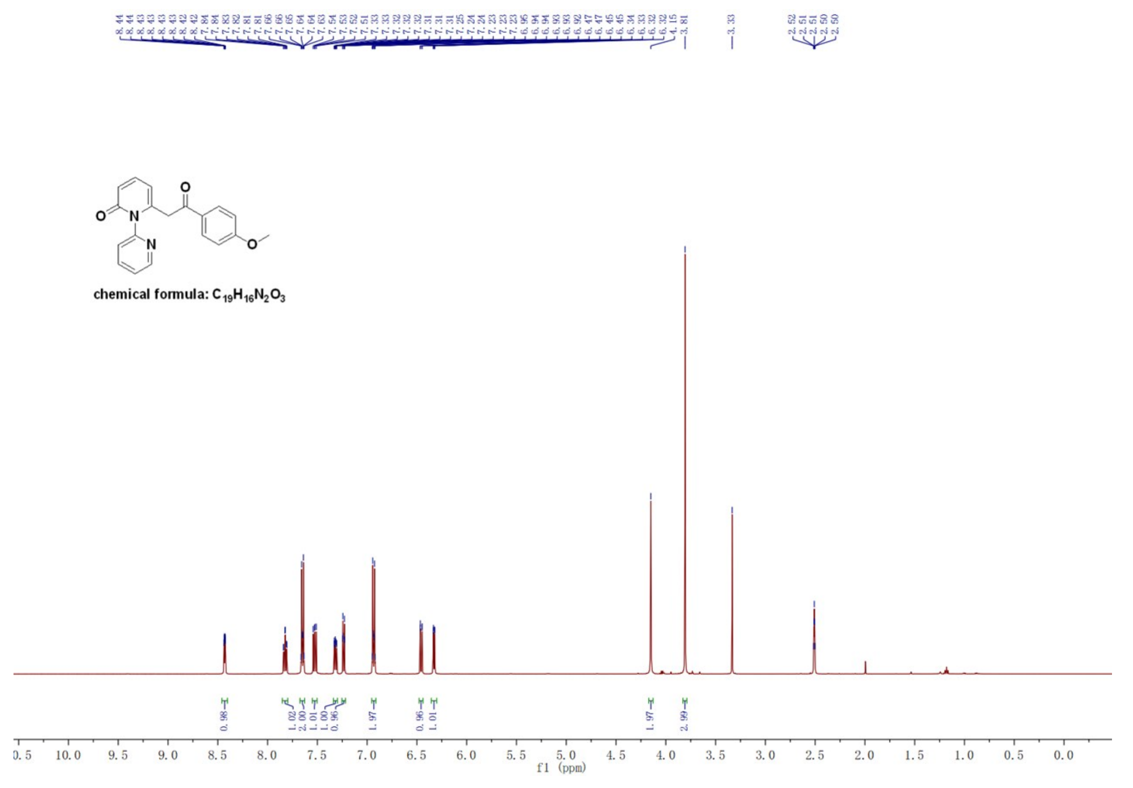

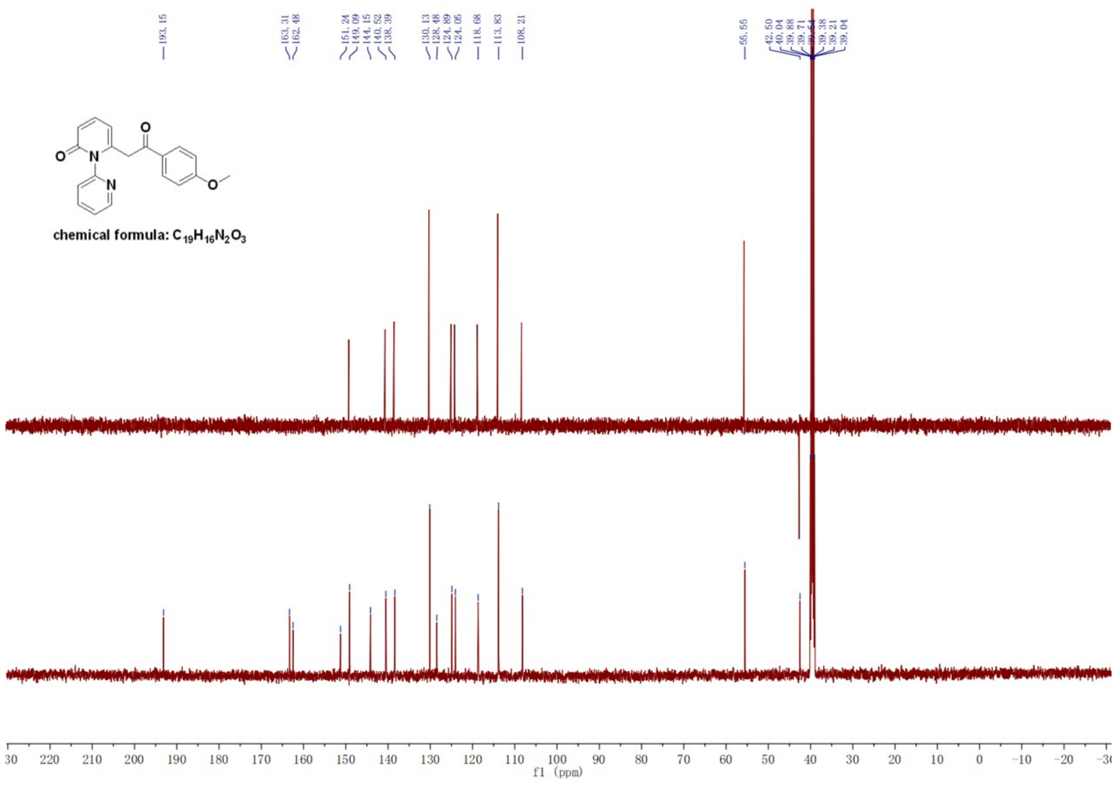

6-(2-oxo-2-(p-tolyl)ethyl)-2*H*-[1,2'-bipyridin]-2-one (3ai)

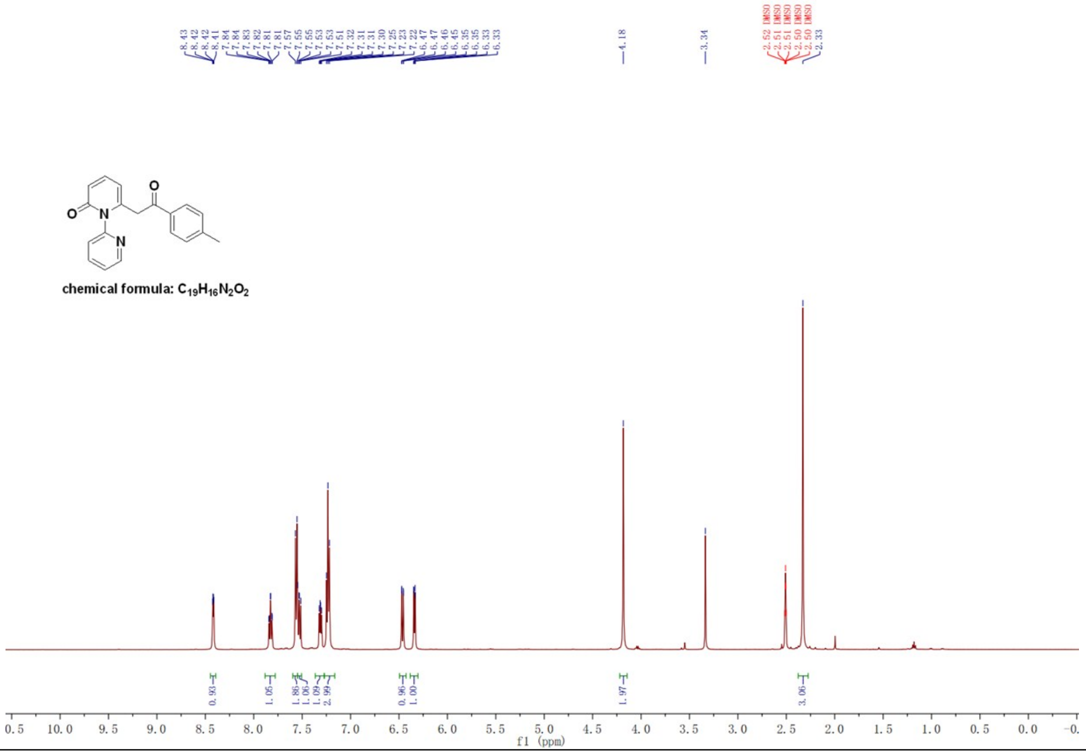

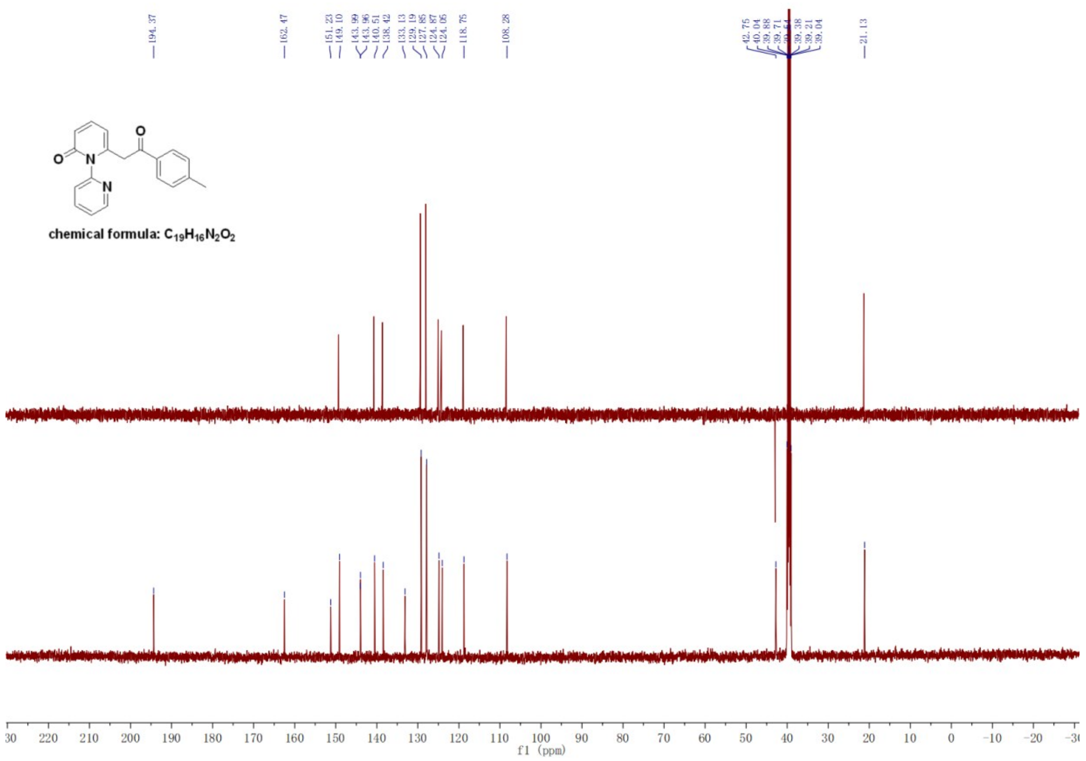

6-(2-([1,1'-biphenyl]-4-yl)-2-oxoethyl)-2*H*-[1,2'-bipyridin]-2-one (3aj)

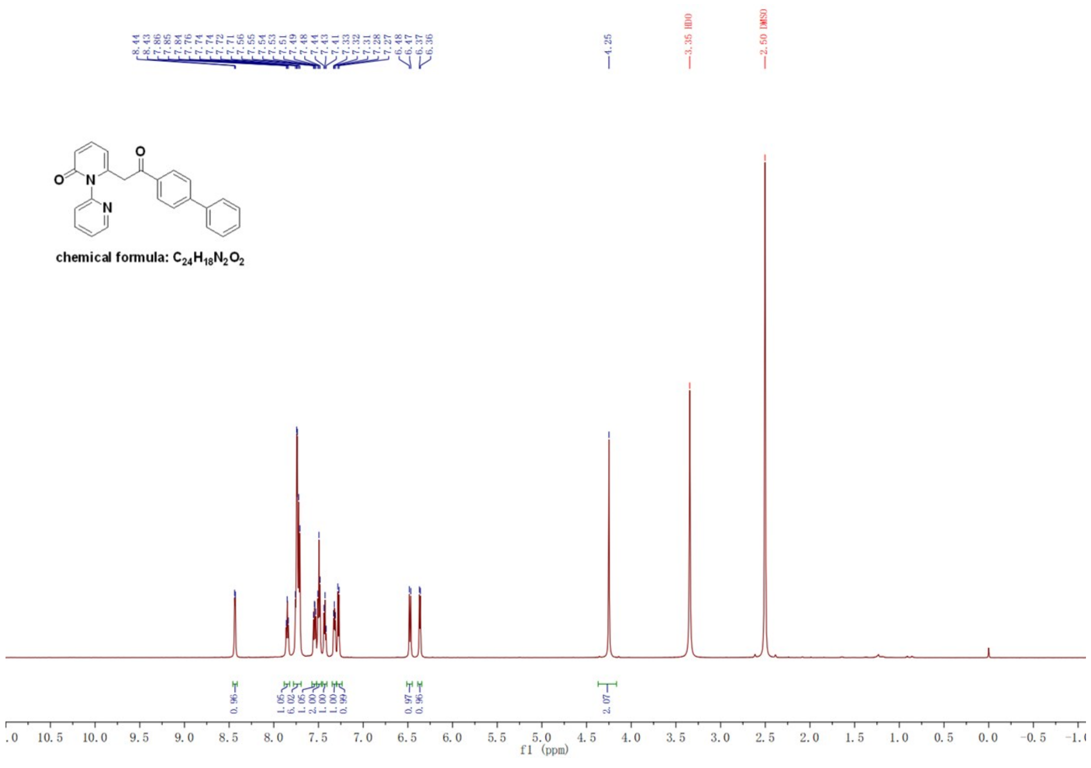

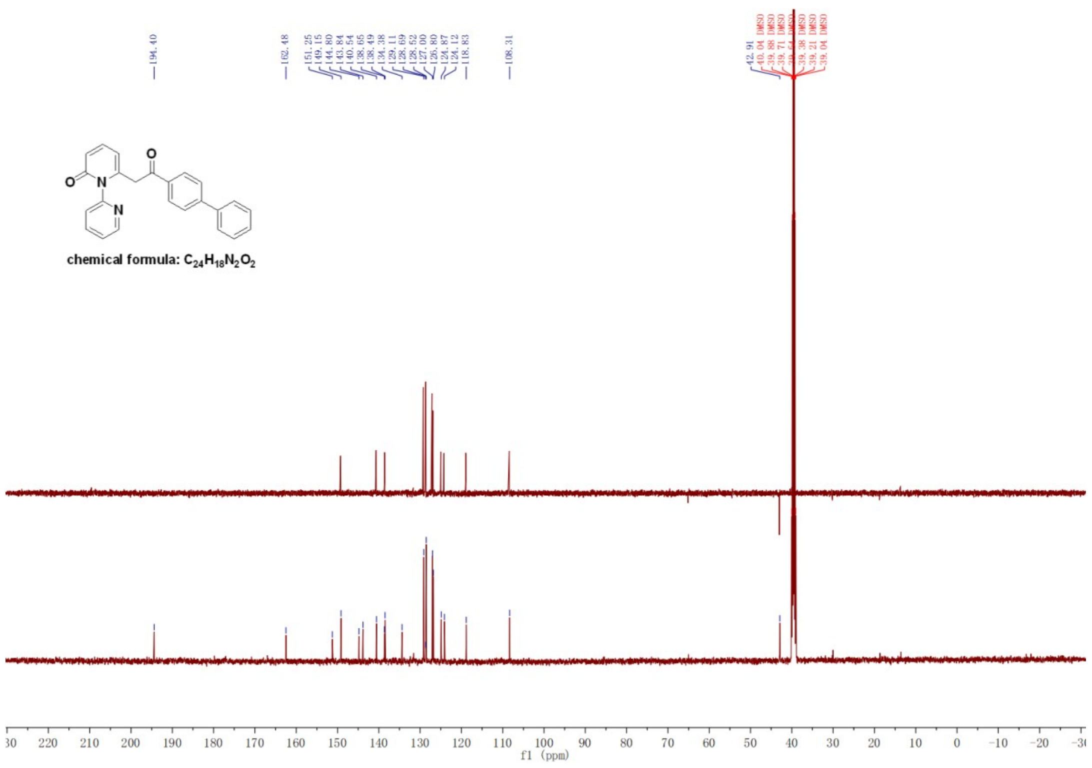

6-(2-(4-fluorophenyl)-2-oxoethyl)-2*H*-[1,2'-bipyridin]-2-one (3ak)

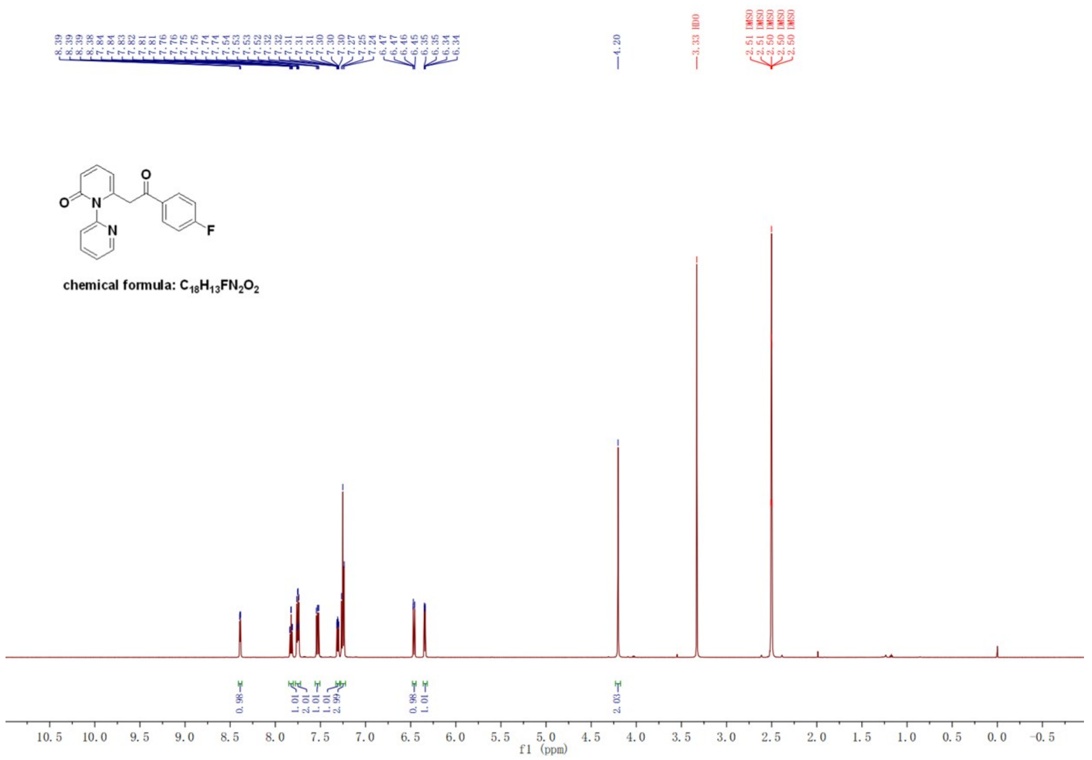

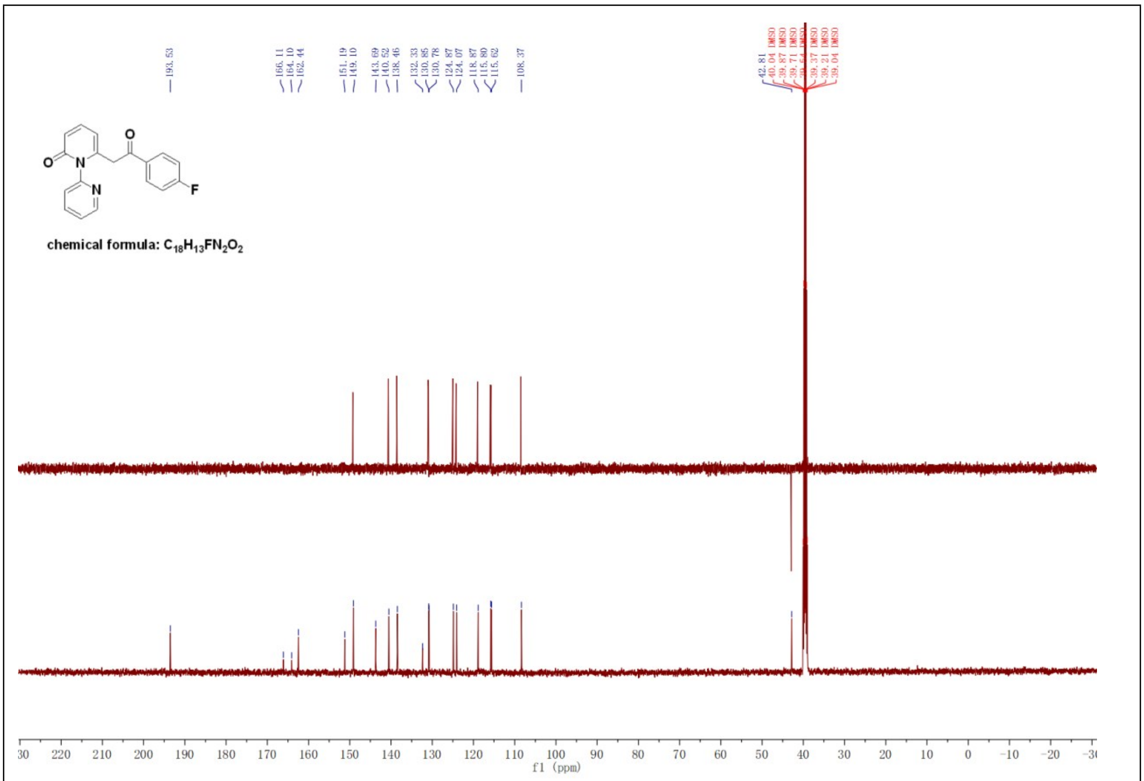

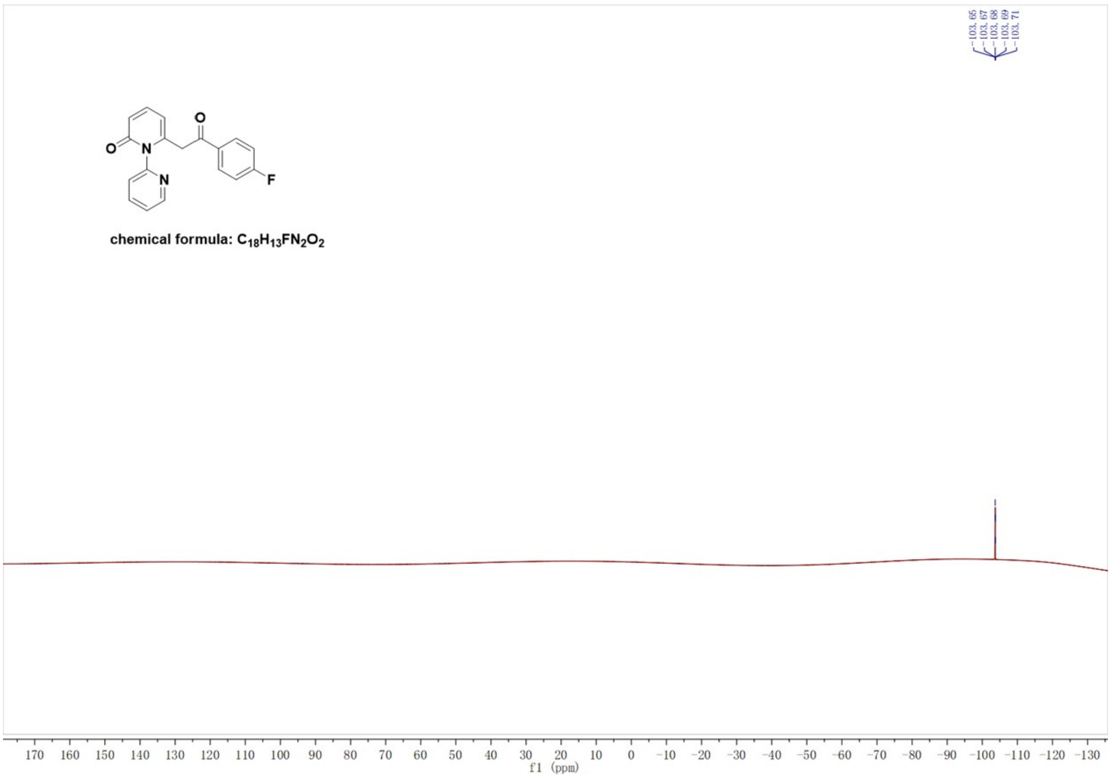

6-(2-(4-chlorophenyl)-2-oxoethyl)-2*H*-[1,2'-bipyridin]-2-one (3al)

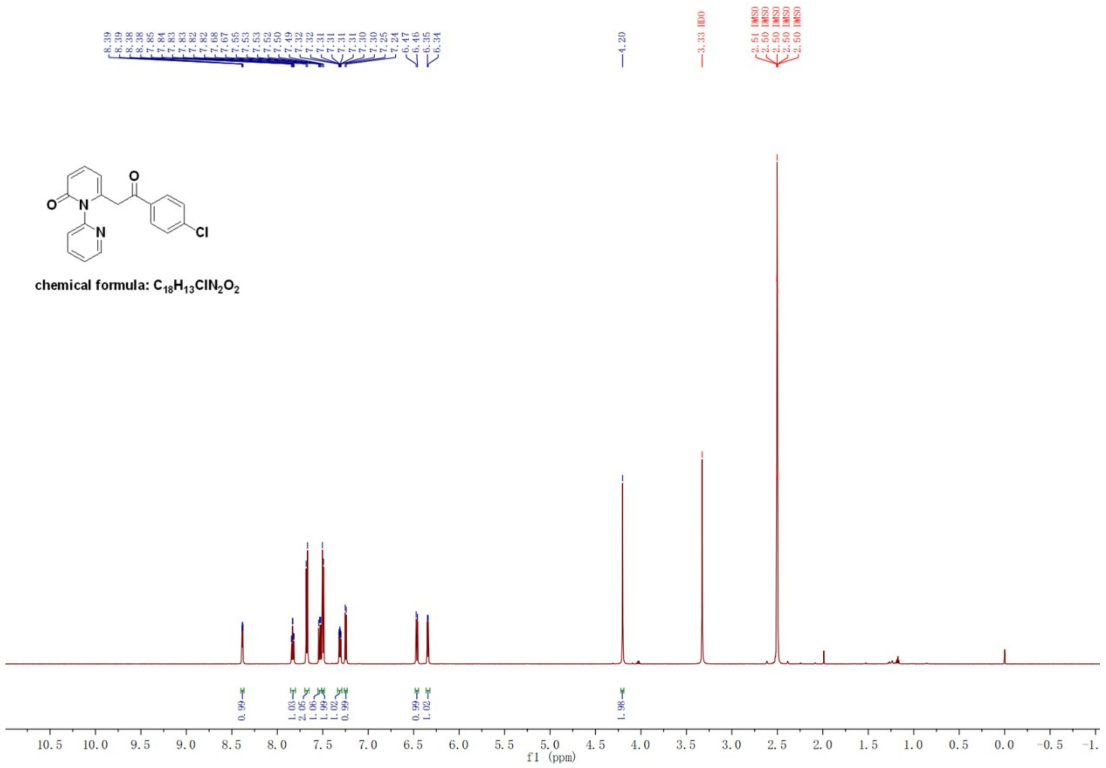

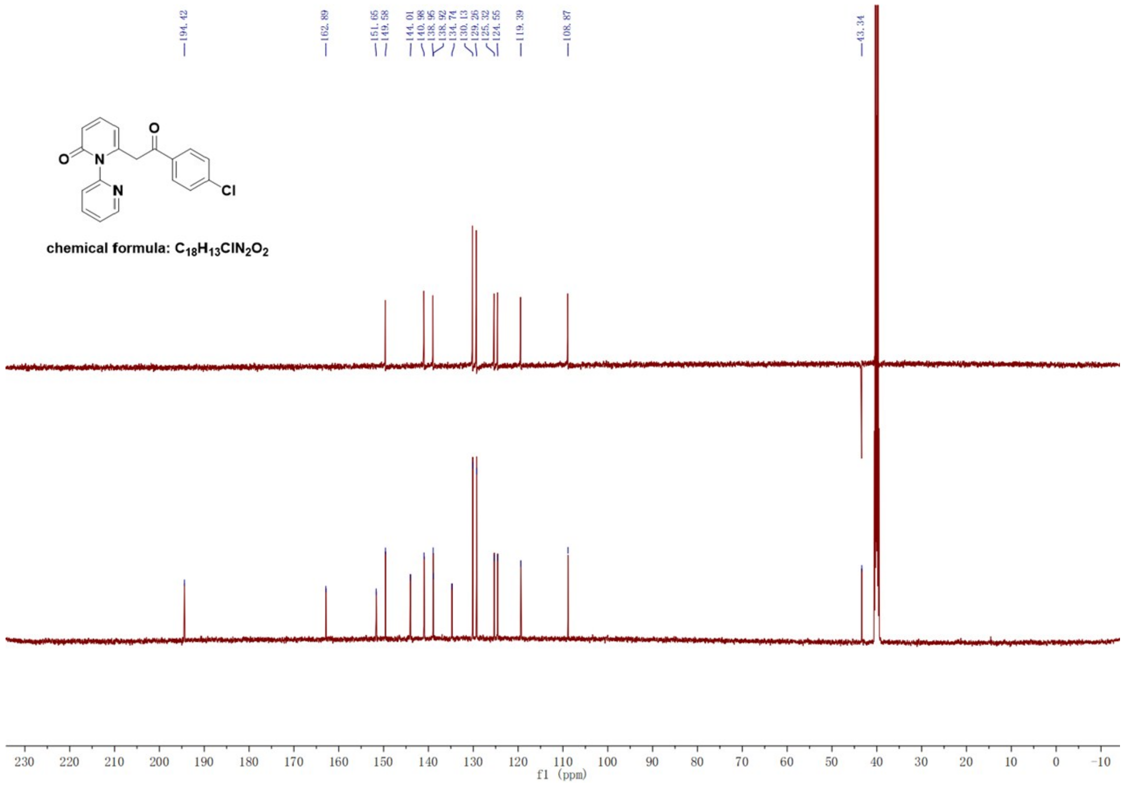

6-(2-(4-bromophenyl)-2-oxoethyl)-2*H*-[1,2'-bipyridin]-2-one (3am)

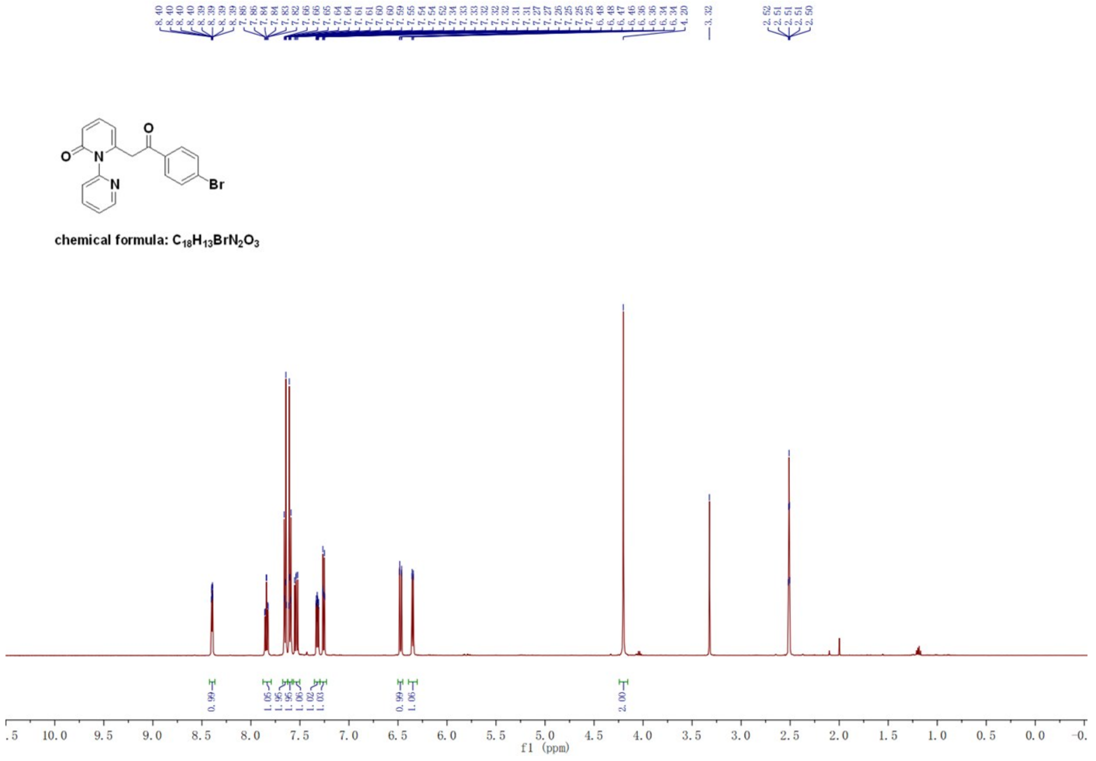

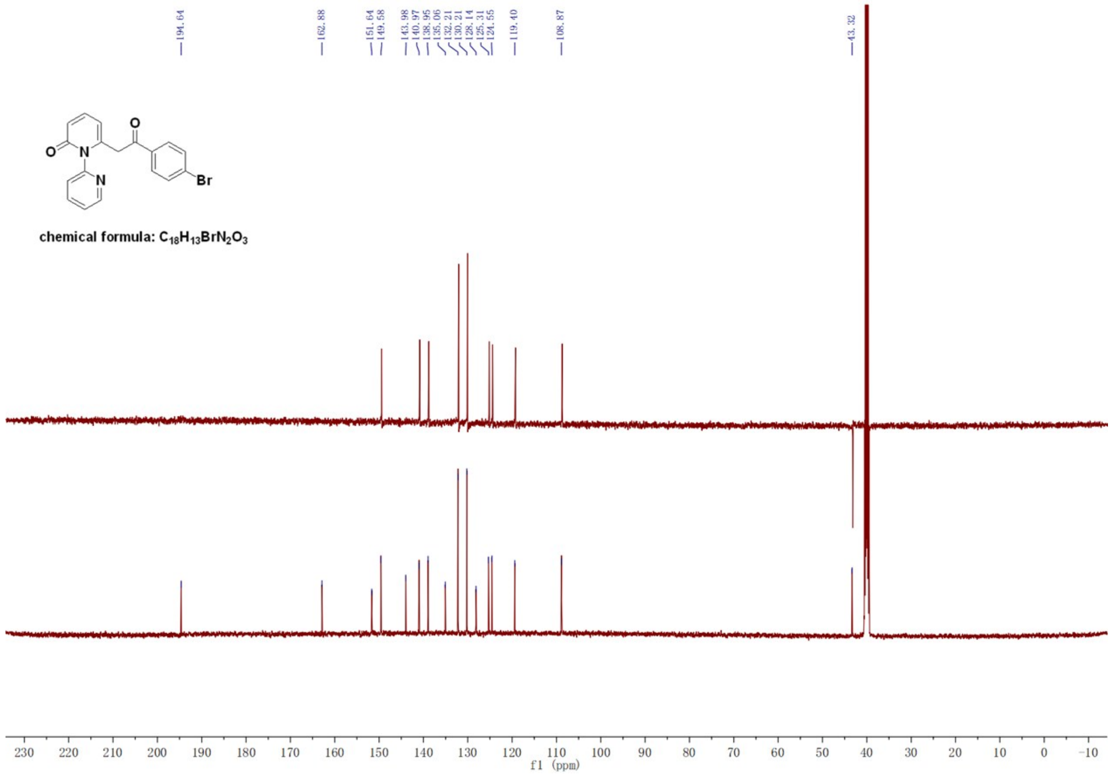

6-(2-oxo-2-(4-(trifluoromethyl)phenyl)ethyl)-2*H*-[1,2'-bipyridin]-2-one (3an)

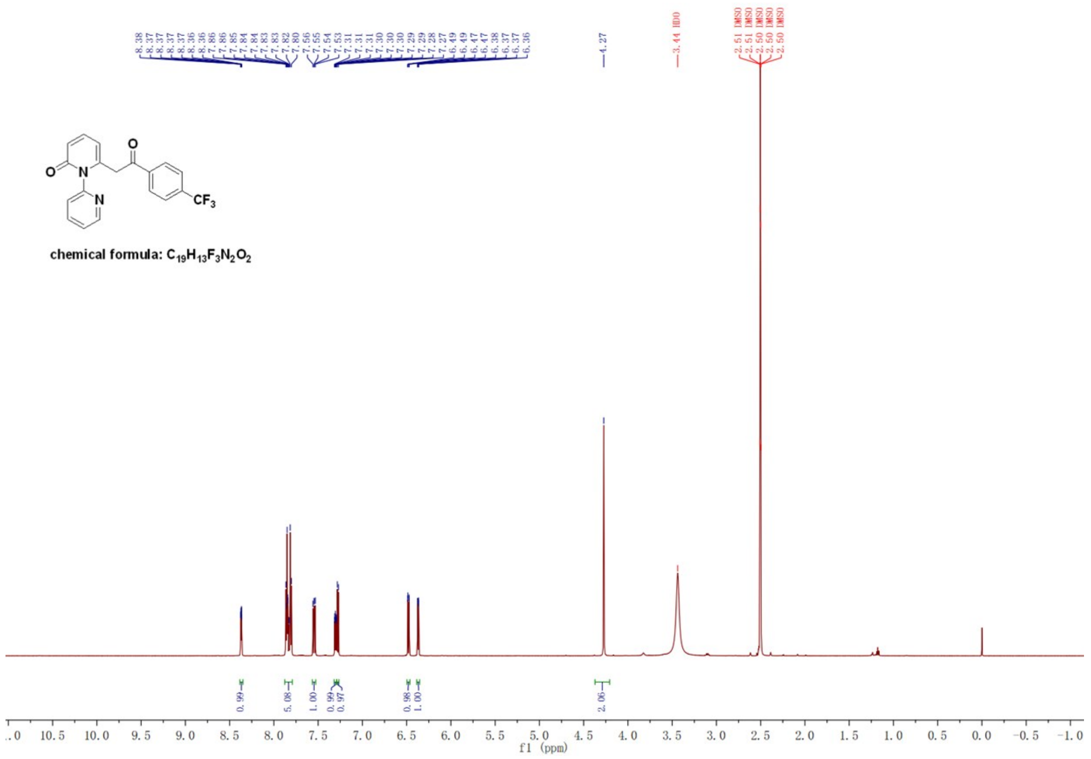

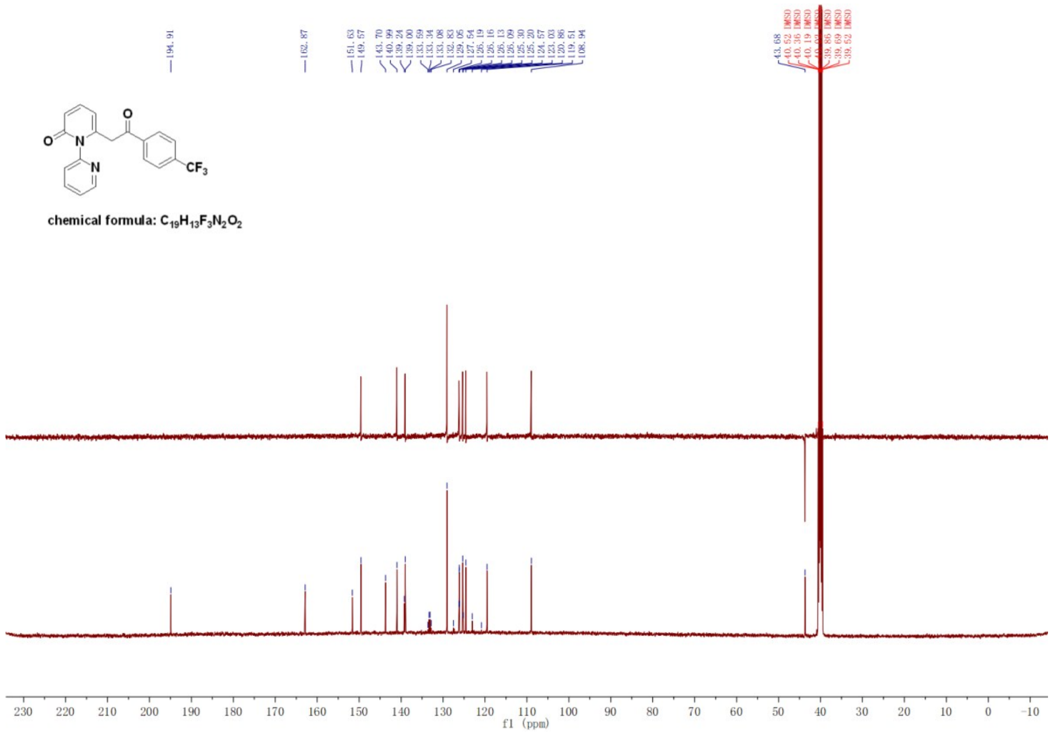

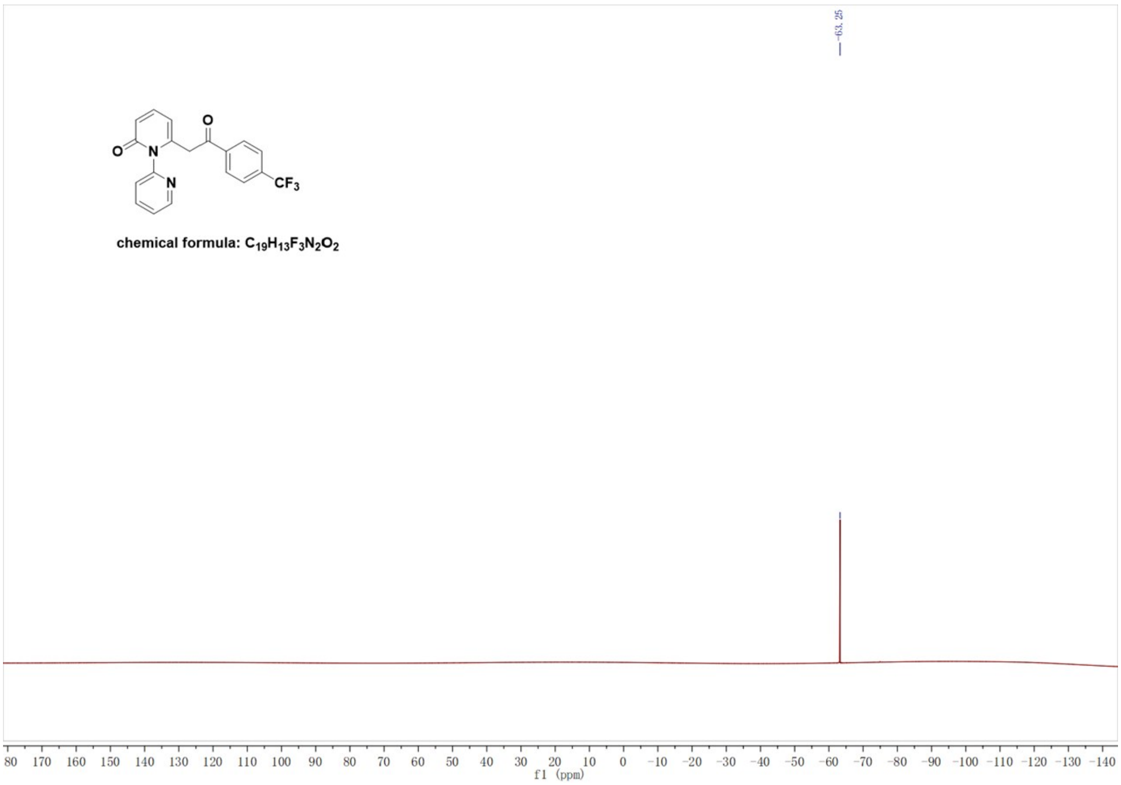

6-(2-oxo-2-(thiophen-2-yl)ethyl)-2*H*-[1,2'-bipyridin]-2-one (3ao)

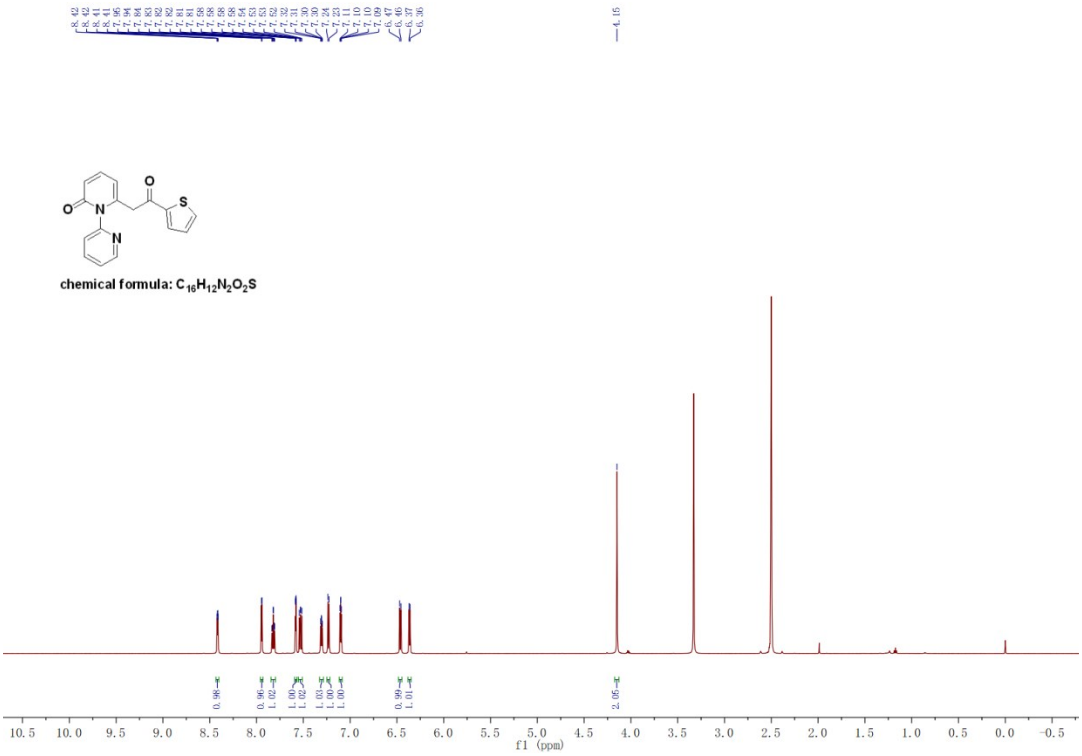

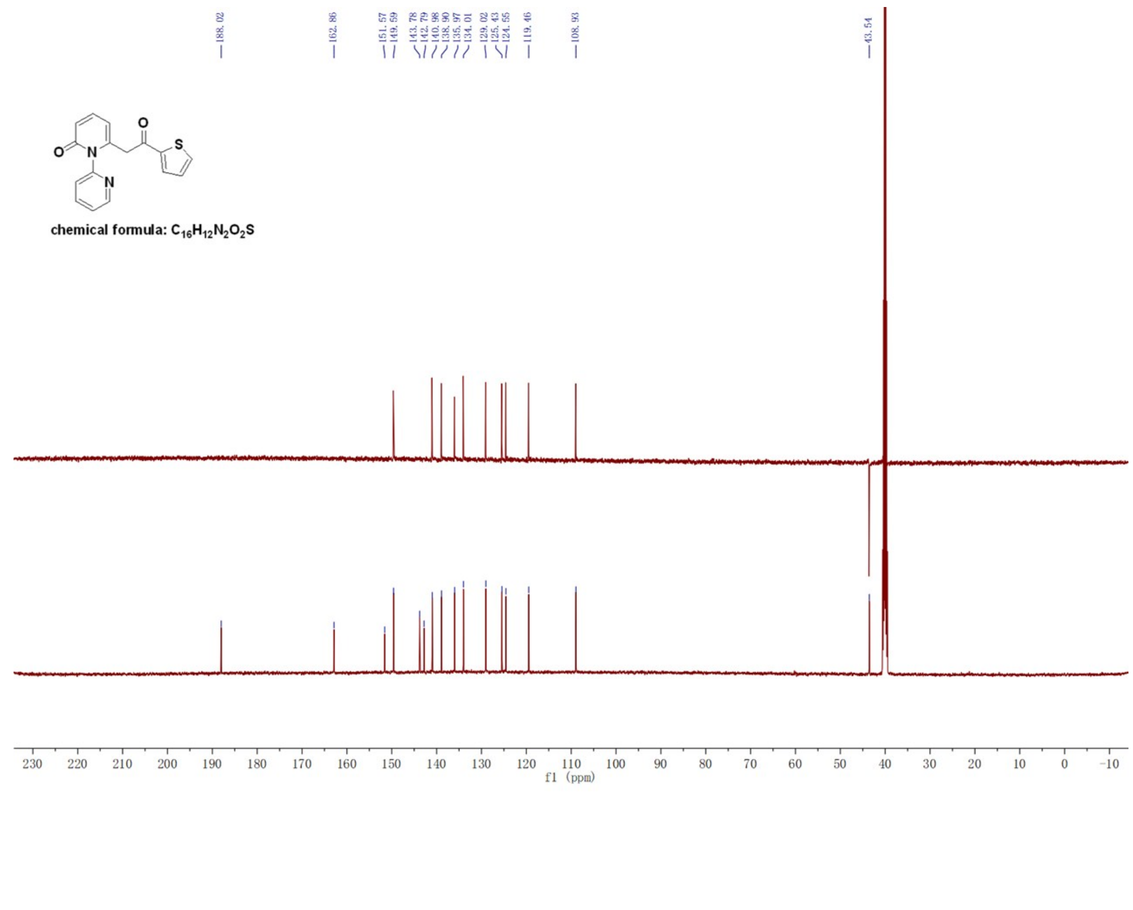

6-(3-methyl-2-oxobutyl)-2*H*-[1,2'-bipyridin]-2-one (3ap)

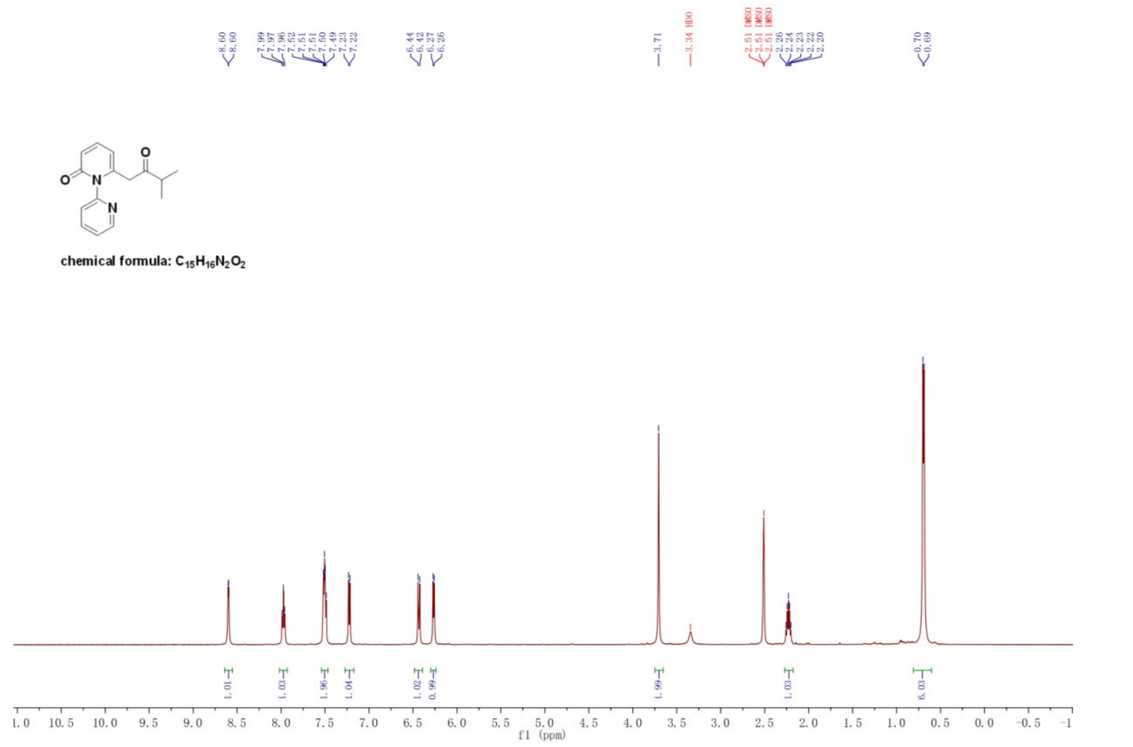

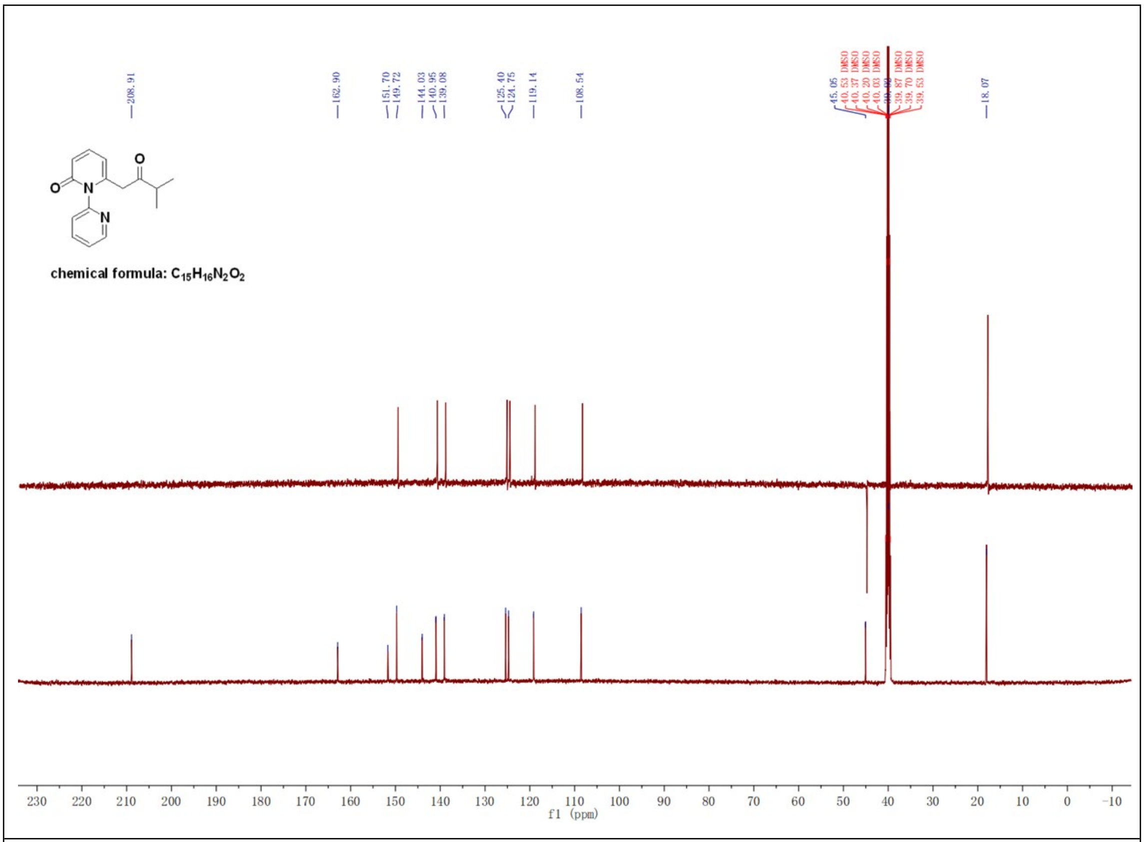

6-(3,3-dimethyl-2-oxobutyl)-2*H*-[1,2'-bipyridin]-2-one (3aq)

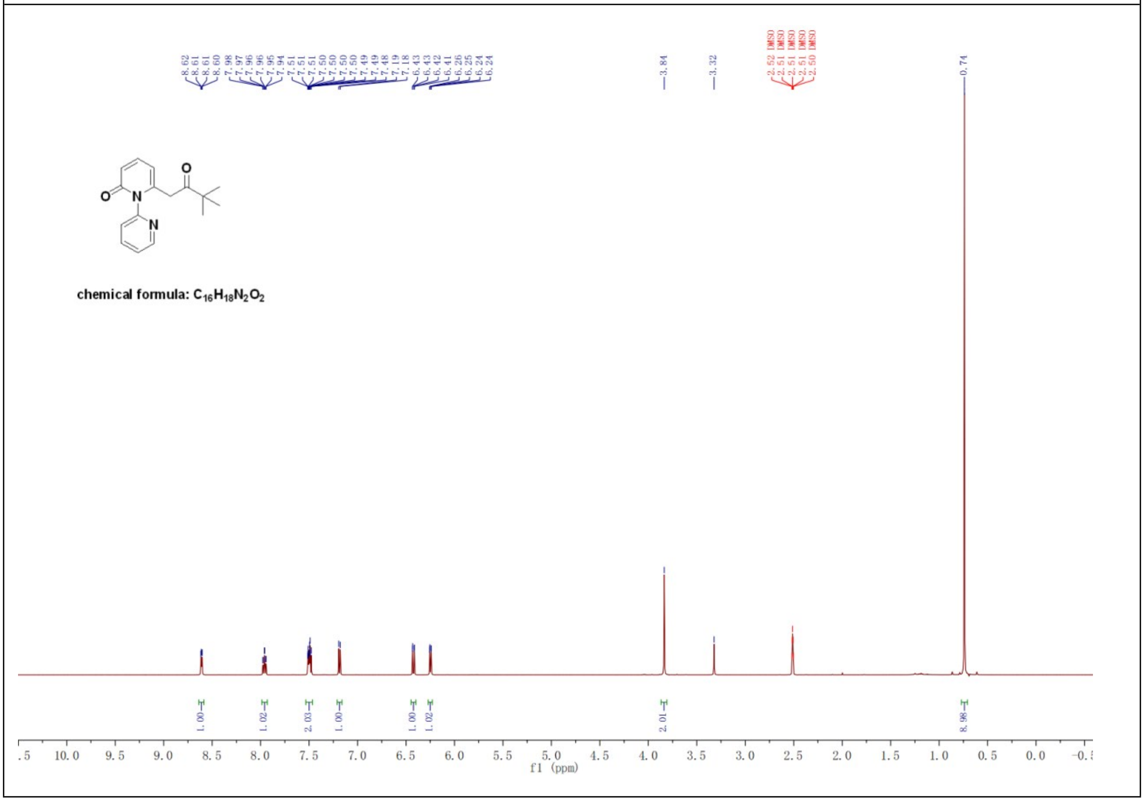

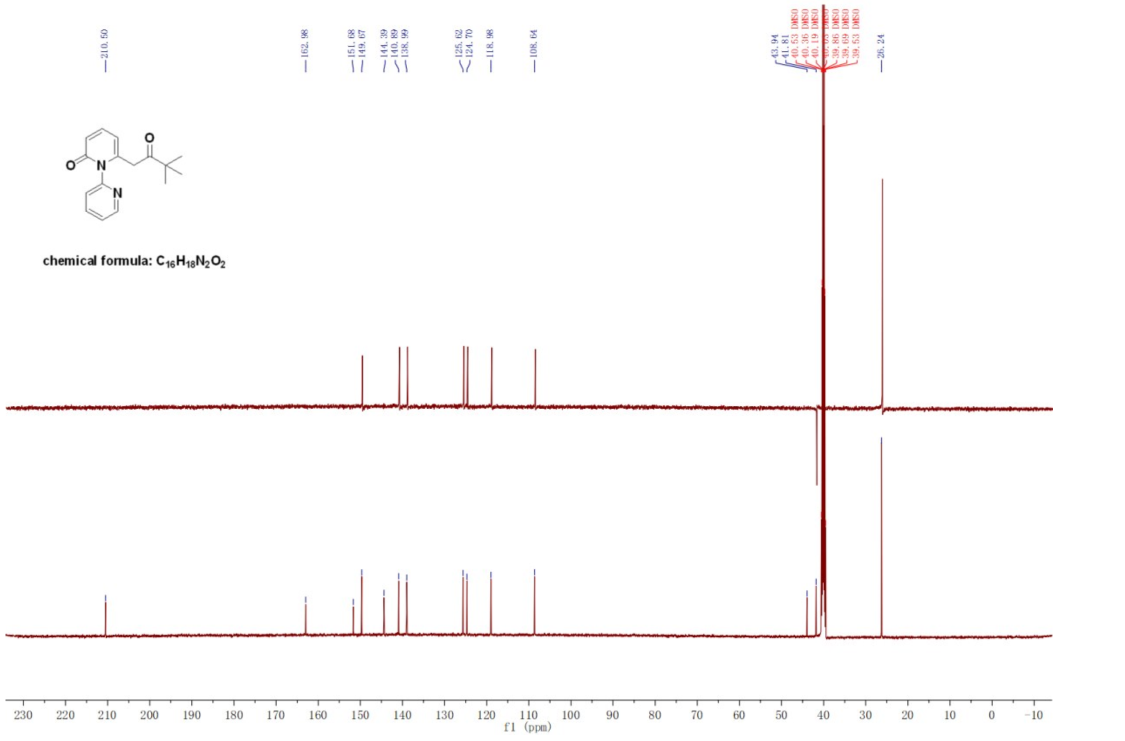

6-(2-oxo-2-phenylethyl)-1-(pyridin-2-yl)piperidin-2-one (4aa)

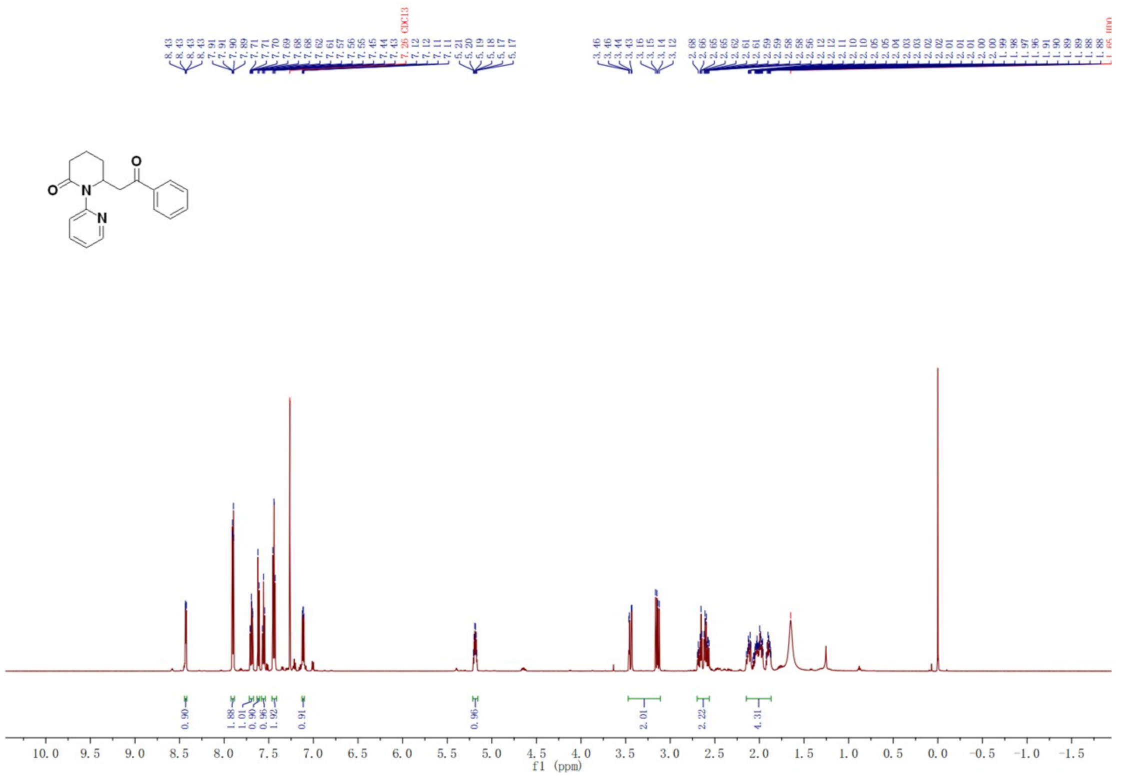

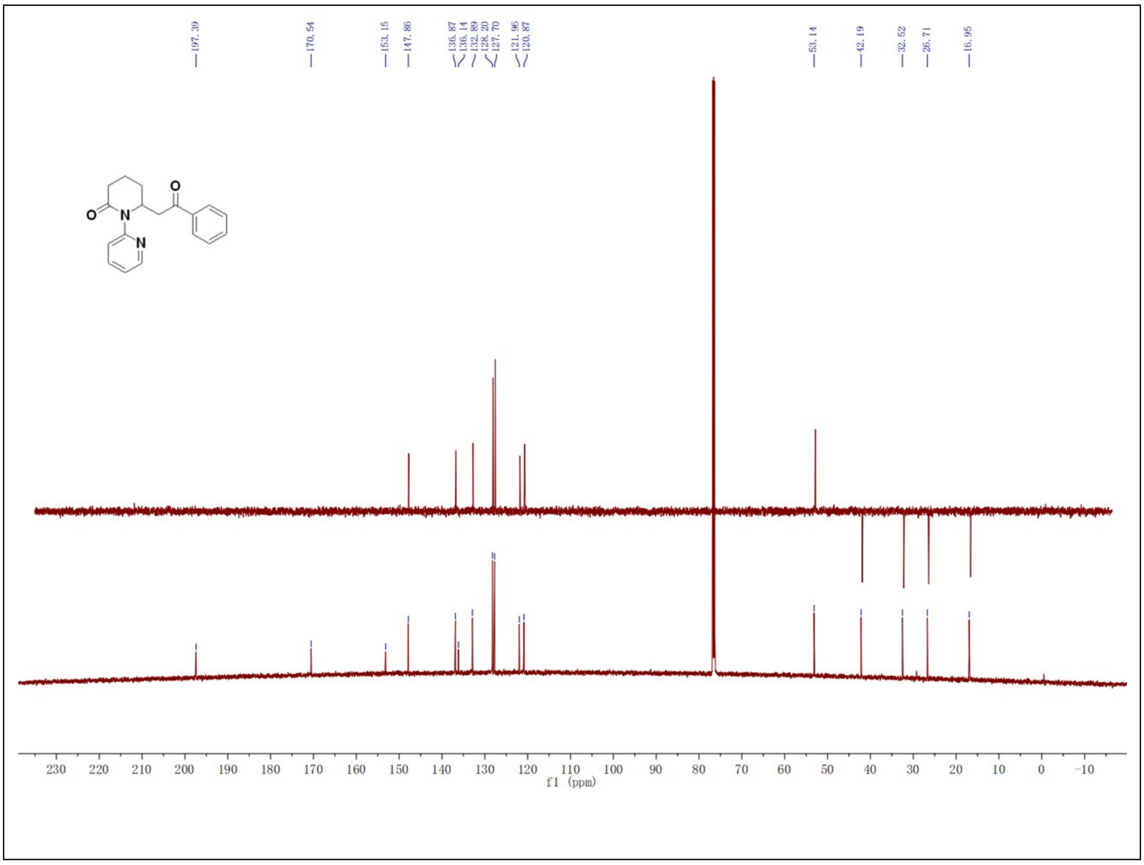
197.39
170.54
153.15
147.86
136.87
136.14
132.89
128.20
127.70
121.96
120.87
53.14
42.19
32.52
26.71
16.95
O
O
N
N
230 220 210 200 190 180 170 160 150 140 130 120 110 100 90 80 70 60 50 40 30 20 10 0 −10
f1 (ppm)
